# Cationic Intrinsically Disordered Antimicrobial Peptides (CIDAMPs) Represent a New Paradigm of Innate Defense with a Potential for Novel Anti-Infectives

**DOI:** 10.1038/s41598-019-39219-w

**Published:** 2019-03-04

**Authors:** Ties Latendorf, Ulrich Gerstel, Zhihong Wu, Joachim Bartels, Alexander Becker, Andreas Tholey, Jens-Michael Schröder

**Affiliations:** 1 Department of Dermatology, University-Hospital Schleswig-Holstein, Campus Kiel, Kiel, Germany; 2 Institute for Experimental Medicine–AG Systematic Proteomics & Bioanalytics, Kiel University (CAU), Kiel, Germany; 3 Present Address: Institute of Biochemistry and Cell Biology, Zhejiang University of Science and Technology, 310023 Hangzhou, China

## Abstract

In the search for potential mechanisms underlying the remarkable resistance of healthy skin against infection by soil bacteria like *Pseudomonas* (*P*.) *aeruginosa* we identified fragments of the intrinsically disordered protein hornerin as potent microbicidal agents in the stratum corneum. We found that, independent of the amino acid (AA)-sequence, any tested linear cationic peptide containing a high percentage of disorder-promoting AA and a low percentage of order-promoting AA is a potent microbicidal antimicrobial. We further show that the antimicrobial activity of these cationic intrinsically disordered antimicrobial peptides (CIDAMPs) depends on the peptide chain length, its net charge, lipidation and environmental conditions. The ubiquitous presence of latent CIDAMP sources in nature suggests a common and yet overlooked adapted innate disinfection system of body surfaces. The simple structure and virtually any imaginable sequence or composition of disorder-promoting AA allow the generation of a plethora of CIDAMPs. These are potential novel microbicidal anti-infectives for various bacterial pathogens, including *P. aeruginosa*, methicillin-resistant *Staphylococcus aureus* (MRSA) and fungal pathogens like *Candida albicans* and *Cryptococcus neoformans*.

## Introduction

The surface of human skin is an intricate habitat for numerous microbes, that colonize at different areas in an anatomical-site specific manner^1^ with remarkable and unexpected temporal stability^2^. *Proteobacteria* were identified as the dominant division while the genus *Pseudomonas* was identified as the largest phylotype of human skin^3^. *Pseudomonas spp* are very frugal soil- and waterborne bacteria that thrive under moist conditions. In addition, *Pseudomonas* spp are commonly found on healthy human skin surfaces, primarily in areas with an adequate content of moisture and humidity. Besides mucosal epithelia, suitable areas seem to be the lumen and ducts of eccrine sweat glands of skin. Although Pseudomonads are highly abundant on human skin^3^, *Pseudomonas* (*P*.) *aeruginosa* is an opportunistic pathogen capable to infect skin when the cutaneous barrier is disturbed, such as in toe web infections^4^ or hot tub folliculitis^5^. *P. aeruginosa* is a major cause of morbidity and mortality^6^ in particular under conditions where the cutaneous barrier is completely missing, e.g. in burn wounds. It is therefore astonishing that *P. aeruginosa*-infections of healthy skin rarely occur, suggesting that antimicrobial factors of healthy person’s stratum corneum actively control *P. aeruginosa* growth.

A potential source of *P. aeruginosa*-directed antimicrobials could be eccrine sweat glands, which are producing the sweat-specific antimicrobial peptide (AMP) dermcidin^7^. Dermcidin shows antimicrobial activity against a range of bacteria, however, only little activity against *P. aeruginosa*^8^. This suggests that sweat glands and epidermis produce additional factors, which control *Pseudomonas spp*. Therefore, we surmise that yet unknown *P. aeruginosa*-targeting AMPs may limit its pathogenic activity at the healthy skin surface.

## Results

### Stratum corneum extracts contain AMPs with activity at acidic conditions

**Figure 1:**
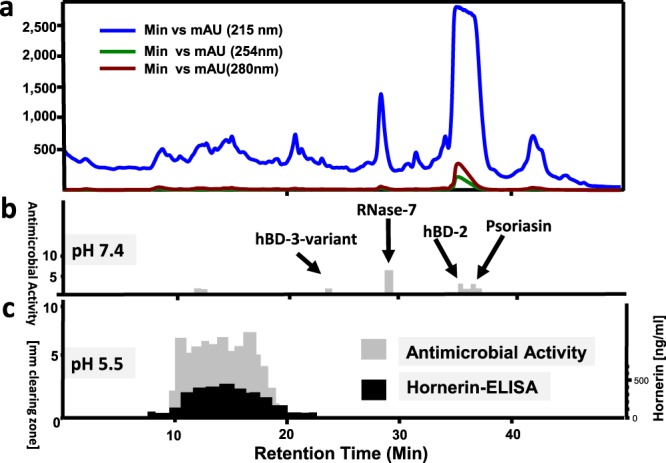
*P. a*.-inhibitory antimicrobials in stratum corneum. (**a)** reversed-phase-HPLC of heparin-bound proteins of an acidic stratum corneum extract. (**b)** radial-diffusion antimicrobial assay (RDA) of HPLC fractions for *P. a*. ATCC 10145 inhibitory activity at pH 7.4. (**c)** RDA of HPLC fractions for *P. a*. inhibitory activity at pH 5.5 and test for immunoreactive HRNR. *P. a*.-inhibitory activity at pH 7.4, corresponding to known skin antimicrobial peptides like RNase-7, an hBD-3 variant, hBD-2 and psoriasin is shown in (**b**). Results are representatives of three (**b,c**) independent experiments.

**Figure 2:**
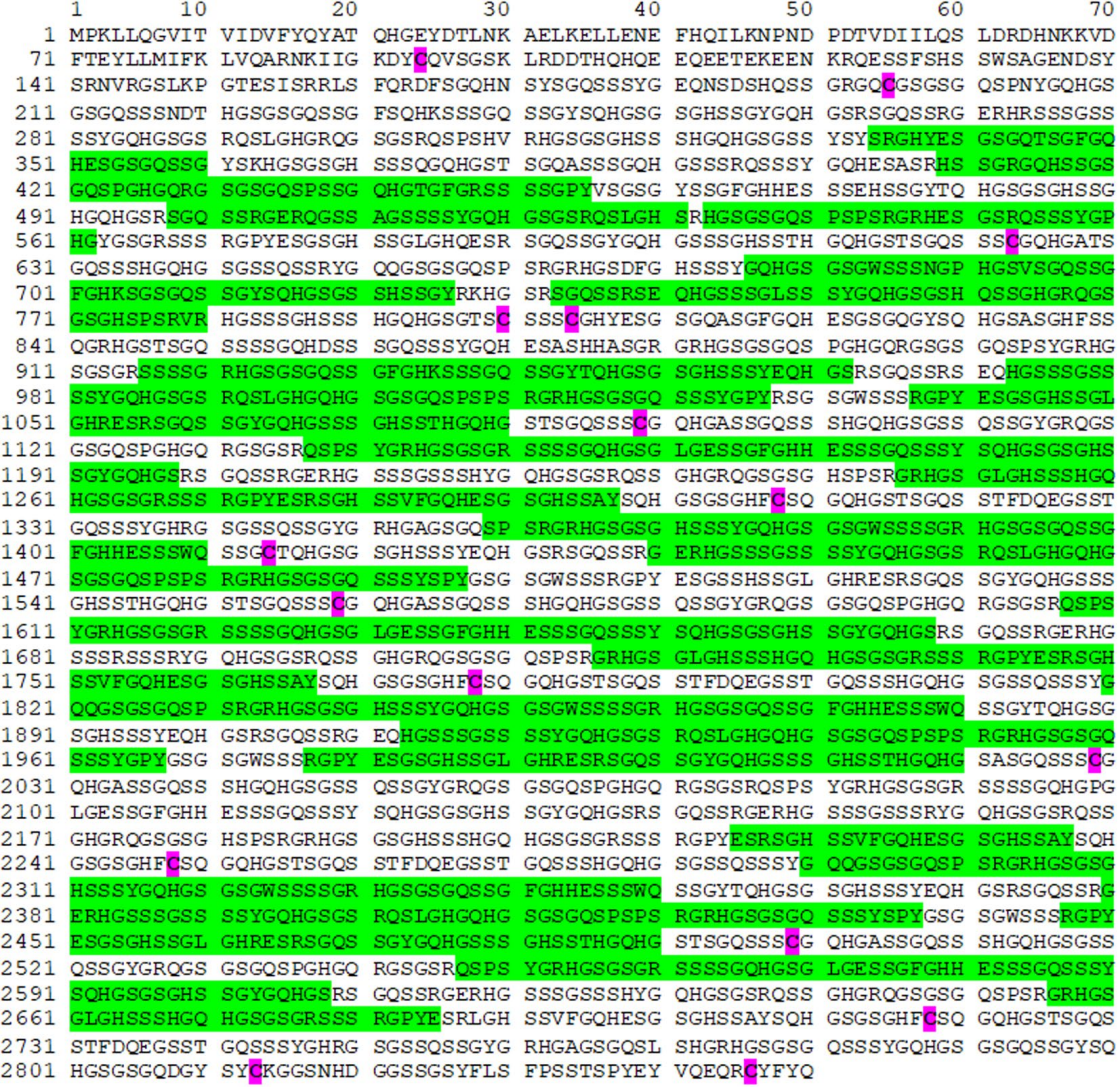
Sequence coverage of HRNR in pooled healthy individuals stratum corneum extracts. Identified peptides are highlighted in green. Note the absence of identified HRNR peptides in domains containing Cys residues (highlighted in magenta).

We hypothesized that skin-derived soil- and waterborne bacteria-controlling compounds should be present in foot skin, an area with high soil contact. The sole of the foot contains a high number of sweat glands. Further, extracts of plantar *stratum corneum* are a rich source of *Escherichia coli* (*E. coli*) - and *Staphylococcus aureus (S. aureus)*-targeting AMPs^9^. Such extracts have not yet been systematically analyzed for compounds inhibiting other bacteria, e.g. for *P. aeruginosa*-targeting AMPs. Since a healthy skin surface is acidic, the commonly used antimicrobial assay system^10^ was adapted to this pH. Further, a low ion strength buffer at pH 5.5 containing only 1% tryptic soy bean broth (TSB) as nutritional additive was used; this deviates from the standard procedures for antibiotic sensitivity testing, outlined by the Clinical and Laboratory Standards Institute (CLSI)^10^. Analyses of cationic stratum corneum proteins, separated by reversed-phase (RP) high performance liquid chromatography (HPLC) revealed a broad peak containing antimicrobial active compounds (Fig. 1c). Notably, unlike well characterized skin-derived peptide antibiotics, such as beta-defensins, RNase-7, psoriasin and LL-37^11^, this inhibitory activity is not observable when using a standard antimicrobial assay (Fig. 1b). The antimicrobial active compounds elutes in many adjacent HPLC-fractions at retention times similar to those of a high number of cationic and very polar polypeptides (Fig. 1c). These co-elute with fragments of the S100-fused-type-protein (SFTP) hornerin (HRNR), a 282 kDa protein (2,850 amino acids (AA))- containing approximately 95% Gly/Ser-rich quasi-repeat domains^12^. This suggests that HRNR peptides are *P. aeruginosa*-targeting antimicrobials. Peptidomic analyses of pooled *P. aeruginosa*-cidal activity- and HRNR-containing HPLC-fractions of heparin-bound heel stratum corneum proteins reveal 122 HRNR-peptides, of which 117 are unique for HRNR (Fig. 2, Supplementary Fig. 1). Despite the high number of identified peptides, the overall HRNR sequence coverage is only 40% (Fig. 2). Interestingly, most of the non-identified sequence stretches of HRNR contain at least one cysteine residue (Fig. 2), which might have been caused either by a reduced detectability of Cys-containing peptides in LC-MS/MS analysis^13^ or have been masked by the presence of posttranslational modifications, e.g. at Cys residues.

Analysis of the N- and C-termini of the identified peptides hints for multiple proteases potentially being involved in HRNR degradation. Analyses of P1 positions (Supplementary Fig. 2) may point towards a possible role of stratum corneum proteases such as diverse kallikreins^14^ and profilaggrin endoproteinase 1 (PEP1). The latter is a profilaggrin processing enzyme that hydrolyses peptides derived from insoluble profilaggrin, which results in its partial solubilization. Cleavage sites identified in several peptides are consistent with HRNR *in vivo* processing sites and with a role in dispersal of HRNR and profilaggrin during terminal differentiation^15^. Further, the appearance of N- and C-terminal truncated HRNR peptides suggests that both, endo- and exoproteases, may contribute to the characteristic HRNR fragmentation pattern (Supplementary Fig. 1).

### HRNR-polypeptides are *P. aeruginosa*-cidal Antimicrobials

*P. aeruginosa* ATCC10145-cidal activity was tested for several recombinant HRNR fragments (rHRNR_1075–1172_, rHRNR_2638–2684_, rHRNR_2656–2684_, rHRNR_2727–2850_, rHRNR_2591–2684_, rHRNR_2591–2644_ and rHRNR_2576–2707_) and the fusion protein rSUMO3-HRNR_2591–2684_ (Table 1, Supplementary Fig. 3, Supplementary Table 1) in a colony-forming unit (CFU) assay^10^ at pH 5.5. This assay reveals a *P. aeruginosa*-cidal activity at nanomolar concentrations. Interestingly, higher sensitivity of *P. aeruginosa* is observed in the absence of TSB (Supplementary Fig. 3e–g). In addition, HRNR polypeptides are also antimicrobials with lower potency for *E. coli* ATCC 11775 and the yeast *Candida albicans* (*C. albicans*) ATCC 24433. In contrast, *S. aureus* ATCC 6538 is sensitive only in the absence of TSB (Supplementary Fig. 3e–g). Since *P. aeruginosa*-sensitivity might depend on pH and/or additives in the test medium, *P. aeruginosa*-cidal activity of SUMO3-HRNR_2591–2684_ was tested in the presence of TSB, Luria Broth (LB) or no additive, at pH 7.3 and 5.5. We found a dose-response curve shift towards lower potency and efficacy by both, increased pH and addition of TSB or LB (Supplementary Fig. 4).

**Table 1:** Various recombinant HRNR-fragments and SUMO3-HRNR-fusion proteins are antimicrobials.

Name	Net charge^b^	LD90 (µg/ml) *P. aeruginosa* ATCC 10145	LD100 (µg/ml) *P. aeruginosa* ATCC 10145	LD90 (µg/ml) *E. coli* ATCC 11775	LD100 (µg/ml) *E. coli* ATCC 11775	LD90 (µg/ml) *C. albicans* ATCC 24433	LD100 (µg/ml) *C. albicans* ATCC 24433
rHRNR_1075–1172_	+14	6.25	>100	6.25	>100	1.6	12.5
rHRNR_2638–2684_	+10	0.39	12.5	3.1	>100	6.3	>100
rHRNR_2656–2684_	+6	0.78	12.5	3.1	>100	6.3	>100
rHRNR_2727–2850_	+9	2.1	17.1	0.28	>100	8.6	137
rHRNR_2591–2684_ -SUMO3	+22	0.2	1.6	0.8	>100^c^	0.8	4.8
rHRNR_2576–2707_^a^	+21	0.035	0.275	0.55	>100	0.55	>140
rHRNR_2591–2684_^a^	+10	0.025	0.1	0.2	>100	0.39	6.25
rHRNR_2591–2684_ -SUMO3^a^	+22	0.025	0.1	0.05	0.2	—	—

P. a. sensitivity has been tested in a CFU assay in 10 mM NaP/1% TSB, pH 5.5 or. ^a^In 10 mM NaP/0.25% glucose/pH 5.5. ^b^At pH 5.5. ^c^Antimicrobial paradox. For complete dose-response curves see Supplementary Fig. 3 and for AA-sequences see Supplementary Table 3. LD90; LD100: lethal dose killing 90% or 100% of the inoculum. Data shown are representatives (n = 2).

To explore whether short HRNR fragments are antimicrobials, we screened chemically synthesized peptides of the repeat domain HRNR_2591–2684_ for *P. aeruginosa*-cidal activity. Peptides HRNR_2606–2628_ (HR1-11), HRNR_2656–2684_ (HR1-17) and HRNR_2656–2677_ (HR1-18) were identified as the most active *P. aeruginosa-cidal* compounds (Table 2). HR1-18 is active only at a pH ≤6.5 (Supplementary Fig. 5) and in the absence of TSB (Supplementary Table 2).

**Table 2:** Identification of potent *P. a*. ATCC 10145-cidal HRNR_2591–2684_ peptide fragments.

AA-sequence	Net charge*	Name	n	LD90 (µg/ml)	LD100 (µg/ml)
HGSRSGQSSRGERHGSSSGSSSH	+5	HR 1-11, HRNR_2606–2628_	2	0.075	2.35
GRHGSGLGHSSSHGQHGSGSGRSSSRGPY	+7	HR 1-17, HRNR_2656–2684_	2	0.038	0.15
GRHGSGLGHSSSHGQHGSGSGR	+6	HR 1-18, HRNR_2656–2677_	7	0.038	0.15

Test condition: 10 mM NaP, pH 5.5 and 0.25% glucose. *At pH 5.5. The results shown are representatives. n: number of experiments.

### Structural requirements for antimicrobial activity

**Figure 3:**
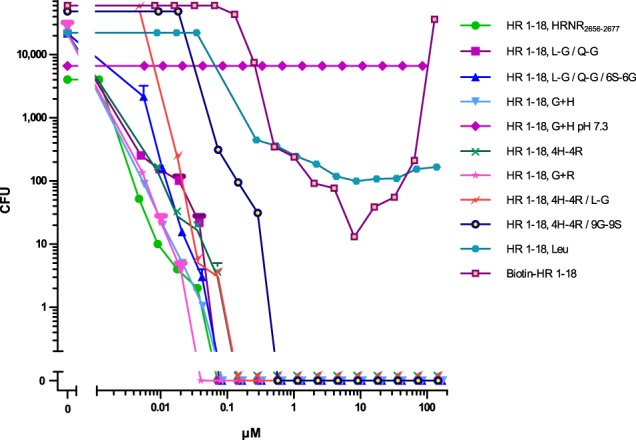
*P. a*. ATCC 10145-killing by HR1–18 and its mutants and variants. *P. a*. ATCC 10145-cidal activity in 10 mM NaP/0.25% glucose/pH 5.5, was examined for various mutants and variants of HR1-18 (AA-sequences see Supplementary Table 6). Data shown are representatives (n = 2) or mean ± s.e.m. of HR1-18, L-G/Q-G/6S-6G (n = 3); HR1-18, L-G/Q-G (n = 3) and HR1-18, G + R (n = 3).

To elucidate structural requirements for *P. aeruginosa*-cidal activity, several HR1-18 variants were tested for antimicrobial activity at pH 5.5: Replacing Leu, Gln or all Ser by Gly or replacing the four His by Arg (HR1-18, 4H-4R) does not alter antimicrobial potency and efficacy (Fig. 3, Supplementary Table 3). Replacing all Arg by His and all non-His amino acids (AA) by Gly (HR1-18, G + H) or replacing all His by Arg and all non-Arg AA by Gly (HR1-18, G + R) alters antimicrobial potency and efficacy marginally. When all Arg and all His are replaced by Gly in HR1-18, antimicrobial activity is abolished. Interestingly, replacement of the N-terminal Gly by the hydrophobic Leu in HR1-18 (HR1-18-Leu) causes an increase of its LD90 and LD100 (Supplementary Table 3). Derivatization of the N-terminal Gly by a bulky biotinyl-group alters the shape and causes a shift of the dose-response curve towards higher concentrations of Biot-HR1-18 (Fig. 3). Curiously, bactericidal activity of this variant now reveals a unique “antibiotic paradox” - an increase of bacterial survival with increased concentration of this peptide (Fig. 3), which has been also observed for some antibiotics^16^.

In a further site-directed mutation analysis of the short HRNR fragment HR1-17 we replaced different single Arg by Gly. Although replacement reduces the net charge, the LD100 and LD90 of *P. aeruginosa*-cidal activity does not markedly change (Supplementary Fig. 6, Supplementary Table 4). Replacement of all four His by Gly, however, reduces the LD90 by a factor of approximately 1,000 and raises the LD100 to concentrations >300 µg/ml. This suggests that the number of cationic AA in HRNR-derived peptide-fragments determines the potency of its antimicrobial activity. As expected from known His-rich AMPs^17^, and seen in HRNR_2591–2684_, also HR1-18 shows antimicrobial activity only at acidic conditions (Supplementary Fig. 5).

### HRNR-derived AMPs are enriched in disorder-promoting AA

Peptide HR1-18 and its variants feature a high percentage of disorder-promoting AA (Gly, His, Ser, Arg, Gln, Pro), a very low percentage of order-promoting AA (only Leu) and a high positive net charge at pH 5.5. This suggests that a peptide, composed primarily of disorder-promoting AA with a sufficient number of cationic AA, could be a *P. aeruginosa*-cidal peptide antibiotic. For example, a 22-mer peptide composed only by Gly and His, generating the peptide Gly_16_-His_6_ (HR1-18, G + H), is a highly potent *P. aeruginosa*-cidal AMP at pH 5.5 (Fig. 3, Supplementary Table 3).

**Figure 4:**
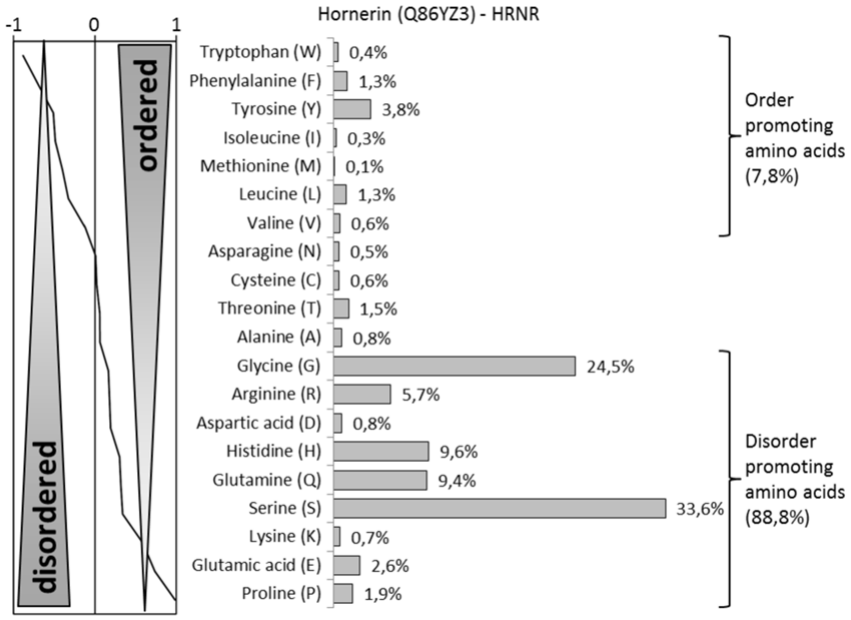
AA-composition of HRNR. HRNR consists in nearly 90% of disorder-promoting AA and only 8% order-promoting AA, which are exclusively located within the N-terminal S100-domain and the C-terminus.

Unlike amphipathic antimicrobial peptides such as defensins and cathelicidins^18^, HRNR is a highly cationic, hydrophilic protein. It is rich in disorder-promoting AA with a very low content of order-promoting AA (Fig. 4). Evaluation of the intrinsic disorder predisposition by several computational tools predicts that this protein consists of approximately 95% functional disordered regions with a low percentage of structure-defining AA (which are limited to the N-terminal S100-domain (AA 1-100) and the C-terminus)(Supplementary Fig. 7), representing an “intrinsically disordered protein, IDP”.

If cationic IDPs in general are antimicrobials, one would expect that the AA composition rather than structure-defining characteristics, such as the AA-sequence or chirality, defines the antimicrobial activity of HRNR-peptides. This would challenge the structure-function paradigm, which reflects an apparent corollary of the “Anfinsen’s dogma”^19^ stating that folding into a well-defined structure is a crucial requirement for a protein exerting defined biological activity. The observation that a few isolated synthetic peptides from denaturated proteins were found to be antimicrobial peptides even when their original AA-sequence was reversed or randomized, led to the assumption that also AMPs lacking structural requirements exist^20^.

**Figure 5:**
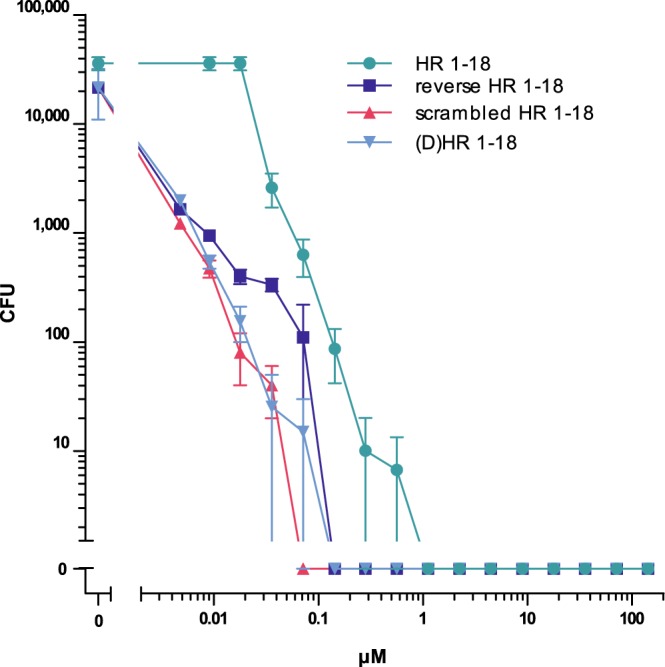
Antimicrobial activity of HR1-18 depends not on structure-defining parameters. Sensitivity of *P. a*. ATCC 10145 towards HR1-18, a retro-analog with the reversed AA-sequence of HR1-18 (reverse HR1-18), a peptide with a scrambled AA-sequence of HR1-18 (scrambled HR1-18) and an enantiomeric HR1-18 peptide where all L-amino acids have been replaced by D-amino acids ((D)HR1-18) were tested in 10 mM NaP/0.25% glucose/pH 5.5. AA-sequences are shown in Supplementary Table 5. Data are mean ± s.e.m. (n = 3).

To test this dogma-challenging hypothesis, mutants with the reversed AA-sequence of HR1-18 (revHR1-18), a scrambled AA-sequence (scrHR1-18) and the enantiomeric form of HR1-18 containing D-AAs instead of the natural L-AAs were investigated. For all these HR1-18 variants no reduction of its antimicrobial activity was found. Instead, LD90 and LD100 values are in a similar range as those of the wild-type HR1-18 (Fig. 5, Supplementary Table 5). This suggests that the AA-composition, but not structure-defining parameters such as hydrophobic AAs, AA-sequence or chirality, determine the antimicrobial activity of cationic IDPs. Hence, we propose to name them “Cationic Intrinsically Disordered Antimicrobial Peptides, CIDAMP”. If cationic IDP or “Intrinsically Disordered Protein Regions (IDPR)” of various origins, containing a high percentage of distinct disorder-promoting AA, are interlinked clusters of antimicrobials, tissues rich in cationic IDPR and hence potential CIDAMP-sources, could represent an important antimicrobial barrier and innate defense system in any organism.

IDPs and IDPRs represent a large class of proteins that are defined by conformational heterogeneity and lack of persistent secondary/tertiary structure. Nearly 30% of natural proteins in all organisms are IDPs or contain IDPRs^21^. Several major structural proteins expressed in the cornified envelope (CE) of human epidermis are enriched with cationic IDPRs. In addition to the SFTPs HRNR (Supplementary Fig. 7), filaggrin-2 (FLG-2), repetin (RPT), filaggrin (FLG), and “Late Cornified Envelope Proteins (LCEs)” such as LCE3B (Supplementary Fig. 8), many other proteins are predicted to represent IDPs or contain cationic IDPRs. In particular, proteins of the epidermal differentiation complex (EDC), which are all encoded on chromosome 1q21, are essential for epidermal differentiation and expressed in the outermost layers of the epidermis of healthy skin^22^.

### Cationic and Disorder-promoting AA-enriched Peptides are Antimicrobials

To test the hypothesis that in general IDP and IDPR peptide fragments with a net positive charge are *P. aeruginosa* ATCC 10145-cidal AMPs, peptides corresponding to defined parts of different repetitive domains in HRNR for *P. aeruginosa*-cidal activity were studied. All these cationic HRNR-peptides and variants are rich in the disorder-promoting AAs Gly, Ser, Gln, His and Arg (Supplementary Table 6). Short HRNR-derived peptides with a net positive charge ≤+3 mostly lack *P. aeruginosa*-cidal activity (Supplementary Table 6, Supplementary Fig. 9). An increase of the peptide chain length together with an increase of the net positive charge affects both, potency and efficacy of its *P. aeruginosa*-cidal activity (Supplementary Table 6, Supplementary Fig. 9). Interestingly, in some cases, substitution of a single AA in a CIDAMP (e.g. Leu by Ser in HR1-18 (HRNR_2656–2677_ vs HRNR_2186–2207_), or replacing Ser by Gly (HRNR_2422–2450_ vs HRNR_1952–1980_) or replacing N-terminal Gly in HRNR_2656–2677_ by Leu (generating HR1-18-Leu) (Supplementary Table 6) causes an increase of the LD90 and LD100 of *P. aeruginosa*-cidal activity.

We then asked whether peptide fragments of other IDPRs are CIDAMPs. Selected peptides of the SFTPs FLG-2, FLG and RPT, which unlike HRNR are rich in Thr and Gln and have a low Gly content, can eradicate *P. aeruginosa* ATCC 10145 (Supplementary Table 7, Supplementary Fig. 10).

In summary, the bactericidal action of *P. aeruginosa* ATCC 10145-killing CIDAMPs depends primarily upon the presence of a high percentage of disorder-promoting AA, the number of positively charged AA resulting in a positive net charge, and the length, but not the AA sequence of the peptide. Our data corroborate the assumption that the potent, nM concentration range, *P. aeruginosa* ATCC 10145-cidal activity of CIDAMPs requires a net positive charge of +4 or higher under conditions mimicking the microbial skin environment, i.e. pH 5.5 and a limited availability of nutrients.

### Selected HRNR-CIDAMPs Exhibit *Staphylococcus aureus*-cidal activity

**Figure 6:**
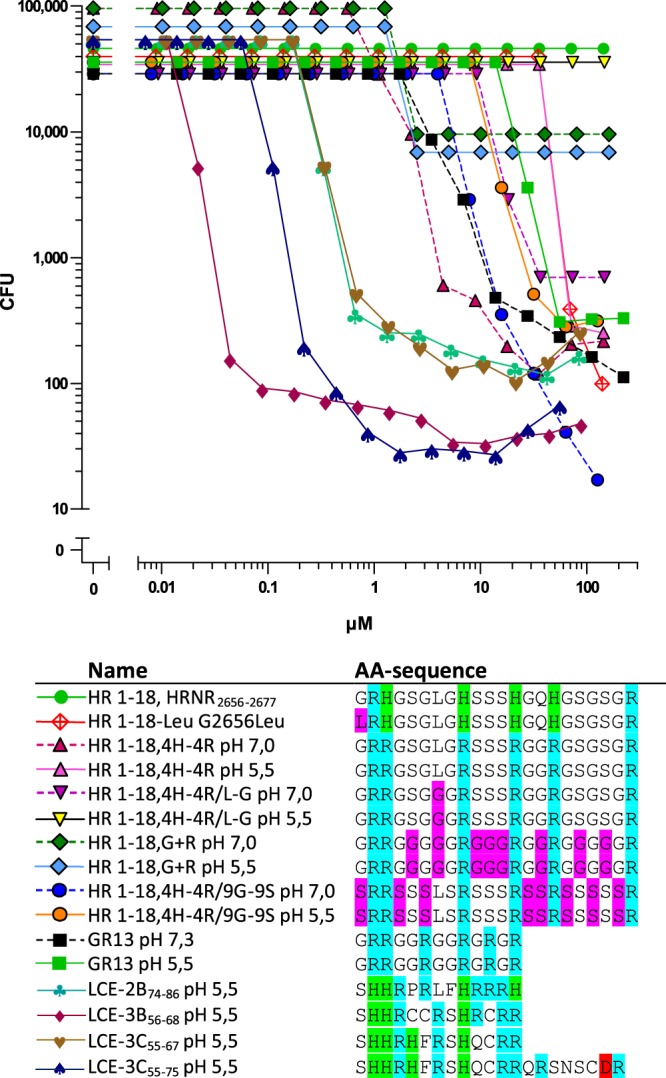
CIDAMPs with *S. a*.-cidal activity. Sensitivity of *S. a*. ATCC 6538 towards peptides was tested in 10 mM NaP/0.25% glucose/pH 5.5 unless otherwise indicated. Residues cationic at pH 7.0, cationic only at acidic pH and anionic are colored blue, green and magenta, respectively. Note a dose-response curve shift towards higher potency with increased Ser residues in HR1-18,4H-4R/9G-9S). All data shown are representatives (n = 2).

The identified CIDAMPs are highly potent *P. aeruginosa*-targeting peptide antibiotics. To investigate whether CIDAMPs are targeting also *S. aureus*, a major opportunistic skin pathogen, in particular in atopic dermatitis^23^, we first investigated *S. aureus*-sensitivity towards recombinant HRNR-polypeptides and found low potency *S. aureus*-cidal activity at pH 5.5 (Supplementary Fig. 3e–g). We then studied HRNR-derived short CIDAMPs. Whereas HR1-18 lacks *S. aureus*-bactericidal activity, this pathogen becomes susceptible towards a HR1-18- variant where all His are replaced by Arg (Fig. 6, Supplementary Table 8). Unexpectedly, this peptide is more potent at pH 7.0 than at pH 5.5. In addition, HR1-18 is converted towards a *S. aureus*-cidal AMP with very low potency when the N-terminal Gly is replaced by Leu. Strikingly, this peptide loses its potent *P. aeruginosa-cidal* activity, with a shift of the LD90 towards nearly 100-fold higher concentrations (Supplementary Table 6).

Replacing the nine Gly by Ser in HR1-18,4H-4R makes this HR1-18-mutant (HR1-18,4H-4R/9G-9S) slightly more potent at pH 5.5, but it retains *S. aureus*-cidal activity at pH 7.0 (Fig. 6, Supplementary Table 8). HR1-18,4H-4R is rich in Ser and Arg and contains a single Leu. When this single Leu in HR1-18,4H-4R is replaced by Gly (forming HR1-18,4H-4R/L-G), *S. aureus*-cidal activity is abolished, suggesting a possibly critical role for the presence of a low percentage of hydrophobic AA in *S. aureus* -killing CIDAMPs.

### Peptide fragments of Late-Cornified-Envelope Proteins, LCEs, are *S. aureus*-cidal CIDAMPs

Next, we tested the hypothesis that peptide fragments of other epidermal differentiation complex-derived IDPRs are presumptive *S. aureus*-cidal CIDAMP candidates: “Late-Cornified-Envelope proteins, LCEs” were selected as cationic IDPR-containing proteins (Supplementary Fig. 8). The peptide fragments LCE-2B_74–86_, LCE-3B_56–68_, LCE-3C_55–67_ and LCE-3C_55–75_ (Supplementary Fig. 11) were generated as possible LCE-derived CIDAMPs. A putative cleavage site (Leu as P1) of Cathepsin D (Peptidase Data Base MEROPS, *merops.sanger.ac.uk/*) was chosen as the N-terminus for all investigated LCE-peptide fragments. Cathepsin D has a pH optimum at acidic pH and is important for processing of the sweat antimicrobial peptide dermcidin^24^. The C-terminus of these LCE-peptides represents a potential tryptic cleavage site which might be targeted by several skin-derived kallikreins and other epidermal serine proteases^14^. These cationic IDPRs of the LCEs are unique in their AA-composition due to the presence of one to three Cys and/or one hydrophobic Phe, together with paired His-residues (Supplementary Fig. 11). We surmised that the presence of the hydrophobic Phe residue in some LCE-derived CIDAMPs could contribute to putative *S. aureus*-cidal activity, similar to that seen for C-terminal AMP end-tagging by Phe^25^ where it might act as membrane anchor^26^. We further speculated that these LCE-based CIDAMPs possibly contain, or can generate (e.g. by dimerization), putative His- and/or Cys-thiolate-based binding sites for transition metals like Zn^2+^, Mn^2+^ or Fe^2+^; such mechanisms are known for antimicrobial peptides and proteins like *S. aureus*-cidal calprotectin (S100A8/A9)^27^, the Cys-reduced form of α-defensin HD-5^28^ or the fungicidal Cys-reduced form of psoriasin (S100A7)^29^.

Unlike investigated HRNR-peptides these LCE-peptides are potent and efficient *S. aureus* ATCC 6538-directed CIDAMPs (Fig. 6, Supplementary Table 8). Among the four LCE-peptides tested, LCE-3B_56–68_, a three Cys residues-containing LCE-peptide, is, in its free thiolate form, the most potent *S. aureus*-killing CIDAMP (LD90: 44 nM).

In summary, HRNR-derived short CIDAMPs, rich in hydrophilic Gly or Ser and a positive net charge show poor *S. aureus*-cidal activity. Despite this, certain LCE-peptides, having a unique AA composition encompassing disorder-promoting AA together with some His and Cys-thiolate-residues (putatively used as transition metal-binding sites), and a few hydrophobic AA, represent the most potent *S. aureus*-cidal peptide antibiotics of human skin identified to date.

### S-palmitoylation affects antimicrobial spectrum of HRNR-peptides

In addition to the AA-composition covalent modification, e.g. biotinylation, can also modify the antimicrobial potency, efficacy, and the antimicrobial activity spectrum of CIDAMPs (Supplementary Table 6). This suggests that natural post-translational modification of CIDAMPs may also modulate their antimicrobial activity.

**Figure 7:**
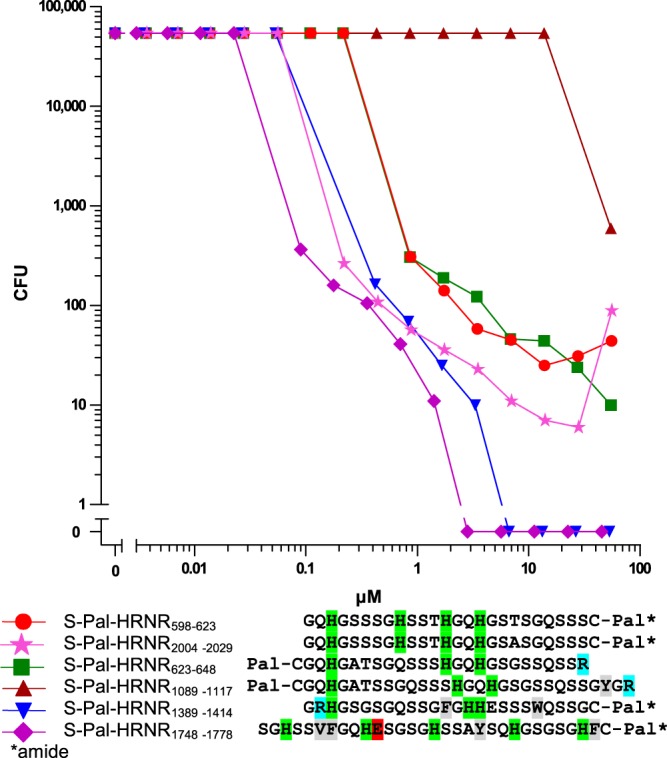
*S. a*. ATCC 6538 is sensitive towards selected S-palmitoylated HRNR-peptides. Sensitivity of *S. a*. ATCC 6538 towards peptides was tested in 10 mM NaP/pH 5.5/0.25% glucose. Residues cationic at pH 7, cationic only at acidic pH, anionic at pH 7 and hydrophobic are highlighted in blue, green, magenta and grey. Pal-C and S-Pal means an S-palmitoyl-Cys-residue. All data shown are representatives (n = 2).

An important post-translational modification is lipidation, in particular S-palmitoylation, which can make proteins lipophilic and thus soluble within lipid-rafts^30^. N-terminal palmitoylation is uncommon and mainly is formed by an acyl-transfer from lipids when peptides contain an N-terminal Gly and are incorporated in a lipid matrix^31^. Palmitoylation at cysteine-thiols (S-palmitoylation) is widely distributed^32^. N-palmitoylation of ultra-short amphipathic antimicrobial peptides has been shown to increase *S. aureus* sensitivity^33^. S-palmitoylated HRNR-fragments were identified in lipid rafts^34^. Therefore we hypothesized that S-palmitoylated HRNR peptides could be potent staphylocidal lipopeptides. A total of six potentially S-palmitoylated peptides are predicted to occur naturally from within the IDPR or HRNR, with S-palmitoylation possible at any of the 12 Cys residues within these (Fig. 2). Six S-palmitoylated (S-Pal) HRNR-peptides (S-Pal-HRNR_598–623_, S-Pal-HRNR_623–648_, S-Pal-HRNR_1089–1117_, S-Pal-HRNR_1389–1414_, S-Pal-HRNR_1748–1778_ and S-Pal-HRNR_2004–2029_) were tested for their activity against *S. aureus*. With the exception of S-Pal-HRNR_1089–1117_, all S-Pal-HRNR-peptides revealed potent staphylocidal activity (Fig. 7, Supplementary Table 9).

In summary, S-palmitoylation of Cys-containing HRNR-derived peptide fragments can generate potent *S. aureus*-cidal CIDAMPs.

### HRNR-derived CIDAMPs are Antimicrobials for Various Microorganisms

We then sought to determine if peptide fragments of rHRNR_2591–2684_ are broad spectrum antimicrobials. Two peptides, HR1-17 (HRNR_2656–2684_) and HR1-18 (HRNR_2656–2677_), were tested for activity against a selection of distinct microorganisms (Tables 3 and 4). When assayed in TSB-free medium, both peptides are inactive against the Gram-positive bacteria *S. aureus* ATCC 6538*, Streptococcus pneumoniae* ATCC 33400 and *Streptococcus pyogenes* ATCC 12344, except *Enterococcus faecium* DSM 2146. In addition, some strains of Gram-negative bacteria (*E. coli* ATCC 11775*, Proteus mirabilis* ATCC 21100*, Burkholderia cepacia* ATCC 25416) are also not susceptible. However, *Acinetobacter baumannii* ATCC 19606 is killed by HR1-18 (LD90: 1.18 µg/mL) and both peptides show activity against *C. albicans* (LD90: 18.8 µg/mL).

**Table 3:** Antimicrobial activity spectrum of HR 1-18 (HRNR_2656–2677_)*.

Microbe	n	LD90 (µg/ml)	LD100 (µg/ml)	Conditions
*Staphylococcus aureus* ATCC 6538	1	>300	>300	pH 5.5, 0.25% glucose
*Streptococcus pneumoniae* ATCC 33400	1	>300	>300	pH 6.5, 0.25% glucose
*Enterococcus. faecium* DSM 2146	2	150	>300	pH 5.5, 0.25% glucose
*Streptococcus pyogenes* ATCC 12344	1	>300	>300	pH 6.0, 0.25% glucose
*Pseudomonas aeruginosa* ATCC10145	7	0.038	0.3	pH 5.5, 0.25% glucose
*Pseudomonas aeruginosa* ATCC10145	3	0.15	2.35	pH 5.5, 1% TSB
*Acinetobacter baumannii* ATCC 19606	1	1.18	37.5	pH 6.0, 0.25% glucose
*Escherichia coli* ATCC11775	1	>300	>300	pH 5.5, 1% TSB
*Proteus mirabilis* ATCC 21100	1	>300	>300	pH 6.0, 0.25% glucose
*Candida albicans* ATCC 24433	2	18.8	150	pH 5.5, 0.25% glucose
*Candida albicans* ATCC 24433	2	150	>300	pH 5.5, 1% TSB
*Burkholderia cepacia* ATCC 25416	1	>300	>300	pH 5.5, 0.25% glucose

*AA-sequence: GRHGSGLGHSSSHGQHGSGSGR. CFU were examined in 10 mM NaP at indicated pH in the presence or absence of additives. All data shown are representatives. N: number of experiments.

**Table 4:** Antimicrobial activity spectrum of HR 1-17 (HRNR_2656–2684_)*.

Microbe	n	LD90 (µg/ml)	LD100 (µg/ml)	Conditions
*Staphylococcus aureus* ATCC 6538	2	>300	>300	pH 5.5, 1% TSB
*Pseudomonas aeruginosa* ATCC10145	2	0.075	0.3	pH 5.5, 0.25% glucose
*Pseudomonas aeruginosa* ATCC10145	1	0.59	18.75	pH 5.5, 1% TSB
*Escherichia coli* ATCC11775	1	>300	>300	pH 5.5, 1% TSB
*Candida albicans* ATCC 24433	2	1,18	9.38	pH 5.5, 0.25% glucose
*Candida albicans* ATCC 24433	1	18.8	150	pH 5.5, 1% TSB
*Burkholderia cepacia* ATCC 25416	1	>300	>300	pH 5.5, 0.25% glucose

*AA-sequence: GRHGSGLGHSSSHGQHGSGSGRSSSRGPY. CFU were examined in 10 mM NaP at indicated pH in the presence or absence of additives. All data shown are representatives. n: number of experiments.

We next tested the sensitivity of *P. aeruginosa* ATCC 10145, a skin commensal *Corynebacterium simulans* (*C. simulans*) DSM 44415, and *S. aureus* ATCC 6538 towards three selected CIDAMPs: FLG_528–554_, HRNR_232–294_ and HRNR_295–361_. Interestingly, FLG_528–554_ is highly active against *P. aeruginosa* ATCC 10145 but lacks activity against *C. simulans*. Both HRNR fragments, HRNR_232–294_ and HRNR_295–361_, show strong activity against *C. simulans*, as well as a high *P. aeruginosa-cidal* activity (Supplementary Table 10). All three peptides lack activity against *S. aureus* ATCC 6538 at concentrations <60 µM (data not shown).

In summary, these findings suggest that CIDAMPs originating from different locations within the HRNR protein or from other IDPs and IDPRs, are potent *P. aeruginosa*-cidal AMPs which may have in addition variable, more or less microbial species-selective antimicrobial activities.

### HRNR-fragments Exhibit Unique Protease-Sensitivity

**Figure 8:**
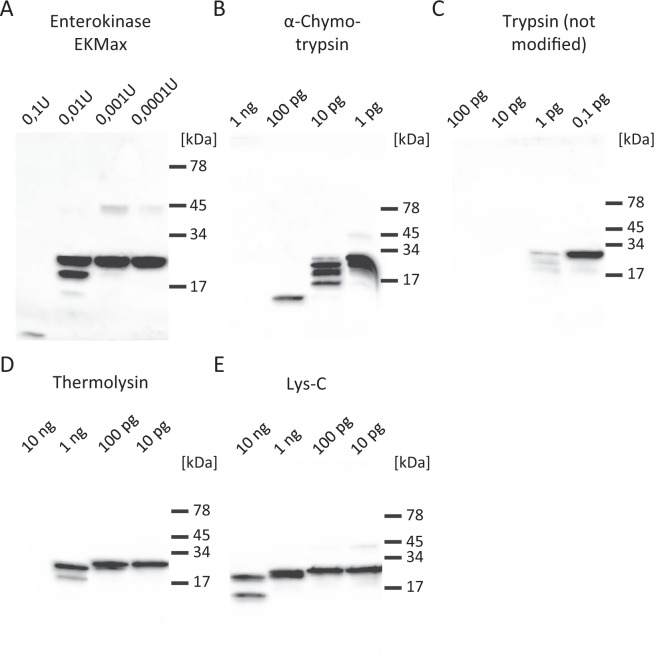
rHRNR_2576–2707_ is highly sensitive towards proteolysis. 10 µg rHRNR_2576–2707_ (for AA-sequence see Supplementary Table 2) each was incubated for 18 h at 37 °C with the respective protease in appropriate digestion buffers. Thereafter samples were subjected to a SDS-PAGE analysis and rHRNR_2576–2707_ and its fragments were detected by a HRNR-Western-Blot analysis using an in-house affinity-purified polyclonal α-HRNR_2591–2662_-antibody. We used **(a)** 0,1 U to 0,0001 U enterokinase EKMax, **(b)** 1 ng to 1 pg α-chymotrypsin, **(c)** 100 pg to 0,1 pg trypsin (not modified), **(d)** 10 ng to 10 pg thermolysin and **(e)** 10 ng to 10 pg Lys-C. Note the extraordinary sensitivity of µg-amounts rHRNR_2576–2707_ towards non-modified trypsin (**c**). All data shown are representatives (n = 3).

IDPs are inherently sensitive to proteolysis^35^. We found rHRNR_2576–2707_ to be highly sensitive towards trypsin, α-chymotrypsin, Lys-C, enterokinase EKMax and thermolysin (Fig. 8, Supplementary Fig. 12). Surprisingly, rHRNR_2576–2707_ is already digested by 1 pg unmodified trypsin (Fig. 8). Enterokinase EKMax, which we had initially been used to generate recombinant pET32-tag-free HRNR-fragments^12^, hydrolyses rHRNR_2576–2707_ - despite the absence of specific EKMax cleavage sites in HRNR. This observation would explain the technical difficulties to generate pET32-tag-free HRNR-fragments in our previous study^12^. Lys-C, a protease with a high specificity for peptide bonds C-terminal to lysine residues, is also able to cleave rHRNR_2576–2707_ - despite the absence of a Lys residue in this HRNR-protein fragment (Fig. 8e, Supplementary Fig. 12); it has to be noted that a much higher enzyme concentration than for tryptic digestion is necessary. In addition, some trypsin-like epithelial kallikreins^14^ such as KLK4, KLK5 and KLK14, can cleave rHRNR_2591–2684_ (Supplementary Fig. 13). Interestingly, however, Protein Arginine Deiminase 1 (PAD1)-deiminated rHRNR_2591–2684_ is still sensitive towards trypsin - although the specific cleavage sites (Arg) in this HRNR-fragment are now citrullinated (Supplementary Fig. 14). Further, the citrullinated HRNR is now sensitive towards KLK1, KLK7, KLK8 and shows a markedly increased sensitivity towards LysC - although predicted cleavage sites (Lys) are absent.

Thus, proteolytic enzymes of the host and microbes together with the environmental pH, will define the fragmentation patterns of stratum corneum-derived CIDAMP-sources, like HRNR, filaggrin-2 and filaggrin, and the local antimicrobial outcome.

### CIDAMPs are templates for designer anti-infectives

**Figure 9:**
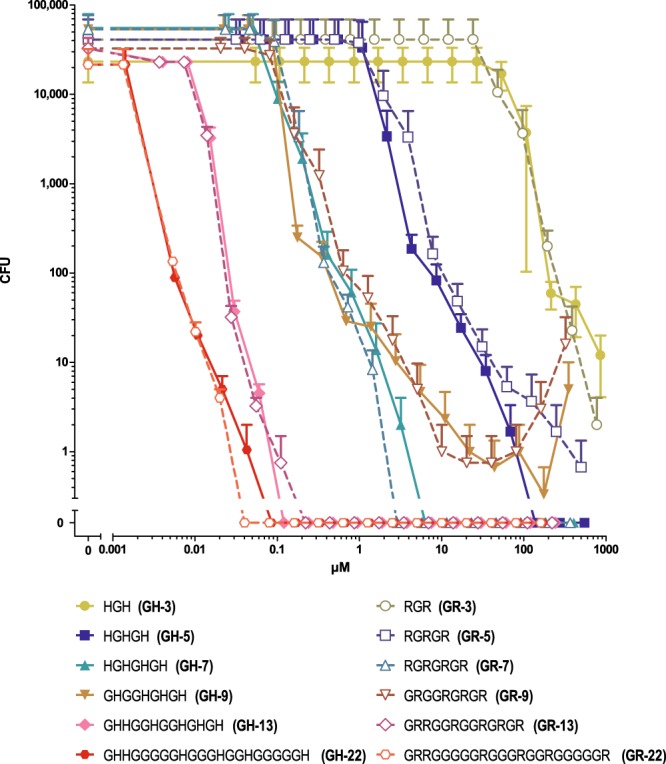
*P. aeruginosa*-cidal activity of Gly/His- and Gly/Arg-peptides depends on peptide chain length. *P. aeruginosa* ATCC 10145-cidal activity in 10 mM NaP, pH 5.5, containing 0.25% glucose, was examined for Gly/His-peptides and Gly/Arg-peptides of different peptide chain length in a CFU assay system. Error bars denote mean ± s.e.m. (n = 3).

Our data show that AA-substitutions and chemical modifications in HRNR-derived CIDAMPs alter their antimicrobial potency, efficacy and spectrum. This observation prompted us to test a library of chemically synthesized and structurally simple CIDAMPs, modified in their AA-composition, peptide chain length and/or by chemical derivatization, for antimicrobial activity. To strengthen our hypothesis that CIDAMP’s antimicrobial potency and efficacy depends on the peptide-chain length, we investigated peptides built solely by either Gly and Arg or Gly and His residues. Even tripeptides consisting of two His and one Gly or two Arg and one Gly exhibit antimicrobial activity. A continuous increase of the peptide chain length decreases the LD90 for both, Gly/His- and Gly/Arg-peptides, down to <10 nM (Fig. 9). CIDAMPs we had investigated thus far, contained mainly Arg and/or His and in a few cases also Lys as cationic AA. To elucidate whether the structure of basic AA defines the antimicrobial activity, 13-mer peptides containing Gly and the basic AA Arg, Lys, Orn or His were investigated. All four peptides studied reveal bactericidal activity against *P. aeruginosa* ATCC 10145 at nanomolar concentrations (Supplementary Fig. 16). This suggests that the presence of positively charged residues–independent from its structure - is a determining factor for *P. aeruginosa*-cidal activity. However, we observed marked differences of the LD100 values, with GR-13 and GH-13 being the most efficient bactericidal 13-mer CIDAMPs (Supplementary Fig. 16).

While HRNR-based CIDAMPs are potent *P. aeruginosa*-cidal AMPs, with much less activity towards *S. aureus*, hydrolysis-sensitive S-palmitoylated HRNR-peptides are potent *S. aureus*-cidal CIDAMPs (Fig. 7, Supplementary Table 9). This is also the case for N-terminal palmitoylated or myristoylated HRNR-peptides (Table 5, Supplementary Fig. 17). Surprisingly, however, some are also active at pH 7.3 and in the presence of 1% TSB as nutritional additive in the test medium.

**Table 5:** Palmitoylation and myristoylation of IDAMPs improves *S. aureus* ATCC 6538-bactericidal activity of HR1-18, HR1-11 and its Arg-mutants.

AA-sequence	Name	n	LD100 (µg/mL)	LD90 (µg/mL)	Condition
Pal-GRHGSGLGHSSSHGQHGSGSGR	Pal-HR 1-18, Pal-HRNR_2656–2677_	1	>300	>300	pH7.3, 1% TSB
Pal-GRHGSGLGHSSSHGQHGSGSGR	Pal-HR 1–18, Pal-HRNR_2656–2677_	2	37.5	1.18	pH5.5, 0.25% glucose
Myr-GRHGSGLGHSSSHGQHGSGSGR	Myr-HR 1-18, MyrHRNR_2656–2677_	1	75	9.38	pH5.5, 0.25% glucose
Myr-GRHGSGLGHSSSHGQHGSGSGR	Myr-HR 1-18, MyrHRNR_2656–2677_	1	75	18.75	pH7.3, 1% TSB
Myr-GRRGSGLGRSSSRGQRGSGSGR	Myr-HR 1-18-HR	1	75	18.75	pH7.3, 1% TSB
Pal-GRRGSGLGRSSSRGQRGSGSGR	Pal-HR 1-18-HR	2	4.7	0.3	pH5.5, 0.25% glucose
Pal-GRRGSGLGRSSSRGQRGSGSGR	Pal-HR 1-18-HR	1	9.38	1.18	pH7.3, 1% TSB
Pal-GRRGSGLGRSSSR	Pal-HR 1-18-3H3R-GR13	2	37.5	2.35	pH5.5, 0.25% glucose
Pal-HGSRSGQSSRGERHGSSSGSSSH	Pal-HR 1-11, HRNR_2606–2628_	1	>300	2.35	pH5.5, 0.25% glucose
Pal-RGSRSGQSSRGERRGSSSGSSSR	Pal-HR-1-11-3H3R	2	300	0.59	pH5.5, 0.25% glucose
Pal-RGSRSGQSSRGERR	Pal-HR-1-11-2H2R-14	1	18.75–75/>300*	0.59/>300*	pH5.5, 0.25% glucose

*Antimicrobial paradox^16^ (increased bacterial growth with increasing CIDAMP-concentrations, see (Fig. 3). Data shown are representatives. n: number of experiments.

In further experiments, ultra-short (4–13 AA), N-terminal palmitoylated CIDAMPs show *S. aureus*-cidal activity. Gly- and Arg-containing palmitoylated CIDAMPs with a peptide chain length of 4 to 13 AA show, by trend, an increase of the LD90 of *S. aureus*-cidal activity with decreasing peptide chain length, from Pal-GR13 (LD90: 47 nM) towards Pal-GR4 (LD90: 3.4 µM) (Table 6, Supplementary Fig. 17).

**Table 6:** Ultrashort palmitoylated Gly-rich CIDAMPs, containing structurally different cationic AA, are bactericidal for *S. aureus* ATCC 6538.

AA-sequence	Name	n	LD100 (µg/mL)	LD90 (µg/mL)	Condition
Pal-GHHGGHGGHGHGH	Pal-GH13	2	300	0.3	pH5.5, 0.25% glucose
Pal-GHHGGHGGHGHGH	Pal-GH13	1	>300	0.3	pH7.0, 0.25% glucose
Pal-GRRGGRGGRGRGR	Pal-GR13	1	4.7	0.3	pH5.5, 1% TSB
Pal-GRRGGRGGRGRGR	Pal-GR13	2	0.3	0.075	pH5.5, 0.25% glucose
Pal-GR_D_R_D_GGR_D_GGR_D_GR_D_GR_D_	(D)-Pal-GR13	2	75, LD99: 0.59	0.075	pH5.5, 0.25% glucose
Pal-GKKGGKGGKGKGK	Pal-K-GR13	2	>300	0.59	pH5.5, 0.25% glucose
Pal-GOOGGOGGOGOGO	Pal-Orn-GR13	2	18.75	0.15	pH5.5, 0.25% glucose
Pal-GRRGGRGGRGR	Pal-GR11	1	75,LD99: 1.18	0.3	pH7.3, 1% TSB
Pal-GRRGGRGGR	Pal-GR9	1	300, LD99: 4.7	1.18	pH5.5, 0.25% glucose
Pal-GR_D_R_D_GGR_D_GGR_D_	(D)-Pal-GR9	1	2.35	0.15	pH5.5, 0.25% glucose
Pal-GRRGGR	Pal-GR6	2	37.5, LD99: 4.7	2.35	pH5.5, 0.25% glucose
Pal-GRGR	Pal-GR4	2	9.375, LD99: 4.7	2.35	pH5.5, 0.25% glucose
Pal-GR_D_GR_D_	Pal-(D)-GR4	1	37.5	18.75	pH5.5, 0.25% glucose
Pal-GKGK	Pal-GK4	2	9.375	4.7	pH5.5, 0.25% glucose
Pal-GK_D_GK_D_	Pal-(D)-GK4	1	9.375	4.7	pH5.5, 0.25% glucose
Pal-GOGO	Pal-GO4	1	37. 5	9.38	pH5.5, 0.25% glucose
Pal-GO_D_GO_D_	Pal-(D)-GO4	1	18.75	9.38	pH5.5, 0.25% glucose
Pal-GlyDabGlyDab	Pal-G-Dab4	2	18.75	4.7	pH5.5, 0.25% glucose
Pal-GlyDapGlyDap	Pal-G-Dap4	2	4.7	2.35	pH5.5, 0.25% glucose

LD99: Concentration of peptide, which kills 99.0% of inoculum, determined in the CFU-assay system. Dab: L-2,4-Diaminobutyric acid, Dap: L-2,3-Diaminopropionic acid. Data shown are representatives. N: number of experiments.

Arg- or Lys-containing CIDAMPs might be prone to digestion by tryptic proteases at neutral pH. To investigate whether an exchange of natural L-AAs against their D-enantiomers affects staphylocidal activity, we investigated a Pal-GR-13 variant, in which all L-Arg have been substituted by D-Arg. We found similar *S. aureus*-cidal activity when compared to the L-Arg peptides, but altered efficacies (Table 6, Supplementary Fig. 17). In addition, these lipidated CIDAMPs retain their strong *P. aeruginosa*-cidal activity (Supplementary Table 11).

As stated above, positively charged AA residues - independent of the peptide structure–were identified as important features for *P. aeruginosa*-cidal activity of CIDAMPs (Supplementary Fig. 16). To investigate whether this is also true for *S. aureus*-cidal activity in palmitoylated CIDAMPs, analogues peptides containing either the natural AA His, Lys, Arg or the nonproteinogenic AA L-Orn, L-(2,4)-Di-amino butyric acid (Dab) or L-(2,3)-Di-amino propionic acid (Dap) substituting Arg-residues were studied. Whereas the non-palmitoylated peptides mostly lack *S. aureus* ATCC 6538-cidal activity (data not shown), the palmitoylated CIDAMPs show activity, with Pal-GR-13 being the most potent antibacterial lipopeptide. Among the ultrashort CIDAMPs, Pal-G-Dap4 was identified as the most efficient peptide (Table 6, Supplementary Fig. 17). Interestingly, the lipopeptide Pal-GH13 reveals a striking pH-dependency of *S. aureus* ATCC 6538-cidal activity, causing at pH > 7 an “antibiotic paradox”^36^, which is characterized by an increase of CFU with increasing lipopeptide concentrations. This finally results in a complete loss of bactericidal activity at pH 7.5 at concentrations >8 µM and <0.1 µM (Supplementary Fig. 18).

**Figure 10:**
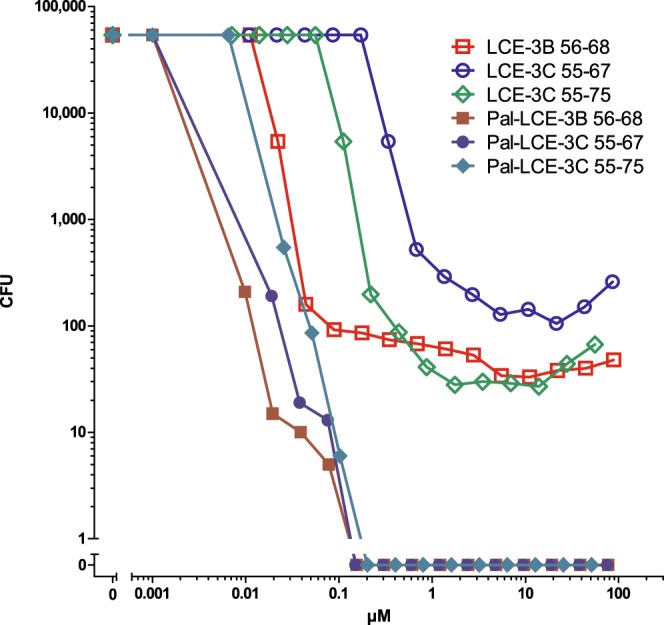
Late Cornified Envelope Protein (LCE)-fragments and its palmitoylated derivatives are potent *S. aureus*-cidal antimicrobials. *S. aureus* ATCC 6538-cidal activity was examined in a CFU assay system in 10 mM NaP, pH 5.5, containing 0.25% glucose. For AA-sequences see Supplementary Table 12. Representatives are shown (n = 2).

Whereas peptide fragments from “Late Cornified Envelope Proteins (LCEs)”, LCE-2B_74–86_, LCE-3B_56–68_, LCE-3C_55–67_ and LCE-3C_55–75_ are potent *S. aureus*-cidal CIDAMPs (Fig. 6, Supplementary Table 8), N-palmitoylation improves its staphylocidal activity further, now eradicating *S. aureus* at nanomolar concentration with Pal-LCE-3B_56–68_ as the most potent and efficient CIDAMP in this study (Fig. 10, Supplementary Table 8).

In summary, lipidation of CIDAMPs improves their antimicrobial potency and efficacy. Palmitoylated Gly- and Arg-rich peptides and, in particular, palmitoylated LCE-3B- and LCE-3C-peptides, are very potent and efficient staphylocidal CIDAMPs when tested in the absence of nutritional additives. In addition, palmitoylated Gly- and Arg-rich peptides are still potent *P. aeruginosa*-cidal antimicrobials.

### Lipidated CIDAMPs are broad-spectrum antimicrobials

A few distinct HRNR-based CIDAMPs were tested for possible broad-spectrum antimicrobial activity and were found to be bactericidal for a narrow spectrum of diverse bacteria (Tables 3 and 4, Supplementary Table 10). Based upon our observation that many palmitoylated CIDAMPs are active against both, *S. aureus-* and *P. aeruginosa*, we surmised that these lipopeptides could be broad-spectrum antimicrobials. To test this hypothesis, we selected four different N-palmitoylated CIDAMPs, Pal-GR13, Pal-LCE-3B_56–68_, Pal-HR1-18 and Pal-HR1-18-HR for sensitivity-testing of a panel of Gram-positive, Gram-negative anaerobic and aerobic pathogens and commensals, respectively. Pal-GR13 was found to be a very potent and efficient CIDAMP with some interesting features: *S. aureus* ATCC 6538 is highly sensitive at the physiological skin pH 5.5, but less at pH 7.3; unexpectedly, at pH 5.5, the commensal *Staphylococcus epidermidis* ATCC 14990 is rather insensitive towards this lipopeptide, but at pH 7.3, sensitivity is similar as seen for *S. aureus* (Table 7, Supplementary Fig. 19). Intriguingly, *Lactobacillus crispatus*, another commensal of the normal vaginal microflora^37^ as well as of the deep stratum corneum layers of the skin^38^ and the commensal *Peptostreptococcus magnus* (*Finegoldia magna*) show a low sensitivity at the given test conditions (Table 7, Supplementary Fig. 19). Further, *E. coli* ATCC 11775, *P. aeruginosa* ATCC 10145, *Klebsiella pneumoniae* ATCC 13883 and *Propionibacterium acnes* ATCC 6919 are highly sensitive towards this lipopeptide (Table 7, Supplementary Fig. 19). Pal-LCE-3B_56–68_ is the most active CIDAMP against *S. aureus* ATCC 6538 at pH 5.5 (Table 8, Supplementary Fig. 20). Again, *Staphylococcus epidermidis* ATCC 14990 is far less sensitive towards this lipopeptide at pH 5.5. Compared with Pal-GR13, several strains show similar susceptibilities, as seen for the tested strains of *E. coli*, *Streptococcus pyogenes*, and *Salmonella typhimurium* at pH 7.3. At pH 5.5, however, the latter is highly sensitive to this lipopeptide (Supplementary Fig. 20, Table 8). In addition, sensitivity of *Streptococcus pneumoniae* towards this lipopeptide is higher than seen towards Pal-GR13. On the other hand, Pal-LCE-3B_56–68_ efficacy (LD100) to eradicate *Klebsiella pneumoniae* ATCC 13883 is lower than that of Pal-GR13 at pH 5.5 (Table 7). Pal-HR1-18 and Pal-HR1-18HR reveal a similar activity pattern and are potent and efficient bactericidal AMPs for the examined strains of *P. aeruginosa*, *Streptococcus pyogenes*, *Klebsiella pneumoniae* and *Propionibacterium acnes*, but not *S. aureus* (Supplementary Tables 13 and 14).

**Table 7:** Antimicrobial activity spectrum of Pal-GR13^*^.

Microorganism	n	LD100 (µg/mL)	LD90 (µg/mL)	Conditions
*Burkholderia cepacia* ATCC 25416	2	18.75	18.75	pH 7.3, 1% TSB
*Burkholderia cepacia* ATCC 25416	3	9.375	4.7	pH 7.3, 0.25% glucose
*Burkholderia cepacia* ATCC 25416	2	>300	>300	pH 5.5, 0.25% glucose
*Escherichia coli* ATCC 11775	1	2.35	1.18	pH 7.3, 1% TSB
*Escherichia coli* ATCC 11775	2	0.59	0.15	pH 5.5, 0.25% glucose
*Klebsiella pneumoniae* ATCC 13883	1	1.18	1.18	pH 7.3, 1% TSB
*Klebsiella pneumoniae* ATCC 13883	2	0.3	0.15	pH 5.5, 0.25% glucose
*Moraxella osloensis* RV A2/2001	1	4.7	0.59	pH 7.3, 1% TSB
*Prevotella oralis* ATCC 33321	1	18.75	9.38	pH 7.3, 1% TSB
*Pseudomonas aeruginosa* ATCC 10145	2	1.18	0.3	pH 7.3, 1% TSB
*Pseudomonas aeruginosa* ATCC10145	2	0.3	0.075	pH 5.5, 0.25% glucose
*Salmonella typhimurium* ATCC 13311	2	2.35	2.35	pH 7.3, 1% TSB
*Clostridium perfringens* ATCC 13124	1	37.5	18.75	pH 7.3, 1% TSB
*Clostridium perfringens* ATCC 13124	1	18.75	4.7	pH 7.3, 0.25% glucose
*Clostridium perfringens* ATCC 13124	1	75	37.5	pH 5.5, 0.25% glucose
*Corynebacterium simulans* DSM 44415	1	2.35	0.59	pH 7.3, 1% TSB
*Lactobacillus crispatus* DSM 20584	2	**75**	**9.38**	pH 7.3, 1% TSB
*Peptostreptococcus magnus* ATCC 15794**	2	**37.5**	**4.7**	pH 7.3, 1% TSB
*Propionibacterium acnes* ATCC 6919	1	4.7	1.18	pH 7.3, 1% TSB
*Propionibacterium acnes* ATCC 6919	1	2.35	0.59	pH 7.3, 0.25% glucose
*Propionibacterium acnes* ATCC 6919	2	0.59	0.3	pH 5.5, 0.25% glucose
*Staphylococcus aureus* ATCC 6538	2	4.7	0.3	pH 7.3, 1% TSB
*Staphylococcus aureus* ATCC 6538	2	>300	0.15	pH 5.5, 0.25% glucose
*Staphylococcus epidermidis* ATCC 14990	2	300	9.38	pH 5.5, 0.25% glucose
*Staphylococcus epidermidis* ATCC 14990	2	4.7	0.59	pH 7.3, 1% TSB
*Staphylococcus hominis* ATCC 27844	1	4.7	1.18	pH 7.3, 1% TSB
*Streptococcus pneumoniae* ATCC 33400	1	4.7	2.35	pH 7.3, 1% TSB
*Streptococcus pyogenes* ATCC 12344	1	0.3	0.15	pH 7.3, 1% TSB

*AA-sequence: Pal-GRRGGRGGRGRGR. **Now *Finegoldia magna*. Sensitivity of bacteria towards Pal-GR13 was tested in a CFU assay system in 10 mM NaP containing 0.25% glucose and 1% TSB, respectively, and, depending on the microbe, at pH 5.5, 6.0, 6.5 or pH 7.3. Representatives are shown. N: number of experiments.

**Table 8:** Antimicrobial activity spectrum of Pal-LCE-3B_56–68_*.

Microorganism	n	LD100 (µg/mL)	LD90 (µg/mL)	Conditions
*Burkholderia cepacia* ATCC 25416	3	9.375	4.7	pH 7.3
*Burkholderia cepacia* ATCC 25416	3	>300	>300	pH 5.5
*Escherichia coli* ATCC11775	1	1.18	0.59	pH 7.3
*Escherichia coli* ATCC11775	1	>300/37.5**	0.3	pH 5.5
*Klebsiella pneumoniae* ATCC 13883	2	4.7	2.35	pH 7.3
*Klebsiella pneumoniae* ATCC 13883	2	9.375	0.15	pH 5.5
*Pseudomonas aeruginosa* ATCC10145	2	0.3	0.075	pH 5.5
*Salmonella typhimurium* ATCC 13311	2	2.35	1.18	pH 7.3
*Salmonella typhimurium* ATCC 13311	2	0.3	0.038	pH 5.5
*Staphylococcus aureus* ATCC 6538	2	0.15	<0.019	pH 5.5
*Staphylococcus epidermidis* ATCC 14990	2	*0.3*	*0.3*	pH 5.5
*Streptococcus pneumoniae* ATCC 33400	1	2.35	0.59	pH 7.3
*Streptococcus pyogenes* ATCC 12344	1	0.59	0.15	pH 6.0
*Streptococcus pyogenes* ATCC 12344	1	0.3	0.15	pH 7.3

*AA-sequence: Pal-SHHRCCRSHRCRR. **Antimicrobial paradox (increased bacterial growth with increasing IDAMP-concentrations). Sensitivity of bacteria towards Pal-LCE-3B_56–68_ was tested in a CFU assay system in 10 mM NaP containing 0.25% glucose and, depending on the microbe, at a pH of 5.5, 6.0 or pH 7.3. Representatives are shown. n: number of experiments.

In summary, this exploratory study suggests that it should be possible to generate potent and microbial target-selective CIDAMPs by modifying their AA-composition together with lipidation.

### CIDAMPs Kill Drug-Resistant Microbes

To investigate whether at least some of the newly designed CIDAMPs may also be active towards antibiotic drug-resistant microbes, a panel of 69 structurally different CIDAMPs revealing potent *P. aeruginosa*-cidal activity at our standard acidic test conditions, were independently screened by the not-for-profit initiative CO-ADD (Community for Open Antimicrobial Drug Discovery)^37^ (http://www.co-add.org/) against a key panel of drug-resistant and control bacterial strains (*E. coli*, a multi-drug resistant *Klebsiella pneumoniae* (MDR)*, Acinetobacter baumannii, P. aeruginosa*, a methicillin-resistant *S. aureus* strain (MRSA)and two yeasts (*C. albicans* and *Cryptococcus neoformans*) for minimum inhibitory concentrations (MICs). Tests were performed at CLSI-recommended conditions (http://clsi.org/standards/micro/), which are commonly used for antibiotic discovery.

While all non-palmitoylated HRNR- and FLG-2-based Gly-rich CIDAMPs are inactive at CLSI conditions against all bacterial and yeast strains tested - as expected from our data on HRNR-derived CIDAMPs (Supplementary Table 2) - some of the LCE-peptides are surprisingly active against one or both yeast strains (Table 9, Supplementary Tables 15 and 16). Other peptides show a high variety of different antimicrobial activity patterns with a marked diversity of antimicrobial potencies. In addition, some CIDAMPs, like Pal-GR13, Pal-HR1-18HR and Pal-GR11, are broad-spectrum peptide antibiotics. Both ultrashort lipopeptides Pal-GR4 and Pal-GDap4, show a restricted antimicrobial spectrum against the two yeasts and the MRSA strain (Table 9, Supplementary Tables 15 and 16). Among the hits of the 69 investigated CIDAMPs, we identified the lipopeptides Pal-HR1-18HR, Pal-GR13 and (D)-Pal-GR13 as the most potent antimicrobials against *E. coli* ATCC 25922; Pal-GR13 against *Klebsiella pneumoniae* ATCC 700603(MDR); Pal-HR1-18HR, Pal-HR-1-11-2H2R-14, Pal-GR13 and (D)-Pal-GR13 against *Acinetobacter baumannii* ATCC 19606; Pal-GR13 against *P. aeruginosa* ATCC27853; Pal-GR13, (D)-Pal-GR13, Pal-GR11, C-Pal-GR13 and 8C-Pal-GR13 against *S. aureus* ATCC 43300 (MRSA) and LCE-3B_56−68_ and C-Pal-GR13 against *C. albicans* ATCC 90028.

**Table 9:** Antimicrobial properties of selected CIDAMPs in Mueller-Hinton assay-medium.

Name	Amino Acid Sequence	*Acinetobacter baumannii ATCC 19606*	*Candida albicans ATCC 90028*	*Cryptococcus neoformans ATCC 208821*	*E. coli* ATCC 25922	*Klebsiella pneumonia ATCC 700603*	*Pseudomonas aeruginosa ATCC 27853*	*Staphylococcus aureus ATCC 43300*
FLG-2 (2082–2100)	HAHSGHGQSTQRGSRTAGR	>32	>32	>32	>32	>32	>32	>32
HR1-18, HRNR_2656–2677_	GRHGSGLGHSSSHGQHGSGSGR	>32	>32	>32	>32	>32	>32	>32
Pal-HR1-18	Pal-GRHGSGLGHSSSHGQHGSGSGR	32	>32	>32	>32	>32	>32	>32
HR1-18,4H4R	GRRGSGLGRSSSRGQRGSGSGR	>32	>32	>32	>32	>32	>32	>32
Pal-HR1-18HR	Pal-GRRGSGLGRSSSRGQRGSGSGR	1	>32	1	4	>32	16	8
Pal-HR1-18-3H3R-GR13	Pal-GRRGSGLGRSSSR	16	>32	16	>32	>32	>32	16
Pal-HR1-11	Pal-HGSRSGQSSRGERHGSSSGSSSH	>32	>32	>32	>32	>32	>32	32
Pal-HR1-11,2H2R-14	Pal-RGSRSGQSSRGERR	4	>32	8	8	>32	>32	8
Pal-HR1-11,3H3R	Pal-RGSRSGQSSRGERRGSSSGSSSR	8	32	32	32	>32	>32	32
Pal-GDab4	Pal-GlyDabGlyDab	32	>32	16	>32	>32	>32	32
Pal-GDap4	Pal-GlyDapGlyDap	>32	8	1	>32	>32	>32	8
Pal-GR4	Pal-GRGR	>32	8	8	>32	>32	>32	8
Pal-GR6	Pal-GRRGGR	>32	>32	16	>32	>32	>32	16
Pal-GR9	Pal-GRRGGRGGR	>32	>32	>32	>32	>32	>32	8
Pal-GR11	Pal-GRRGGRGGRGR	8	4	4	8	>32	16	4
GR-13	GRRGGRGGRGRGR	>32	>32	>32	>32	>32	>32	>32
Pal-GR13	Pal-GRRGGRGGRGRGR	2	4	0.25	4	≥16	8	0.25
D-Pal-GR13	Pal-GRDRDGGRDGGRDGRDGRD	8	8	2	8	≥32	16	2
LCE3B (56-68)	SHHRCCRSHRCRR	>32	>32	8	>32	>32	>32	32
Pal-LCE3B (56–68)	Pal-SHHRCCRSHRCRR	>32	1	0.25	>32	>32	>32	16
LCE3C (55–67)	SHHRHFRSHQCRR	>32	>32	8	>32	>32	>32	32
Pal-LCE3C (55–67)	Pal-SHHRHFRSHQCRR	>32	16	1	>32	>32	>32	32

Minimal inhibitory concentration (MIC in µg/mL) is shown. R_D_: AA in its D form, Dab = L-2, 4-Diaminobutyric acid, Dap = L-2, 3-Diaminopropionic acid; Pal: N-palmitoyl. MIC values for bacteria were determined according to the CLSI (Clinical and Laboratory Standards Institute) guidelines in Mueller–Hinton (MH) broth for bacteria and in Yeast Nitrogen Base (YNB) broth for fungi. For raw data see Supplementary Table 15.

N-terminal palmitoylated GR13 (Pal-GR13) is the most potent broad-spectrum antimicrobial under CLSI test conditions. S-palmitoylated Cys-derivatives of GR13, which are slightly less potent in killing the MRSA-strain, show a similar antimicrobial potency–irrespective whether the palmitoylated Cys-residue is located at the C-terminus (C-Pal-GR13) or within the GR13-peptide chain (8C-Pal-GR13) (Table 9, Supplementary Tables 15 and 16). In addition, the D-Arg-enantiomer of Pal-GR13, which would be expected to be a more stable peptide antibiotic at conditions with high tryptic enzyme loads^38^ and a rather neutral environmental pH, show a similar broad antimicrobial spectrum as the L-Arg-form (Table 9, Supplementary Tables 15 and 16).

To investigate possible cytotoxic properties, 69 mostly lipidated CIDAMPs were tested on the human embryonic kidney cell line HEK293 at concentrations ≤32 µg/mL (Supplementary Tables 15 and 16) and show low or no cytotoxicity. Thus CIDAMPs resemble structurally related proline-rich AMPs, which are in general not toxic to mammalian cells^39^.

### Unique Ultrastructural Features of CIDAMP-treated Microorganisms

**Figure 11:**
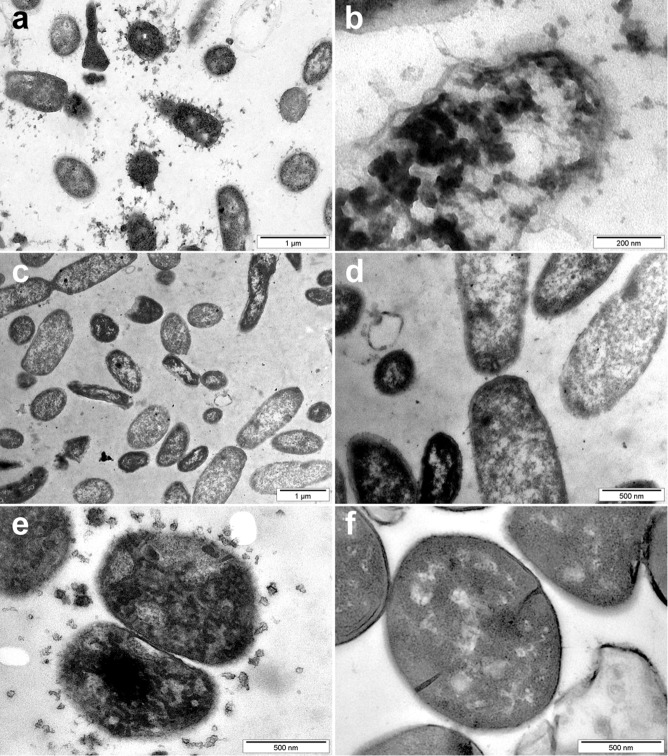
The membrane and ribosomes are targets of Pal-GR13-treated microbes. 10^7^ *P. aeruginosa* ATCC 10145 (**a**,**b**) or *S. aureus* ATCC 6538 (**e**), suspended in 150 µl 10 mM NaP, pH 7.3, containing 1% TSB, were treated with Pal-GR13 (Pal-GRRGGRGGRGRGR, 375 µg/ml) for 60 min at ambient temperature and then analyzed by transmission electron microscopy (TEM). Buffer-treated *P. aeruginosa* ATCC 10145 (**c**,**d**) and *S. aureus* ATCC 6538 (**f**) served as controls. Note condensation of electron-dense cytoplasmic material and liberation of mesosome-like vesicles in Pal-GR13-treated bacteria (**a,b,e**). Images are representative of two independent experiments, sampling on average 10 images per condition in each experiment.

To gain insight in the mode of action of antimicrobials, transmission electron microscopy (TEM)-studies can be informative. HRNR-derived CIDAMPs are ribosome-targeting antimicrobials, which lead to unique ultrastructural features with electron-dense cytoplasmic aggregates as a general characteristic. However, they show no signs of immediate membrane alteration^40^– that are typical features of pore-forming amphipathic antimicrobial peptides such as cathelicidin LL37^41^ and also defensins^42^. Similarly, in TEM of LCE3B_56−68_-challenged *S. aureus* ATCC 6538 these electron-dense cytoplasmic aggregates are also observable (Supplementary Fig. 21). Interestingly, in Pal-GR13-challenged *P. aeruginosa* ATCC 10145 together with these electron-dense cytoplasmic aggregates, small membrane vesicles are found (Fig. 11a,b). These are also observed in Pal-GR13-treated *S. aureus* ATCC 6538 (Fig. 11e) and other Pal-GR13-treated bacterial species, including *E. coli* ATCC 11775*, Burkholderia cepacia ATCC 25416, Clostridium perfringens* ATCC 13124, *Propionibacterium acnes* ATCC 6919, *Prevotella oralis* ATCC 33321 and *Staphylococcus epidermidis* ATCC 14990 (Supplementary Fig. 22a–m). Accordingly, the very fact that these mesosome-like structures are seen in most palmitoylated CIDAMP-treated cells is indicative of cytoplasmic-membrane alteration and (possibly) uncoupling of the synthesis and turnover of cell wall polymers^43^.

*C. albicans* ATCC 24433, treated with Pal-GR-13, reveals characteristic ultrastructural patterns with the release of electron-dense membrane vesicles (Supplementary Fig. 23). These are very similar to morphological features seen upon treatment of *C. albicans* with the HRNR fragment HRNR_2591–2684_^40^. They are a hallmark ultrastructural signs of apoptosis in pathogenic fungi^44,45^. This supports the hypothesis that Pal-GR-13 might kill *C. albicans* using similar mechanisms as AMPs like lactoferrin^46^, human ß-defensins^47^, plant defensins^48^ and probably HRNR_2591–2684_ ^40^, i.e. by apoptosis-like cell death.

## Discussion

Our study provides strong evidence towards the existence of an antimicrobial defense system on the outermost parts of body surfaces such as human skin and other barrier organs. This is based on the versatile generation of a multitude of cationic intrinsically disordered antimicrobial peptides, which we propose to term “CIDAMPs”. These AMPs contain a high percentage of disorder-promoting AA and a low percentage of order-promoting AA which build linear intrinsically disordered peptides of varying lengths carrying a positive net charge.

CIDAMPs are mostly present as linked series in repeat domains of positively charged epidermal differentiation complex proteins and protein-regions, which are rich in disorder-promoting polar amino acids (i.e. Gly/Ser/Thr/Gln/His/Pro) and low in order-promoting, hydrophobic AA (i.e. Leu/Ile/Val/Asp/Tyr/Phe/Trp). These proteins are both strategically and optimally located within the stratum corneum^12,49^. They are a potential source for huge numbers (e. g. in HRNR approximately 4 million different, putatively microbicidal polypeptide fragments with chain lengths >10 AA) of slightly different CIDAMPs. Interestingly, until now only 122 HRNR peptides have been identified in stratum corneum (Fig. 2, Supplementary Fig. 1). This suggests a coordinated cleavage of HRNR by host and/or microbial proteases within the horny layer, generating protease-cleavage site- and AA-composition-dependent, target-selective CIDAMPs.

HRNR seems to be the most abundant source of CIDAMPs in healthy skin. Furthermore, quantitative proteome analyses have identified HRNR as a highly abundant protein in humans^50^. Epithelial cell types in barrier organs (e.g. kidney, skin with hair follicles, lung, vulva, rectum, colon, urinary bladder, uterine cervix, and placenta) exhibit highest HRNR-abundances (top 5–25% of identified proteins). Further, immune privileged organs and organs in which vital structures need to be protected from the potentially damaging effects of an inflammatory immune response (e.g. brain, eye, central nervous system, female gonads, placenta, teeth, and heart) reveal a similar HRNR abundance^50–53^. Notably, HRNR is an abundant protein in earwax (http://pax-db.org/protein/1854883/HRNR) and in extracellular vesicles, where it was found in nasal and bronchial secretions^54^, in platelet-derived microparticles, plasma exosomes^55^, urine exosomes and podocyte-enriched exosome fractions of normal human urine, in exhaled breath condensate^56^ as well as in extracellular vesicles released by many cell lines (http://microvesicles.org/gene_summary?gene_id=388697). This finding is nourishing the hypothesis that these particles could have a role in innate defense, trapping microbes and subsequently killing them by HRNR fragments.

We therefore propose that the highly abundant protein HRNR is an important precursor for a collection of versatile local disinfectants, acting at the outermost surface of barrier organs, helping to keep healthy skin and mucosal surfaces free of infection.

Although only a few fragments of HRNR, FLG-2, FLG, RPT or LCEs have been thoroughly characterized (antimicrobial potency, efficacy, microbial target spectrum, and optimal activity conditions), our exploratory site-directed mutation studies reveal evidence that distinct HRNR-fragments, and fragments of other IDPRs, have microbe-specific antimicrobial activity under certain conditions. Here, different N- and C-termini, a varying AA-composition, the extent of post-translational modification, and the peptide chain length are the major factors influencing the antimicrobial output. Interestingly, the highest potency and efficacy of HRNR-fragments towards the opportunistic pathogen *P. aeruginosa* was observed only under acidic pH and at no to low levels of soluble nutrients. Importantly, these conditions are found at the nasal mucosal surface^57^ and within healthy skin stratum corneum^58^. The loss of antimicrobial activity of several CIDAMPs at neutral pH can be attributed towards their high histidine content, which is cationic only at acidic conditions (pK_s_: 6.0). This phenomenon is known from several other, His-rich AMPs^17^ which are active only at acidic pH.

Derivatization and AA-substitutions can highly affect antimicrobial properties of HRNR fragments. S-palmitoylation introduces, or markedly increases, the activity towards *S. aureus* (Fig. 7). Moreover, CIDAMPs content of positively charged AA influences its antimicrobial potency and efficacy. HRNR and other CIDAMP sources seem to require at least four cationic residues (at environmental pH) for potent (nanomolar) bactericidal activity (Supplementary Table 6). Further, replacement of a single AA can alter CIDAMPs activity. This indicates that an AA-exchange, e.g. by mutation in cationic IDPRs, could have consequences for the antimicrobial spectrum of CIDAMPs. In this context, it is intriguing to note that 7769 variations have currently been identified in the *HRNR* gene (http://www.ensembl.org/Homo_sapiens/Transcript/ProteinSummary?g=ENSG00000197915). It would be interesting to study whether at least some of these variations alter protease cleavage sites, potentially generating CIDAMPs with different N- and/or C-termini, which possibly lead to altered antimicrobial properties. Furthermore, this could potentially cause changes in the microbiome at HRNR-rich locations, such as the stratum corneum.

An increase of the environmental pH would lead to a decreased net positive charge in HRNR and other His-rich CIDAMPs and would thus reduce their antimicrobial potency and efficacy (Supplementary Fig. 6). As a consequence, the composition of the local microbiome would be altered. More critical, however, would be an effect of increased pH on the peptide chain length of CIDAMPs present at the skin surface: picogram amounts of trypsin (Fig. 8) and possibly skin-derived tryptic enzymes such as several kallikreins^14^, which have a pH optimum at neutral to alkaline pH, would cleave Arg- and Lys-containing CIDAMPs. This would lead to very short CIDAMPs, containing in the most part only a single Arg or Lys at the C-terminus. Due to a very low positive net charge at neutral pH, the antimicrobial activity would be, if retained at all, reduced (Supplementary Table 6). Such effects could be highly relevant in atopic dermatitis (AD), especially as *HRNR* represents an AD susceptibility gene^59^. In AD, a reduced HRNR production has been observed within the skin^12,60^. Further, even in non-lesional areas, the skin of AD patients has a more neutral pH^61,62^, tissue kallikrein-derived tryptic activity is elevated^63^, and an altered AD skin microbiome, with an overgrowth and often skin infection by *S. aureus*, is observed^64^. It would be intriguing to study, whether AD patient’s stratum corneum contains kallikrein-degraded (and likely antimicrobially inactive) HRNR-fragments. If so, this would help to explain why *S. aureus*, which grows well at neutral but not at acidic pH^65,66^, abnormally inhabits AD skin. Thus, HRNR could play a critical role in AD pathophysiology and might contribute to the altered skin microbiome in this disease.

Our findings suggest that any intrinsically disordered protein region with a positive net charge, and dependent on its AA-composition and peptide chain length, represents a potential antimicrobial. This is the case for antimicrobial peptides like epsilon-poly-L-lysine, a natural antimicrobial cationic peptide^67^, as well as poly-L-arginine^68^, which both are generally regarded as safe food preservatives.

Among already known antimicrobial proteins, some of them are, at least in part, also CIDAMPs due to their high content of disorder promoting AA and a positive net charge. This is, for example, the case for antimicrobially active peptide fragments of the intrinsically disordered protein casein^69^, histatin-5, histones, proline-rich and glycine-rich insect AMPs^70^, as well as for Gly/Ser-rich C-terminal antimicrobial regions of salmonid cathelicidins. Further, this is the case for chicken cathelicidins (hybrid proteins that contain ordered domains and functional intrinsically disordered regions), as well as Gly-His-rich proteins in plants. A protein Blast search (https://blast.ncbi.nlm.nih.gov/Blast.cgi) for hypothetical Gly/Arg/His-rich peptide sequences [e.g., both highly potent *P. aeruginosa*-cidal CIDAMPs **HR1-18,G + H** (GHHGGGGGHGGGHGGHGGGGGH) and **HR1-18,G + R** (GRRGGGGGRGGGRGGRGGGGGR) (Supplementary Table 3)], reveals similar cationic IDPRs e.g. in the horseshoe crab, as well as in several insects, plants, birds and even bacteria. With other AA-compositions, a huge number of His- or Arg-rich potential IDPR-containing proteins, widespread in organisms from all kingdoms of life, are expected to be identified as predictive cationic IDPRs. Among them are putative HRNR orthologs in many mammals, in particular primates, as well as “hornerin-like proteins” in birds, insects and plants. Gly/Arg-rich proteins are also present in the wild strawberry, in the sweet water polyp, and in the glycine-rich cell wall structural protein of the tomato.

It is tempting to speculate that many of these predicted cationic IDPRs would represent presumptive CIDAMP sources and thus could be important as innate defense effectors of the respective organisms. We therefore propose that CIDAMPs are generated in an adaptive, environmental condition-dependent manner, where host proteases and microbial proteases determine their N- and C-termini as well as the peptide length. This potentially generates microbial target-selective CIDAMPs that would be active only at specific environmental conditions. CIDAMPs generated from crosslinked and insoluble epithelial proteins would represent effector molecules of an adaptive innate defense system, having the potential for the generation of pathogen-selective designer peptide antibiotics and disinfectants. Therefore, as proof-of-principle, we also explored the antimicrobial potential of chemically synthesized cationic peptides designed from HRNR- and LCE-derived CIDAMPs. In general, our findings suggest that it is possible to generate CIDAMP-based anti-infectives with potent microbicidal activity towards a high number of distinct pathogens and concomitant low activity towards “good” commensals and low - if any - cytotoxic activity towards a human cell line.

The simple AA-composition, linear peptide structure, and chemical modification make CIDAMPs very versatile microbicidal peptides. The intrinsically disordered structure would give CIDAMP designers a great degree of flexibility compared to AMPs, whose activity is based on secondary structure elements. Virtually any imaginable AA-sequence combination or composition of disorder promoting AA together with cationic AA and a low percentage of order-promoting AA could be envisioned and further subjected to experimental validation.

N-palmitoylated CIDAMPs revealed a striking increase in antimicrobial potency and efficacy when tested in nutrient-free medium (Tables 5 and 6; Supplementary Figs 15 and 17). Ultrastructural analyses of palmitoylated CIDAMP-treated bacteria suggest membrane effects, similar to those reported for palmitoylated short cationic peptides^33^, and unlike in bacteria treated with HRNR-based CIDAMPs^40^. One may speculate that palmitoylated CIDAMPs, depending on the environmental conditions, are utilizing both an energy-dependent peptide-channel^40^, and a lipid-dependent pathway that involves permeation and disintegration of membranes, similar to that of many long antimicrobial peptides. Thus, lipidation of CIDAMPs would increase the antimicrobial potency and efficacy of candidate CIDAMPs and, as seen in selected HRNR-derived CIDAMPs (Tables 3 and 4), their antimicrobial spectrum (Table 7). Improvement of antimicrobial potency, efficacy, and possibly also changes in the antimicrobial spectrum of CIDAMPs, could be achieved by introducing metal-binding motifs^27,29,71–73^. This might help to explain the preferential and potent *S. aureus*-targeting antimicrobial activity of the investigated LCE-derived CIDAMPs (Figs 6 and 10; Table 8; Supplementary Fig. 20). A combination of all these parameters to modify the simple chemical structure of CIDAMPs will generate a plethora of distinct peptides with different antimicrobial properties; as shown in a few examples within our exploratory study.

Our findings suggest that selected CIDAMPs might have a potential for developing novel *P. aeruginosa*-targeting peptide antibiotics. *P. aeruginosa* is currently among the leading causes of severe nosocomial infections, particularly affecting critically ill and immunocompromised patients^74^. *P. aeruginosa* infections are becoming more difficult to treat because this bacterium is naturally resistant or even less sensitive to antibiotics than most other Gram-negative bacteria^75^. Additionally, the number of multidrug- and pan-drug-resistant strains of *P. aeruginosa* is increasing worldwide. A very high number of structurally different Gly-rich CIDAMPs (Fig. 9, Supplementary Tables 3–7) are non-toxic, potent and efficient microbicidal AMPs, eradicating *P. aeruginosa* ATCC 10145 under environmental conditions present on skin and mucosal surfaces. Some CIDAMPs are active also under CLSI-test conditions suggesting a potential for systemic application. Thus, most of the non-lipidated *P. aeruginosa*-targeting CIDAMPs might have a higher potential for topical use as antiseptics and disinfectants, in particular at acidic conditions, which could be relevant in cystic fibrosis where air surface liquid is acidic^76^.

*S. aureus* is a widespread cutaneous pathogen responsible for the majority of bacterial skin infections in humans. The treatment of *S. aureus* is marked by development of resistance to each new class of anti-staphylococcal antimicrobial drugs^77^. Thus alternative approaches to the treatment of *S. aureus* infection, in particular MRSA, are urgently sought^78^. Our exploratory study identified a number of candidate CIDAMPs with potent activity also towards MRSA (Table 9). Here, Pal-GR13, and its putatively protease-stabile^79^ all-(D)-variant, were identified as the most promising CIDAMPs that retained activity under test conditions recommended by CLSI.

One of the most intriguing findings to arise from the CO-ADD-screening of our CIDAMP library was the observation that 23 of 69 investigated CIDAMPs were inhibiting *Cryptococcus neoformans* (Table 9). Cryptococcosis is an opportunistic invasive fungal infection, which can result in life-threatening infections of the central nervous system. The disease remains responsible for considerable morbidity and mortality and, despite advances in the standard of medical care and the introduction of Amphotericin B more than half a century ago, the management of cryptococcosis remains unsatisfactory^80^. Current therapeutic options are limited to therapeutics that exhibit significant toxicity and are widely unavailable in resource-limited regions. Additionally, resistance mechanisms against these drugs have evolved. As a possible alternative, CIDAMPS like LCE-peptides could present as promising candidates in the urgent race for therapeutic alternatives for cryptococcosis^81^.

In summary, our data leave little doubt about the great potential of CIDAMPs to form the basis of a novel class of broad spectrum and target-specific bactericidal and fungicidal anti-infectives. These could be used locally as versatile disinfectants, antiseptics and possibly, at least in part, also systemically. Furthermore, CIDAMPs may be added to the large number of antibiotics targeting ribosomes at distinct locations within functionally relevant sites, exerting their inhibitory action by diverse modes^82^. It is further suggested that usage of designer CIDAMPs, which are composed from degradable chemical components, should hardly cause ecological or environmental contamination, as proposed for newly designed ribosomal antibiotics^83^.

Although development of resistance can be a major problem for any newly designed antimicrobial agent, this seems be very difficult for CIDAMP-challenged bacteria since HRNR-derived CIDAMPs are simultaneously targeting multiple ribosomal proteins^40^. Possibly as consequence and/or CIDAMP’s intrinsic characteristic to form amyloid-like nanostructures^40^, the result seems to be an induced cell death.

## Material and Methods

### Ethics

Isolation of human stratum corneum from the heel of healthy donors was performed according to Helsinki guidelines and with appropriate protocols approved by the Ethics Committee at the Medical Faculty of the Christian-Albrechts-University, Kiel (AZ 104/06). Only anonymized material, which has been pooled and stored below −78 °C for 20 years, was used in this study and research personnel received and used these samples anonymously. The same source of pooled heel stratum corneum has been already used for our previous studies^12,84–87^.

### Synthetic peptides

Peptides were purchased as trifluoracetic acid (TFA)-salts from Genecust Europe (Luxembourg). When necessary, peptides were further purified by RP-HPLC, adopting conditions successfully used for purification of antimicrobial peptides^11^. The identity of the peptides was confirmed by ESI-MS analyses and its purity was better than 95% as determined by RP-HPLC and mass spectrometry.

Whenever possible, peptides were dissolved in 0.01% (v/v) aqueous acetic acid and stored as a stock at 3 mg/ml at −20 °C until further use and dilutions were always freshly prepared. Some palmitoylated peptides were dissolved in DMSO (30% (v/v) in 0.01% (v/v) aqueous acetic acid, 30 mg/ml), immediately diluted with 0.01% (v/v) aqueous acetic acid and stored as a stock at 3 mg/ml at −20 °C until further use. Dilutions were always freshly prepared and checked for the presence of precipitates. If these were observed, samples were discharged. When Cys-containing LCE-peptides have been dissolved in DMSO, dilutions were kept cool to minimize thiolate-oxidation. DMSO-concentrations were always ≤0.3%.

### Recombinant expression of HRNR peptide fragments

Seven recombinant hornerin polypeptides (rHRNR_1050–1172_; rHRNR_2576–2707_; rHRNR_2591–2684_; rHRNR_2591–2644_; rHRNR_2638–2684_; rHRNR_2656–2684_; rHRNR_2727–2850_) were expressed (Supplementary Table 1). First we attempted to generate HRNR repeat-domain peptides using a thioredoxin-reductase-(His)_6_-HRNR fusion protein, which had to be cleaved by enterokinase to liberate the full length HRNR-peptide. To subclone into the expression vector pET-32a (Novagen, North Ryde, Australia), PCR was performed with *Pfu* DNA polymerase (Promega, Mannhein, Germany) under the following conditions: 45 s at 98 °C; 5 cycles (45 s at 98 °C; 45 s at 55 °C; 1 min at 72 °C); 25 cycles (45 s at 98 °C; 1 min at 72 °C). The inserts were cut with *Bgl* II and *Not* I, gel purified and inserted into the pET-32a vector that had been double-digested with *Bgl* II and *Not* I. Although the fusion protein could be generated, all of our attempts to generate full length HRNR polypeptides failed. In particular the HRNR_2591–2684_-fusion protein was found to be extremely sensitive towards treatment with enterokinase resulting in excessive degradation. Using a different strategy we could generate defined HRNR polypeptides from SUMO3-His-tag-fusion proteins. To subclone into the expression vector pET-SUMO3 (Invitrogen), PCR was performed with *Pfu* DNA polymerase for 30 cycles (45 s at 98 °C; 45 s at Tm-5 °C; 1 min at 72 °C). The inserts were gel purified and inserted into the pET-SUMO vector. Specific primer pairs used in this study are listed in Supplementary Table 15. All positive clones were identified and verified by sequencing. The plasmids were introduced into the *E. coli* host strain BL21(DE3)pLysS (Novagen). Subsequently, these were grown at 37 °C in tryptic soy broth (TSB) medium containing appropriate antibiotics. Expression of the recombinant protein was induced with 1 mM isopropyl thio-β-D-galactoside (IPTG) for 3 h at 37 °C. Bacteria were harvested by centrifugation at 5,000 × *g* for 5 min at 4 °C, lysed by sonication and then centrifuged at 18,000 × *g* for 30 min at 4 °C (Beckman Coulter, Krefeld, Germany). Recombinant proteins were trapped with Ni^2+^ prepared columns (Macherey-Nagel, Dueren, Germany) and Ni^2+^-affinity column-bound proteins were subjected to preparative reversed phase high-performance liquid chromatography (RP-HPLC) with a column (SP250/10 Nucleosil 300-7 C8; Macherey-Nagel) that was previously equilibrated with 0.1% (v/v) TFA in HPLC-grade water containing 10% acetonitrile. The polyhistidine-tagged fusion proteins were eluted with a gradient of increasing concentrations of acetonitrile containing 0.1% (v/v) TFA (flow rate, 3 ml/min). Fractions containing UV (215 nm)-absorbing material were collected, lyophilized and analyzed by ESI-QTOF-mass spectrometry (Micromass, Manchester, U.K.). Purified histidine-tagged SUMO3-fusion proteins were then digested with SUMO protease 1 (Lifesensors Inc., Pennsylvania, USA) according to the manufacturer’s suggestion. The target peptide was purified by RP-HPLC with a Jupiter-5µ-C4-300A HPLC column (Phenomenex, Aschaffenburg, Germany) equilibrated with 0.1% (TFA) in 10% acetonitrile. Peptides were eluted with a gradient of increasing concentrations of acetonitrile containing 0.1% (v/v) TFA (flow rate, 0.5 ml/min). Fractions of each peak were collected. Purity of recombinant fusion-proteins was determined by SDS-PAGE. Briefly, proteins were separated on NuPAGE^®^ Novex 10% Bis-Tris gels with MES SDS buffer (Invitrogen). Fusion proteins in Bis-Tris gels were stained either with silver nitrate (Sigma) or Coomassie blue R-250 (Sigma). The SeeBlue^®^ Plus 2 Pre-stained Standard marker (Invitrogen) was used as molecular weight markers. The polypeptide purity and molecular masses were assessed using QTOF-ESI-MS.

Further details (in german) are available from (http://macau.uni-kiel.de/receive/dissertation_diss_00018004).

### Proteases used in this study

human α-Chymotrypsin (Sigma); Collagenase NB 4G Proved Grade (Serva); Elastase from human leukocytes (Sigma); Enterokinase EKMax (Invitrogen); Glu-C (Roche); kallikrein 1, kallikrein 2, kallikrein 3, kallikrein 4, kallikrein 5 (stratum corneum tryptic enzyme - SCTE), kallikrein 7 (stratum corneum chymotryptic enzyme - SCCE), kallikrein 8, kallikrein 13 und kallikrein 14: (R&D); Lys-C (Roche); modified (methylated) trypsin (Roche); plasmin from human plasma (Sigma); proteinase K (Invitrogen); SUMOProtease 1 und 2 (LifeSensors); Thermolysin (R&D); thrombin from human serum (Sigma); Trypsin (Roche).

### Other enzymes used in this study

Human protein-arginine deiminase (PAD1) (Cayman).

### Microbes used in this study

*Acinetobacter baumannii ATCC 19606*

*Burkholderia cepacia ATCC 25416*

*Candida albicans ATCC 2443*

*Clostridium perfringens* ATCC 13124

*Corynebacterium simulans DSM 44415*

*Enterococcus faecium DSM 2146*

*Escherichia coli ATCC11775*

*Finegoldia magna* ATCC 15794

*Klebsiella pneumoniae* ATCC 13883

*Lactobacillus crispatus* DSM 20584

*Moraxella osloensis* RV A2/2001

*Peptostreptococcus magnus* ATCC 15794

*Prevotella oralis* ATCC 33321

*Propionibacterium acnes* ATCC 6919

*Proteus mirabilis ATCC 21100*+

*Pseudomonas aeruginosa ATCC 10145*

*Pseudomonas aeruginosa ATCC 11446*

*Salmonella typhimurium* ATCC 13311

*Staphylococcus aureus ATCC 6538*

*Staphylococcus epidermidis ATCC*

*Staphylococcus hominis* ATCC 27844

*Streptococcus pneumoniae ATCC 33400*

*Streptococcus pyogenes ATCC 12344*

### Microbial growth conditions

Bacteria were cultivated in either brain heart infusion medium (BHI), lysogeny broth (LB) or tryptic soy broth (TSB)^88^. If not otherwise stated, bacteria were incubated under shaking conditions (37 °C at 170 rpm) or as indicated on the ATCC- or DSM-data sheets. *Candida albicans* was cultured for 3 days on Yeast Extract-Peptone Dextrose (YPD) agar at 30 °C and yeast suspensions at appropriate density were treated with CIDAMPs as indicated.

### Preparative reversed phase (RP8)-HPLC of heparin-bound heel stratum corneum proteins

Isolation and purification of cationic peptides and proteins was performed as previously described^84,85,89^. Pooled heel stratum corneum, which has been stored below −78 °C as surplus from a previous study^12^, was extracted with acidic ethanolic citrate buffer as described^12,86,90^ and extracts stored as aliquots below −78 °C until further use. After diafiltration (Amicon filters, cut off: 3 kDa) against 10 mM Tris/citrate buffer, pH 8.0, extracts were then applied to a heparin-sepharose cartridge (10 × 5 mm, Pharmacia, Freiburg, FRG), previously equilibrated with the diafiltration buffer. After washing, bound proteins were eluted with 2 ml 2 M NaCl in 0.1 M Tris/citrate buffer and the heparin-bound material was then diafiltered against 0.1% (v/v) TFA in HPLC grade water.

Heparin-bound material was separated by preparative wide-pore reversed phase high-performance liquid chromatography (RP-HPLC) using a column (300 × 7 mm, C8 Nucleosil, 250 × 12.6 mm, Macherey and Nagel, Düren, FRG) that was previously equilibrated with 0.1% (v/v) TFA in HPLC grade water containing 20% (v/v) acetonitrile (eluent A). Proteins were eluted with a gradient of increasing concentrations of acetonitrile containing 0.1% (v/v) TFA (eluent B, flow rate: 2 ml/min). Aliquots (30 μl) of each fraction were lyophilized, dissolved in 5 μl 0.1% (v/v) aqueous acetic acid and tested for antimicrobial activity by a radial diffusion plate assay.

Polar peptides-containing fractions eluting at 10–14% eluent A (and inhibiting *P. a*. at pH 5.5, Fig. 1 in this study) have been previously analysed by N-terminal amino acid sequencing and ESI-MS, as described^12,85^.

### In-house- HRNR-ELISA of RP-HPLC-fractions

20 µl aliquots of acetonitrile-containing fractions from a preparative RP8-HPLC-run of heparin-bound proteins off a stratum corneum extract^11^; for a RP-HPLC run, see Fig. 1) were lyophilized and the residues dissolved in 5 µl distilled water. 4 µl of each sample were diluted with 45 µl 0.1 M Na-phosphate buffer (NaP), pH 7.4, and then added to an ELISA-microtiter plate (Nunc). The HRNR-ELISA-microtiter plate has been prepared by coating over night with pooled affinity-purified goat polyclonal HRNR antibodies (antigens: HRNR_1075–1172_, HRNR_2591–2662_, HRNR_2726–2850_)^12^, stored at 1 mg/ml as pool below −78 °C and used at 1:200 dilution in coating buffer, blocking with 1% (w/v) freshly prepared, biotin-free BSA for 90 min, and three times washing with PBS-Tween (PBS-T, 0.05%, v/v). Samples and standards (rHRNR_1075–1172_ (0–500 ng/ml 0.1 M Na-P buffer, pH 7.4) were added to the ELISA plate, incubated for 45 min at 37 °C, followed by adding biotinylated pooled polyclonal HRNR antibodies (see above, from a 1 mg/ml pool), at 1:200 dilution in PBS-T (0.05%, v/v) and a 45 min at 37 °C incubation. After three-fold washing in PBS-T, streptavidin-peroxidase conjugate (1:10,000) was added, incubated for 30 min and after 6-fold washing with PBS, peroxidase substrate (T-ABTS- solution) was added and monitored at 405 nm and 492 nm.

### HRNR-Immuno-Dot-blot-analyses

30 µL of RP-8-HPLC fraction (Fig. 1) were lyophilized in a microtiter plate, the residues dissolved in 5 µL water and 2 µL applied to a nitrocellulose membrane (0.2 µm-pore size; Bio-Rad). After blotting, the membrane was saturated at room temperature for 1 h with PBS/M [1 × PBS containing 5% (wt/vol) freeze-dried low-fat milk] and then washed three times with PBS/T [1 × PBS containing 0.05% (vol/vol) Tween 20]. The membrane was incubated at room temperature for 1 h with affinity-purified goat HRNR antibodies (antigens: HRNR_1075–1172_, HRNR_2591–2662_, HRNR_2726–2850_)^12^ at 10 µg/ml in PBS/T/M (1 × PBS containing 0.05% Tween and 5% freeze-dried low-fat milk). After three washes with PBS/T, the membrane was incubated at room temperature for 1 h with rabbit anti-goat immunoglobulin G (heavy plus light chains)-horseradish peroxidase conjugate (Bio-Rad) diluted 1: 30,000 in PBS/T/M. The membrane was then washed three times with PBS/T and twice with PBS. The substrate (SuperSignal West Dura extended duration substrate; Pierce) was deposited for 5 min onto the membrane. The chemiluminescence was monitored by a CD-camera (Peqlab, Raytest).

### Radial diffusion antimicrobial assay

10 µl aliquots of HPLC fractions were lyophilized in a microtiter plate, the residues dissolved in water and applied to a low electro-osmosis agarose plate containing *Pseudomonas aeruginosa* ATCC 10145 in a modified radial diffusion (RDA) assay, as described^10,11^. The underlay agarose used for the RDA was either prepared in 10 mM NaP, pH 7.4/1% (v/v) tryptic soybean broth (TSB, Oxoid /Thermo Fisher Scientific,Wesel, Germany) or in 10 mM NaP, pH 5.5/ 1% (v/v) TSB and inoculated with approximately 10^5^ colony forming units (CFU) per ml. After adding the samples, plates were incubated over night for 16 h. Thereafter, an overlay-agarose (1% (w/v) agarose, 3.4% (w/v) casein peptone, 0.6% (w/v) soybean peptone, 0.5% D(+)- glucose-mono-hydrate, 1% (w/v) NaCl, 0.5% (w/v) KH_2_PO_4_, pH 7.2–7.4) was added and after an additional 3 h incubation at 37 °C, the diameter of clearing zones were measured.

### Colony-Forming Unit (CFU) assay of antimicrobial activity

All purified peptides and recombinant proteins were applied using a colony forming unit (CFU) assay in different media. Bacteria were grown to early log-phase in BHI, washed either with 10 mM Na-P, pH 7.4/1% TSB or pH 5.5/1% TSB, or pH 5.5 without TSB or BHI, and adjusted to a concentration of 10^4^–10^5^ colony forming units (CFU)/ml. For cell number control, 100 µl aliquots were diluted 1:10, 1:100 and 1:1000 and plated in duplicates on BHI agar plates. To 100 µl aliquots of the bacteria, suspended in the respective medium (10 mM Na-P buffer pH 7,0–7,4/ 1% (w/v) TSB; 10 mM Na-P buffer pH 5,5/ 1% (w/v) TSB; 10 mM Na-P buffer pH 7,0–7,4/ 1% (w/v) TSB pH 7,0–7,4/ 0,25% (w/v) glucose; 10 mM Na-P buffer pH 5,5/ 0,25% (w/v) glucose, respectively), 10 µl of a solution of the peptide or protein (two-fold dilutions, from 300 µg/ml or 150 µg/ml down to 19 ng/ml), dissolved in 0.01% (v/v) acetic acid, were added.

Then bacteria were incubated for 2 h at ambient temperature, if not otherwise stated. 10 µl of 0.01% (v/v) acetic acid, added to 100 µl aliquots of the bacteria in the respective medium, served as negative control. From each test, 1:10 and 1:100 dilutions were generated and plated as duplicates of 100 µl aliquots on BHI agar plates. After 16 h incubation at 37 °C, CFU were counted using a colony counter (Bio Kobe, Japan) and expressed as mean of the duplicate estimation to achieve a complete dose-response curve. All experiments were performed at serial dilutions in a concentration range of 100 µg/mL −25 ng/mL for recombinant HRNR fragments and 300 or 150 µg/mL–19 ng/mL for synthetic peptides. The peptide concentrations showing 10% or 0% CFU of the control ((acidic) medium-treated bacteria) were given as concentration killing 90% (LD90) and 100% (LD100) of the inoculum, respectively.

### Nano-LC-ESI MS and peptide identification

Lyophilized samples of primary 20 µL of pooled polar RP-8-HPLC fractions (fractions 10–20, Fig. 1) of pooled stratum corneum extracts were reconstituted in 50 µL loading buffer (0.1% TFA/3% ACN), diluted 1:10 and injected into a Dionex U3000 nano-LC system (Dionex, Idstein, Germany) coupled online to a Q Exactive Orbitrap mass spectrometer (Thermo Fisher Scientific, Bremen, Germany). Peptides were desalted on a trap column (Acclaim Pepmap C-18, 300 µm × 5 mm, 5 µm, 100 Å, Dionex) at a flow-rate of 30 µL/min with loading buffer for 4 min before being eluted onto an analytical column (Acclaim Pepmap C-18, 75 µm × 500 mm, 3 µm, 100 Å, Dionex) at a flow-rate of 300 nL/min. For peptide elution and separation, a linear gradient with eluent A (0.05% FA) and eluent B (0.04% FA in 80% ACN) was run. The content of B was adjusted as follows: 4–25% B in 300 min, 25–90% B in 6 min, 90% B for 10 min, 90–4% B in 0.1 min and 4% B for 15 min. MS data were recorded from 10 to 320 min. MS full scans at a resolution of 70,000 was acquired between 300 and 2,000 m/z. For every MS spectrum obtained, the 10 most intense precursors with a charge state of ≥2+ were isolated and fragmented using HCD and a normalized collision energy of 30% applied (isolation width was set to 3 m/z). At MS/MS spectra resolution was set to 17,500. After fragmentation, precursors were excluded from further isolation for 15 sec.

Peptides were identified using the Proteome Discoverer software (version 1.4, Thermo Fisher) and the search engine Mascot (version 2.2.07, Matrix Science, London, UK). All precursors between 350 and 5,000 Da with a signal-to-noise ratio of at least 1.5 were considered. The search was performed against a FASTA database of the human proteome including isoforms (36,244 sequences, 2012-09-17) with no enzymatic specificity selected. Precursor and fragment mass tolerances were set to 10 ppm and 0.02 Da, respectively. Oxidation of methionine residues was set as dynamic modification. All sequences were also searched against a decoy list of peptides with a strict (0.01) and relaxed (0.05) false discovery rate (FDR). For identification, only high confident peptide spectrum matches were allowed.

### Protease sensitivity testing of HRNR

10 µg rHRNR_2576–2707_, dissolved in 20 µl proteolysis buffer (100 mM Tris pH 7.5, 150 m NaCl, 5 mM EDTA, 0.05% Tween 20) were incubated with the respective enzymes, dissolved in 10 µl proteolysis buffer, for 18 h at 37 °C and then further analyzed by SDS-PAGE-analyses and HRNR-Westernblot-analyses. Further details (in german) are available from (http://macau.uni kiel.de/receive/dissertation_diss_00018004).

### SDS-PAGE-analyses and protein staining

Electrophoretic mobility was investigated using 16.5% SDS-polyacrylamide gels (SDS-PAGE) in the presence of 8 M urea and Tricine^91^ under nonreducing conditions, as described for chemokines and antimicrobial peptides^11,92^. Peptides were visualized by silver staining. Further details (in german) are available from (http://macau.uni-kiel.de/receive/dissertation_diss_00018004).

### HRNR-Western-blot-analysis

For Western-blot analysis, samples were loaded onto a 16.5% SDS-tricine polyacrylamide gel containing 8 M urea and were transfered to a nitrocellulose membrane (pore size: 0.2 µm, Schleicher & Schuell BioScience, Dassel, Germany) or polyvinylidenfluoride membrane (pore size: 0.2 µm, GE Healthcare) using an alkaline transfer buffer (48 mM Tris, 39 mM g-NaPlycine, 0.0375% (w/v) SDS and 20% EtOH (pH 9.2)). An alkaline transfer buffer is essential to get HRNR-fragments transferred to a membrane^12^. Thereafter, the membrane was blocked for 1 h in blocking buffer (5% (w/v) nonfat powdered milk in PBS + 0.05% Tween), then incubated for 18 h at 4 °C in 3% (w/v) nonfat powdered milk in PBS + 0.05% Tween containing 1:2,000 polyclonal HRNR antibody with affinity-purified goat HRNR antibodies (antigens: HRNR_1075–1172_, HRNR_2591–2662_, HRNR_2726–2850_)^12^. The membrane was washed with PBS + 0.05% Tween six times for 5 min each, then incubated for 1 h in 3% (w/v) nonfat powdered milk in PBS + 0.05% Tween containing 1:20,000 dilution of rabbit anti-goat IgG HRP conjugate (Dianova). After six times washing steps as before, immunoreactive HRNR was visualized by incubation with a peroxidase substrate (Roche LumiLight) at ambient temperature and documented with a „Diana III Digital CCD Imaging System“ or „FUSION FX7“. Further details are available from (http://macau.uni-kiel.de/receive/dissertation_diss_00018004).

### Sample preparation for CO-ADD antimicrobial assays

Samples were provided as dry material and made up to 10 mg/mL in DMSO or water solution and stored frozen at −20 °C. An aliquot of each sample was diluted to 320 μg/mL and plated in 384-well polypropylene plates. 5 μL were plated in duplicate into a 384-well non-binding surface plate (NBS) for each strain or cell type assayed against. Once cells were added, this gave a final compound concentration range of 32 μg/mL. Final Sample Concentrations: 32 μg/mL. Final DMSO Concentration: ≤0.3%. Compound preparation: in Water/DMSO.

### Microbial Strains used for CO-ADD antimicrobial assays

*Escherichia coli* ATCC 25922: FDA control strain

*Klebsiella pneumoniae* ATCC 700603: MDR

*Acinetobacter baumannii* ATCC 19606: Type strain

*Pseudomonas aeruginosa* ATCC 27853: Quality control strain

*Staphylococcus aureus* ATCC 43300: MRSA

*Candida albicans* ATCC 90028: CLSI reference

*Cryptococcus neoformans* ATCC 208821: H99 - Type strain

### Antibacterial assays performed by CO-ADD

All bacteria were cultured in Cation-adjusted Mueller Hinton broth (CAMHB) at 37 °C overnight. A sample of each culture was then diluted 40-fold in fresh broth and incubated at 37 °C for 1.5–3 h. The resultant mid-log phase cultures were diluted (CFU/mL measured by OD600), then 45 μL was added to each well of the compound containing plates, giving a cell density of 5 × 105 CFU/mL and the nominated final compound concentration. All plates were covered and incubated at 37 °C for 18 h without shaking. Inhibition of bacterial growth was determined measuring absorbance at 600 nm (OD600), using a Tecan M1000 Pro monochromator plate reader. The percentage of growth inhibition was calculated for each well, using the negative control (media only) and positive control (bacteria without inhibitors) on the same plate as references. The significance of the inhibition values was determined by Z-scores, calculated using the average and standard deviation of the sample wells (no controls) on the same plate. Samples with inhibition value above 80% and Z-Score above 2.5 for each replicate (n = 2 on different plates) were classed as actives.

Screening has been performed by CO-ADD. For detailed information see http://www.co-add.org/content/free-screening.

### Antifungal assays performed by CO-ADD

Fungi strains were cultured for 3 days on Yeast Extract-Peptone Dextrose (YPD) agar at 30 °C. A yeast suspension of 1 × 10^6^ to 5 × 10^6^ cells/mL (as determined by OD530) was prepared from five colonies. These stock suspensions were diluted with Yeast Nitrogen Base (YNB) broth to a final concentration of 2.5 × 10^3^ CFU/mL. Then, 45 μL of the fungi suspension was added to each well of the compound-containing plates. Plates were covered and incubated at 35 °C for 24 h without shaking.

Screening has been performed by CO-ADD. For detailed information see http://www.co-add.org/content/free-screening.

### Antibiotic standards preparation and quality control of CO-ADD-assays

Colistin and Vancomycin were used as positive bacterial inhibitor standards for Gram-negative and Gram-positive bacteria, respectively. Fluconazole was used as a positive fungal inhibitor standard for *C. albicans and C. neoformans*. The antibiotics were provided in 4 concentrations, with 2 above and 2 below its MIC value, and plated into the first 8 wells of column 23 of the 384-well NBS plates.

The quality control (QC) of the assays was determined by the antimicrobial controls and the Z’-factor (using positive and negative controls). Each plate was deemed to fulfil the quality criteria (pass QC), if the Z’-factor was above 0.4, and the antimicrobial standards showed full range of activity, with full growth inhibition at their highest concentration, and no growth inhibition at their lowest concentration. For detailed information see http://www.co-add.org/content/free-screening.

### Transmission electron microscopy

All Transmission electron microscopy (TEM) imaging was performed by the Christian-Albrechts-University (CAU) Kiel Center of Biologic Imaging Core at the microscopy core facility. Logarithmic grown microorganisms were concentrated at ambient temperature to an OD_600nm_ of 4 in 10 mM NaP, pH 7.4/1% TSB or 10 mM NaP, pH 5.5/0.25% glucose, washed with the respective medium and then suspended to an OD_600nm_ of 2.0, depending on the species, corresponding to 10^9^–10^10^ microorganisms. The amount of the CIDAMPs used was about 2 × 10^7^ molecules per colony forming unit (CFU). Microorganisms were then incubated with CIDAMPs either at ambient temperature or at 37 °C for 120 min (Pal-GR13) or 90 min (LCE3B_56–68_) in the respective media, which also served as controls to identify medium effects. Microorganisms were then fixed in 2.5% glutaraldehyde at 4 °C overnight. Bacteria were centrifuged at 3220 × g for 10 min, supernatants discarded and then the pelleted samples were suspended at 44 °C (in a thermoblock) in a vial containing 2% Noble Agar in distilled water. Samples in the vial were centrifuged, cooled to 4 °C and the bacteria-containing agar-block taken from the tip of the vial. This was then dehydrated in an ascending graded ethanol (EtOH) series. For embedding, the EtOH was replaced stepwise by a polyhydroxy-aromatic acrylic resin (LR White), starting at a resin:EtOH ratio of 1:2, followed by 1:1, 2:1, and three times in resin only, each for 30 min. Finally, samples were embedded in resin at 60 °C. The hardened resin was then cut into 5 nm sections and transferred onto a grid. All samples were analyzed with a transmission electron microscope (Philips TEM 208 or FEI Tecnai G2 Spirit BioTwin).

## Supplementary information

Dataset 1
