# Supplementary material for: Cationic Intrinsically Disordered Antimicrobial Peptides (CIDAMPs) Represent a New Paradigm of Innate Defense with a Potential for Novel Anti-Infectives
Source: Sci Rep. 2019 Mar 4;9:3331. doi: 10.1038/s41598-019-39219-w (PMC6399351; doi:10.1038/s41598-019-39219-w)
Supplement: Supplementary file 1 — Dataset 1 [file 41598_2019_39219_MOESM1_ESM.pdf]

SUPPLEMENTARY MATERIAL

Cationic Intrinsically Disordered Antimicrobial Peptides (CIDAMPs) Represent a New Paradigm of Innate Defense with a Potential for Novel Anti-Infectives

Ties Latendorf[1,§] , Ulrich Gerstel[1,§], Zhihong Wu[1,□], Joachim Bartels[1], Alexander Becker[2], Andreas Tholey[2], Jens-Michael Schröder[1,*]

1 Dpt. of Dermatology, University-Hospital Schleswig-Holstein, Campus Kiel, Kiel, Germany

2 Institute for Experimental Medicine – AG Systematic Proteomics & Bioanalytics, Kiel University (CAU), Kiel, Germany

* corresponding author

□ present address: Institute of Biochemistry and Cell Biology, Zhejiang University of Science and Technology, 310023 Hangzhou, China

§ equal contribution

Corresponding author: Jens-M. Schröder, University-Hospital Schleswig-Holstein, Dpt. of Dermatology, Rosalind-Franklin-Str. 7, D-24105 Kiel, Germany, +49 431 500 – 2101, FAX – 21104, Email: jschroeder@dermatology.uni-kiel.de

Supplementary Figure 1

```
       340        350        360        370        380        400        410        420        430        440        450        460
YSYSRGHYES GSGQTSGFGQ HESGSGQSSG YSKHGSGSGH SSSQGQHGST SGQASSSGQH GQHESASRHS SGRGQHSSGS GQSPGHGQRG SGSGQSPSSG QHGTGFGRSS SSGPYVSGSG
    SRGHYES GSGQTSGFGQ HESGSGQSSG Y                                       HS SGRGQHSSGS GQSPGHGQRG SGSGQSPSSG QHGTGFGRSS SSGPY
    SRGHYES GSGQTSGFGQ HESGSGQSSG                                         HS SGRGQHSSGS GQSPGHGQRG SGSGQSPSSG QHGTGFGRSS SSGP
         ES GSGQTSGFGQ HESGSGQSSG Y                                       HS SGRGQHSSGS GQSPGHGQRG SGSGQSPSSG QHGTGFGR
                                                                          HS SGRGQHSSGS GQSPGHGQRG SGSGQSPSSG QHGTGFGR
                                                                          HS SGRGQHSSGS GQSPGHGQRG SGSGQSPSSG QHGTGFG
                                                                             SGRGQHSSGS GQSPGHGQRG SGSGQSPSSG QHGTGFGR
                                                                              GRGQHSSGS GQSPGHGQRG SGSGQSPSSG QHGTGFG
                                                                              GRGQHSSGS GQSPGHGQRG SGSGQSPSSG QHGTGF
                                                                                   SSGS GQSPGHGQRG SGSGQSPSSG QHGTGFGR
                                                                                     GS GQSPGHGQRG SGSGQSPSSG QHGTGFG

       500        510        520        530        540        550        560        570
HGQHGSRSGQ SSRGERQGSS AGSSSSYGQH GSGSRQSLGH SRHGSGSGQS PSPSRGRHES GSRQSSSYGP HGYGSGRSSS
       SGQ SSRGERQGSS AGSSSSYGQH GSGSRQSLGH S HGSGSGQS PSPSRGRHES GSRQSSSYGP HG
              GERQGSS AGSSSSYGQH GSGSRQSLGH S
              GERQGSS AGSSSSYGQH GSGSRQSL

       680        690        700        710        720        730        740        750        760        770        780
HSSSYGQHGS GSGWSSSNGP HGSVSGQSSG FGHKSGSGQS SGYSQHGSGS SHSSGYRRHG SRSGQSSRSE QHGSSSGLSS SYGQHGSGSH QSSGHGRQGS GSGHSPSRVR
     GQHGS GSGWSSSNGP HGSVSGQSSG FGHKSGSGQS SG                      SGQSSRSE QHGSSSGLSS SYGQHGSGSH QSSGHGRQGS GSGHSPSRVR
                SSSNGP HGSVSGQSSG FGHKSGSGQS SGYSQHGSGS SHSSGY       SGQSSRSE QHGSSSGLSS SYGQHGSGSH QSSGHGR
                SSSNGP HGSVSGQSSG FGHKSGSGQS SGY                           SE QHGSSSGLSS SYGQHGSGSH QSSGHGRQGS GSGHSPS
                SSSNGP HGSVSGQSSG FGHKSGSGQS SG
                 SSNGP HGSVSGQSSG FGHKSGSGQS SGYS
                 SSNGP HGSVSGQSSG FGHKSGSGQS SGY
                  SNGP HGSVSGQSSG FGHKSGSGQS SGY
                   NGP HGSVSGQSSG FGHKSGSGQS SGY
                              SSG FGHKSGSGQS SGYSQHGSGS SHSSGY

       920        930        940        950        960        970
SGSGRSSSSG RHGSGSGQSS GFGHKSSSGQ SSGYTQHGSG SGHSSSYEQH GSRSGQSSRS
     SSSSG RHGSGSGQSS GFGHKSSSGQ SSGYTQHGSG SGHSSSYEQH GS
     SSSSG RHGSGSGQSS GFGHKSSSGQ SSGYTQHG
     SSSSG RHGSGSGQSS GFGHKSSSGQ SSGYTQH
     SSSSG RHGSGSGQSS GFGHKSSSGQ SSGYT
     SSSSG RHGSGSGQSS GFGHKSSSGQ SSGY
       SSG RHGSGSGQSS GFGHKSSSGQ SSGYTQH

       980        990       1000       1010       1020       1030       1040       1050       1060       1070       1080
EQHGSSSGSS SSYGQHGSGS RQSLGHGQHG SGSGQSPSPS RGRHGSGSGQ SSSYGPYRSG SGWSSSRGPY ESGSGHSSGL GHRESRSGQS SGYGQHGSSS GHSSTHGQHG
  HGSSSGSS SSYGQHGSGS RQSLGHGQHG SGSGQSPSPS                             RGPY ESGSGHSSGL GHRESR
  HGSSSGSS SSYGQHGSGS RQSL                                                   ESGSGHSSGL GHRESRSGQS SGYGQHGSSS GHSSTHGQHG
                       QSLGHGQHG SGSGQSPSPS RGRHGSGSGQ SSSY
                          GHGQHG SGSGQSPSPS RGRHGSGSGQ SSSYGPY
                          GHGQHG SGSGQSPSPS RGRHGSGSGQ SSSYGP
                           HGQHG SGSGQSPSPS RGRHGSGSGQ SSSYGPY
                              HG SGSGQSPSPS RGRHGSGSGQ SSSYGPY
                               G SGSGQSPSPS RGRHGSGSGQ SSSYGPY
                                 SGSGQSPSPS RGRHGSGSGQ SSSYGPY
                                 SGSGQSPSPS RGRHGSGSGQ SSSYGP

      1110       1120       1130       1140       1150       1160       1170       1180       1190       1200
SHGQHGSGSS QSSGYGRQGS GSGQSPGHGQ RGSGSRQSPS YGRHGSGSGR SSSSGQHGSG LGESSGFGHH ESSSGQSSSY SQHGSGSGHS SGYGQHGSRS
                                       QSPS YGRHGSGSGR SSSSGQHGSG LGESSGFGHH ESSSGQSSS
                                       QSPS YGRHGSGSGR SSSSGQHGSG LGESSGFGHH E
                                       QSPS YGRHGSGSGR SSSSGQHGSG LGE
                                             GRHGSGSGR SSSSGQHGSG LGESSGFGHH ESSSGQSSSY
                                             GRHGSGSGR SSSSGQHGSG LGESSGFGHH ESSSGQSSS
                                             GRHGSGSGR SSSSGQHGSG LGESSGFGHH ESSSGQSS
                                             GRHGSGSGR SSSSGQHGSG LGESSGFGHH ESSSGQS
                                             GRHGSGSGR SSSSGQHGSG LGESSGFGHH ESSSG
                                             GRHGSGSGR SSSSGQHGSG LGESSGFGHH ESS
                                             GRHGSGSGR SSSSGQHGSG LGESSGFGHH ES
                                             GRHGSGSGR SSSSGQHGSG LGESSGFGHH E
                                              RHGSGSGR SSSSGQHGSG LGESSGFGHH ESSSGQSSS
                                               HGSGSGR SSSSGQHGSG LGESSGFGHH ESSSGQSSS
                                                GSGSGR SSSSGQHGSG LGESSGFGHH ESSSGQSSS
                                                       SSSSGQHGSG LGESSGFGHH ESSSGQSSSY SQHGSGSGHS SGYGQHGS
                                                       SSSSGQHGSG LGESSGFGHH ESSSGQSSSY
                                                       SSSSGQHGSG LGESSGFGHH ESSSGQSSS
                                                       SSSSGQHGSG LGESSGFGHH ESSSGQSS
                                                       SSSSGQHGSG LGESSGFGHH ESSSGQS
                                                       SSSSGQHGSG LGESSGFGHH ESSSGQ
                                                       SSSSGQHGSG LGESSGFGHH ESSSG
                                                       SSSSGQHGSG LGESSGFGHH ESSS
                                                       SSSSGQHGSG LGESSGFGHH ESS
                                                       SSSSGQHGSG LGESSGFGHH ES
                                                       SSSSGQHGSG LGESSGFGHH E
                                                       SSSSGQHGSG LGESSGFGHH
                                                         SSGQHGSG LGESSGFGHH ESSSGQSSS
                                                          SGQHGSG LGESSGFGHH ESSSGQSSS
                                                           GQHGSG LGESSGFGHH ESSSGQSSS
                                                             HGSG LGESSGFGHH ESSSGQSSS
```

Supplementary Figure 1, continued

```
      1250       1260       1270       1280       1290       1300
HSPSRGRHGS GLGHSSSHGQ HGSGSGRSSS RGPYESRSGH SSVFGQHESG SGHSSAYSQH
     GRHGS GLGHSSSHGQ HGSGSGRSSS RGPYE
     GRHGS GLGHSSSHGQ HGSGSGRSSS RGPY
       HGS GLGHSSSHGQ HGSGSGRSSS RGPYE
       HGS GLGHSSSHGQ HGSGSGRSSS RGPY
        GS GLGHSSSHGQ HGSGSGRSSS RGPY
           GLGHSSSHGQ HGSGSGRSSS RGPY
                                     ESRSGH SSVFGQHESG SGHSSAY
                                     ESRSGH SSVFGQHESG SGHSSA
                                     ESRSGH SSVFGQHESG SGHS
                                     ESRSGH SSVFGQHESG SGH
                                        SGH SSVFGQHESG SGHSSAY
                                        SGH SSVFGQHESG SGHSSA

      1360       1370       1380       1390       1400       1410
RHGAGSGQSP SRGRHGSGSG HSSSYGQHGS GSGWSSSSGR HGSGSGQSSG FGHHESSSWQ
        SP SRGRHGSGSG HSSSYGQHGS GSGWS
             RGRHGSGSG HSSSYGQHGS GSGW
              GRHGSGSG HSSSYGQHGS GSGWSSSSGR HGSGSGQSSG FGHHES
              GRHGSGSG HSSSYGQHGS GSGWSSSSGR HGSGSGQSSG FGHHE
              GRHGSGSG HSSSYGQHGS GSGWSSSSGR HG
              GRHGSGSG HSSSYGQHGS GSGWSSSSGR
              GRHGSGSG HSSSYGQHGS GSGWS
              GRHGSGSG HSSSYGQHGS GSGW
                HGSGSG HSSSYGQHGS GSGWSSSSGR HGSGSGQSSG FGHHESS
                HGSGSG HSSSYGQHGS GSGWSSSSGR HGSGSGQSSG FGHHES
                HGSGSG HSSSYGQHGS GSGWSSSSGR HGSGSGQSSG FGHHE
                HGSGSG HSSSYGQHGS GSGW
                           GQHGS GSGWSSSSGR HGSGSGQSSG FGHHESSS
                           GQHGS GSGWSSSSGR HGSGSGQSSG FGHHESS
                           GQHGS GSGWSSSSGR HGSGSGQSSG FGHHES
                           GQHGS GSGWSSSSGR HGSGSGQSSG FGHHE
                           GQHGS GSGWSSSSGR HGSGSGQSSG FGHH
                              GS GSGWSSSSGR HGSGSGQSSG FGHHE
                                     SSSSGR HGSGSGQSSG FGHHESSSWQ
                                     SSSSGR HGSGSGQSSG FGHHESSSW
                                        SGR HGSGSGQSSG FGHHESSSW
                                         GR HGSGSGQSSG FGHHESSSWQ
                                            HGSGSGQSSG FGHHESSSW

      1440       1450       1460       1470       1480       1490       1500
GSRSGQSSRG ERHGSSSGSS SSYGQHGSGS RQSLGHGQHG SGSGQSPSPS RGRHGSGSGQ SSSYSPYGSG
         G ERHGSSSGSS SSYGQHGSGS RQSL
             HGSSSGSS SSYGQHGSGS RQSLGHGQHG SGSGQSPSPS
             HGSSSGSS SSYGQHGSGS RQSL
                                  QSLGHGQHG SGSGQSPSPS RGRHGSGSGQ SSSY
                                     GHGQHG SGSGQSPSPS RGRHGSGSGQ SSSYSPY

      1610       1620       1630       1640       1650       1660       1670
RGSGSRQSPS YGRHGSGSGR SSSSGQHGSG LGESSGFGHH ESSSGQSSSY SQHGSGSGHS SGYGQHGSRS
      QSPS YGRHGSGSGR SSSSGQHGSG LGESSGFGHH ESSSGQSSS
      QSPS YGRHGSGSGR SSSSGQHGSG LGESSGFGHH E
      QSPS YGRHGSGSGR SSSSGQHGSG LGE
            GRHGSGSGR SSSSGQHGSG LGESSGFGHH ESSSGQSSSY
            GRHGSGSGR SSSSGQHGSG LGESSGFGHH ESSSGQSSS
            GRHGSGSGR SSSSGQHGSG LGESSGFGHH ESSSGQSS
            GRHGSGSGR SSSSGQHGSG LGESSGFGHH ESSSGQS
            GRHGSGSGR SSSSGQHGSG LGESSGFGHH ESSSG
            GRHGSGSGR SSSSGQHGSG LGESSGFGHH ESS
            GRHGSGSGR SSSSGQHGSG LGESSGFGHH ES
            GRHGSGSGR SSSSGQHGSG LGESSGFGHH E
             RHGSGSGR SSSSGQHGSG LGESSGFGHH ESSSGQSSS
              HGSGSGR SSSSGQHGSG LGESSGFGHH ESSSGQSSS
               GSGSGR SSSSGQHGSG LGESSGFGHH ESSSGQSSS
                      SSSSGQHGSG LGESSGFGHH ESSSGQSSSY SQHGSGSGHS SGYGQHGS
                      SSSSGQHGSG LGESSGFGHH ESSSGQSSSY
                      SSSSGQHGSG LGESSGFGHH ESSSGQSSS
                      SSSSGQHGSG LGESSGFGHH ESSSGQSS
                      SSSSGQHGSG LGESSGFGHH ESSSGQS
                      SSSSGQHGSG LGESSGFGHH ESSSGQ
                      SSSSGQHGSG LGESSGFGHH ESSSG
                      SSSSGQHGSG LGESSGFGHH ESSS
                      SSSSGQHGSG LGESSGFGHH ESS
                      SSSSGQHGSG LGESSGFGHH ES
                      SSSSGQHGSG LGESSGFGHH E
                      SSSSGQHGSG LGESSGFGHH
                        SSGQHGSG LGESSGFGHH ESSSGQSSS
                         SGQHGSG LGESSGFGHH ESSSGQSSS
                          GQHGSG LGESSGFGHH ESSSGQSSS
                            HGSG LGESSGFGHH ESSSGQSSS
```

Supplementary Figure 1, continued

```
      1710       1720       1730       1740       1750       1760       1770       1780       1790
GHGRQGSGSG QSPSRGRHGS GLGHSSSHGQ HGSGSGRSSS RGPYESRSGH SSVFGQHESG SGHSSAYSQH GSGSGHFCSQ GQHGSTSGQS STFDQE
                GRHGS GLGHSSSHGQ HGSGSGRSSS RGPYE
                GRHGS GLGHSSSHGQ HGSGSGRSSS RGPY
                  HGS GLGHSSSHGQ HGSGSGRSSS RGPYE
                  HGS GLGHSSSHGQ HGSGSGRSSS RGPY
                   GS GLGHSSSHGQ HGSGSGRSSS RGPY
                      GLGHSSSHGQ HGSGSGRSSS RGPY
                                                ESRSGH SSVFGQHESG SGHSSAY
                                                ESRSGH SSVFGQHESG SGHSSA
                                                ESRSGH SSVFGQHESG SGHS
                                                ESRSGH SSVFGQHESG SGH
                                                   SGH SSVFGQHESG SGHSSAY
                                                   SGH SSVFGQHESG SGHSSA

      1810       1820       1830       1840       1850       1860       1870       1880
GQSSSHGQHG SGSSQSSSYG QQGSGSGQSP SRGRHGSGSG HSSSYGQHGS GSGWSSSSGR HGSGSGQSSG FGHHESSSWQ
                    G QQGSGSGQSP SRGRHGSGSG HSSSYGQHGS GSGWSSSSGR
                    G QQGSGSGQSP SRGRHGSGSG HSSSYGQHGS GSGWS
                         SGSGQSP SRGRHGSGSG HSSSYGQHGS GSGWSSSSGR
                         SGSGQSP SRGRHGSGSG HSSSYGQHGS GSGWSS
                              SP SRGRHGSGSG HSSSYGQHGS GSGWS
                                  RGRHGSGSG HSSSYGQHGS GSGW
                                   GRHGSGSG HSSSYGQHGS GSGWSSSSGR HGSGSGQSSG FGHHES
                                   GRHGSGSG HSSSYGQHGS GSGWSSSSGR HGSGSGQSSG FGHHE
                                   GRHGSGSG HSSSYGQHGS GSGWSSSSGR HG
                                   GRHGSGSG HSSSYGQHGS GSGWSSSSGR
                                   GRHGSGSG HSSSYGQHGS GSGWS
                                   GRHGSGSG HSSSYGQHGS GSGW
                                     HGSGSG HSSSYGQHGS GSGWSSSSGR HGSGSGQSSG FGHHESS
                                     HGSGSG HSSSYGQHGS GSGWSSSSGR HGSGSGQSSG FGHHES
                                     HGSGSG HSSSYGQHGS GSGWSSSSGR HGSGSGQSSG FGHHE
                                     HGSGSG HSSSYGQHGS GSGW
                                                 GQHGS GSGWSSSSGR HGSGSGQSSG FGHHESS
                                                 GQHGS GSGWSSSSGR HGSGSGQSSG FGHHES
                                                 GQHGS GSGWSSSSGR HGSGSGQSSG FGHHE
                                                 GQHGS GSGWSSSSGR HGSGSGQSSG FGHH
                                                    GS GSGWSSSSGR HGSGSGQSSG FGHHE
                                                           SSSSGR HGSGSGQSSG FGHHESSSWQ
                                                           SSSSGR HGSGSGQSSG FGHHESSSW
                                                              SGR HGSGSGQSSG FGHHESSSW
                                                               GR HGSGSGQSSG FGHHESSSWQ
                                                                  HGSGSGQSSG FGHHESSSW

      1890       1900       1910       1920       1930       1940       1950       1960       1970
SSGYTQHGSG SGHSSSYEQH GSRSGQSSRG EQHGSSSGSS SSYGQHGSGS RQSLGHGQHG SGSGQSPSPS RGRHGSGSGQ SSSYGPYGSG
                                   HGSSSGSS SSYGQHGSGS RQSLGHGQHG SGSGQSPSPS
                                   HGSSSGSS SSYGQHGSGS RQSL
                                                       QSLGHGQHG SGSGQSPSPS RGRHGSGSGQ SSSY
                                                          GHGQHG SGSGQSPSPS RGRHGSGSGQ SSSYGPY
                                                          GHGQHG SGSGQSPSPS RGRHGSGSGQ SSSYGP
                                                           HGQHG SGSGQSPSPS RGRHGSGSGQ SSSYGPY
                                                              HG SGSGQSPSPS RGRHGSGSGQ SSSYGPY
                                                               G SGSGQSPSPS RGRHGSGSGQ SSSYGPY
                                                                 SGSGQSPSPS RGRHGSGSGQ SSSYGPY
                                                                 SGSGQSPSPS RGRHGSGSGQ SSSYGP

      1980       1990       2000       2010       2020
SGWSSSRGPY ESGSGHSSGL GHRESRSGQS SGYGQHGSSS GHSSTHGQHG
      RGPY ESGSGHSSGL GHRESR
           ESGSGHSSGL GHRESRSGQS SGYGQHGSSS GHSSTHGQHG

      2220       2230       2240
RGPYESRSGH SSVFGQHESG SGHSSAYSQH
    ESRSGH SSVFGQHESG SGHSSAY
    ESRSGH SSVFGQHESG SGHSSA
    ESRSGH SSVFGQHESG SGHS
    ESRSGH SSVFGQHESG SGH
       SGH SSVFGQHESG SGHSSAY
       SGH SSVFGQHESG SGHSSA
```

Supplementary Figure 1, continued

```
      2290       2300       2310       2320       2330       2340       2350
SGSSQSSSYG QQGSGSGQSP SRGRHGSGSG HSSSYGQHGS GSGWSSSSGR HGSGSGQSSG FGHHESSSWQ
         G QQGSGSGQSP SRGRHGSGSG HSSSYGQHGS GSGWSSSSGR
         G QQGSGSGQSP SRGRHGSGSG HSSSYGQHGS GSGWS
              SGSGQSP SRGRHGSGSG HSSSYGQHGS GSGWSSSSGR
              SGSGQSP SRGRHGSGSG HSSSYGQHGS GSGWSS
                   SP SRGRHGSGSG HSSSYGQHGS GSGWS
                       RGRHGSGSG HSSSYGQHGS GSGWS
                        GRHGSGSG HSSSYGQHGS GSGWSSSSGR HGSGSGQSSG FGHHES
                        GRHGSGSG HSSSYGQHGS GSGWSSSSGR HGSGSGQSSG FGHHE
                        GRHGSGSG HSSSYGQHGS GSGWSSSSGR HG
                        GRHGSGSG HSSSYGQHGS GSGWSSSSGR
                        GRHGSGSG HSSSYGQHGS GSGWS
                        GRHGSGSG HSSSYGQHGS GSGW
                          HGSGSG HSSSYGQHGS GSGWSSSSGR HGSGSGQSSG FGHHESS
                          HGSGSG HSSSYGQHGS GSGWSSSSGR HGSGSGQSSG FGHHES
                          HGSGSG HSSSYGQHGS GSGWSSSSGR HGSGSGQSSG FGHHE
                          HGSGSG HSSSYGQHGS GSGW
                                      GQHGS GSGWSSSSGR HGSGSGQSSG FGHHESS
                                      GQHGS GSGWSSSSGR HGSGSGQSSG FGHHES
                                      GQHGS GSGWSSSSGR HGSGSGQSSG FGHHE
                                      GQHGS GSGWSSSSGR HGSGSGQSSG FGHH
                                         GS GSGWSSSSGR HGSGSGQSSG FGHHE
                                                SSSSGR HGSGSGQSSG FGHHESSSWQ
                                                SSSSGR HGSGSGQSSG FGHHESSSW
                                                   SGR HGSGSGQSSG FGHHESSSW
                                                    GR HGSGSGQSSG FGHHESSSWQ
                                                       HGSGSGQSSG FGHHESSSW

      2380       2390       2400       2410       2420       2430       2440       2450       2460       2470       2480       2490
GSRSGQSSRG ERHGSSSGSS SSYGQHGSGS RQSLGHGQHG SGSGQSPSPS RGRHGSGSGQ SSSYSPYGSG SGWSSSRGPY ESGSGHSSGL GHRESRSGQS SGYGQHGSSS GHSSTHGQHG
         G ERHGSSSGSS SSYGQHGSGS RQSLGHGQHG SGSGQSPSPS                                   RGPY ESGSGHSSGL GHRESR
             HGSSSGSS SSYGQHGSGS RQSLGHGQHG SGSGQSPSPS                                        ESGSGHSSGL GHRESRSGQS SGYGQHGSSS GHSSTHGQHG
             HGSSSGSS SSYGQHGSGS RQSL
                                  QSLGHGQHG SGSGQSPSPS RGRHGSGSGQ SSSY
                                     GHGQHG SGSGQSPSPS RGRHGSGSGQ SSSYSPY

      2550       2560       2570       2580       2590       2600       2610
RGSGSRQSPS YGRHGSGSGR SSSSGQHGSG LGESSGFGHH ESSSGQSSSY SQHGSGSGHS SGYGQHGSRS
      QSPS YGRHGSGSGR SSSSGQHGSG LGESSGFGHH ESSSGQSSS
      QSPS YGRHGSGSGR SSSSGQHGSG LGESSGFGHH E
      QSPS YGRHGSGSGR SSSSGQHGSG LGE
            GRHGSGSGR SSSSGQHGSG LGESSGFGHH ESSSGQSSSY
            GRHGSGSGR SSSSGQHGSG LGESSGFGHH ESSSGQSSS
            GRHGSGSGR SSSSGQHGSG LGESSGFGHH ESSSGQSS
            GRHGSGSGR SSSSGQHGSG LGESSGFGHH ESSSGQS
            GRHGSGSGR SSSSGQHGSG LGESSGFGHH ESSSG
            GRHGSGSGR SSSSGQHGSG LGESSGFGHH ESS
            GRHGSGSGR SSSSGQHGSG LGESSGFGHH ES
            GRHGSGSGR SSSSGQHGSG LGESSGFGHH E
             RHGSGSGR SSSSGQHGSG LGESSGFGHH ESSSGQSSS
              HGSGSGR SSSSGQHGSG LGESSGFGHH ESSSGQSSS
               GSGSGR SSSSGQHGSG LGESSGFGHH ESSSGQSSS
                      SSSSGQHGSG LGESSGFGHH ESSSGQSSSY SQHGSGSGHS SGYGQHGS
                      SSSSGQHGSG LGESSGFGHH ESSSGQSSSY
                      SSSSGQHGSG LGESSGFGHH ESSSGQSSS
                      SSSSGQHGSG LGESSGFGHH ESSSGQSS
                      SSSSGQHGSG LGESSGFGHH ESSSGQS
                      SSSSGQHGSG LGESSGFGHH ESSSGQ
                      SSSSGQHGSG LGESSGFGHH ESSSG
                      SSSSGQHGSG LGESSGFGHH ESSS
                      SSSSGQHGSG LGESSGFGHH ESS
                      SSSSGQHGSG LGESSGFGHH ES
                      SSSSGQHGSG LGESSGFGHH E
                      SSSSGQHGSG LGESSGFGHH
                        SSGQHGSG LGESSGFGHH ESSSGQSSS
                         SGQHGSG LGESSGFGHH ESSSGQSSS
                          GQHGSG LGESSGFGHH ESSSGQSSS
                            HGSG LGESSGFGHH ESSSGQSSS

      2660       2670       2680       2690       2700
QSPSRGRHGS GLGHSSSHGQ HGSGSGRSSS RGPYESRLGH SSVFGQHESG
     GRHGS GLGHSSSHGQ HGSGSGRSSS RGPYE
     GRHGS GLGHSSSHGQ HGSGSGRSSS RGPY
       HGS GLGHSSSHGQ HGSGSGRSSS RGPYE
       HGS GLGHSSSHGQ HGSGSGRSSS RGPY
        GS GLGHSSSHGQ HGSGSGRSSS RGPY
           GLGHSSSHGQ HGSGSGRSSS RGPY
```

Supplementary Figure 1 I Complete list of all cationic HRNR-peptides identified in stratum corneum.

Supplementary Figure 2

Amino acid	P4	P3	P2	P1	P1'	P2'	P3'	P4'		
A	0	1	1	1	0	1	1	0	A	40
C	0	0	0	0	0	0	0	0	C	
D	0	0	0	0	0	0	0	0	D	
E	2	4	2	3	3	3	0	0	E	
F	1	1	1	1	0	0	0	2	F	
G	20	17	21	9	17	21	23	28	G	
H	7	9	10	6	7	6	11	12	H	
I	0	0	0	0	0	0	0	0	I	
K	0	0	0	0	0	2	1	0	K	
L	1	1	0	2	0	0	0	0	L	
M	0	0	0	0	0	0	0	0	M	
N	0	0	0	0	1	1	1	1	N	
P	1	3	5	1	0	1	3	1	P	
Q	9	5	5	4	6	9	10	9	Q	
R	8	6	1	17	8	5	7	4	R	
S	31	38	39	29	40	38	31	30	S	
T	2	2	0	1	1	0	0	0	T	
V	1	0	1	0	1	2	0	0	V	
W	3	2	2	3	1	1	1	1	W	
Y	5	2	3	14	6	1	2	3	Y	0

Amino acid	P4	P3	P2	P1	P1'	P2'	P3'	P4'		
A	0.00	0.04	0.04	0.04	0.00	0.04	0.04	0.00	A	0.25
C	0.00	0.00	0.00	0.00	0.00	0.00	0.00	0.00	C	
D	0.00	0.00	0.00	0.00	0.00	0.00	0.00	0.00	D	
E	0.03	0.05	0.03	0.04	0.04	0.04	0.00	0.00	E	
F	0.03	0.03	0.03	0.03	0.00	0.00	0.00	0.05	F	
G	0.03	0.02	0.03	0.01	0.02	0.03	0.03	0.04	G	
H	0.03	0.03	0.04	0.02	0.03	0.02	0.04	0.04	H	
I	0.00	0.00	0.00	0.00	0.00	0.00	0.00	0.00	I	
K	0.00	0.00	0.00	0.00	0.00	0.11	0.05	0.00	K	
L	0.03	0.03	0.00	0.05	0.00	0.00	0.00	0.00	L	
M	0.00	0.00	0.00	0.00	0.00	0.00	0.00	0.00	M	
N	0.00	0.00	0.00	0.00	0.07	0.07	0.07	0.07	N	
P	0.02	0.05	0.09	0.02	0.00	0.02	0.05	0.02	P	
Q	0.03	0.02	0.02	0.01	0.02	0.03	0.04	0.03	Q	
R	0.05	0.04	0.01	0.10	0.05	0.03	0.04	0.02	R	
S	0.03	0.04	0.04	0.03	0.04	0.04	0.03	0.03	S	
T	0.05	0.05	0.00	0.02	0.02	0.00	0.00	0.00	T	
V	0.06	0.00	0.06	0.00	0.06	0.12	0.00	0.00	V	
W	0.25	0.17	0.17	0.25	0.08	0.08	0.08	0.08	W	
Y	0.05	0.02	0.03	0.13	0.06	0.01	0.02	0.03	Y	0.00

Supplementary Figure 2 I Analysis of the sequences surrounding the cleavage sites (P1 position) of all HRNR-peptides identified in stratum corneum. The heat maps show (left panel) the number of residues identified in position P4-P1 and P1'-P4', respectively, and (right panel) the values of these residues normalized vs. the total number of the particular amino acid in the HRNR canonical sequence. Annotation of P4-P1 and P1'-P4' according to ref.[93].

Supplementary Figure 3

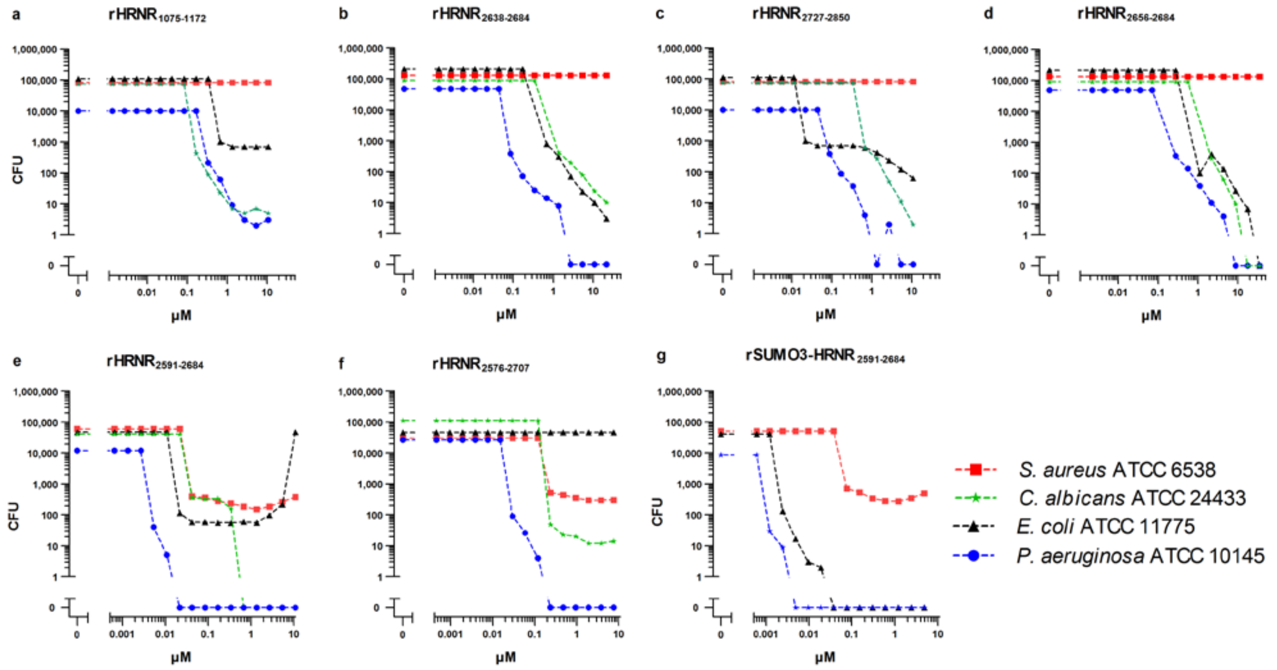

Supplementary Figure 3 I HRNR fragments are potent broad-spectrum antimicrobial polypeptides. Sensitivity of *P. aeruginosa* ATCC 10145, *E. coli* ATCC 11775, *S. aureus* ATCC 6538 and *C. albicans* ATCC 24433 towards **a**, rHRNR$_{1075-1172}$; **b**, rHRNR$_{2638-2684}$; **c**, rHRNR$_{2727-2850}$; **d**, rHRNR$_{2656-2684}$; **e**, rHRNR$_{2591-2684}$; **f**, rHRNR$_{2576-2707}$; and **g**, rSUMO3-HRNR$_{2591-2684}$ has been tested in a CFU assay system at pH 5.5 in 10 mM NaP/1% TSB (**a-d**) or in 10 mM NaP/0.25% glucose (**e-g**). Note *E. coli* ATCC 11775 missing sensitivity towards rHRNR$_{2576-2707}$ at these conditions (**f**), and an "antibiotic paradox" (decrease of sensitivity at increasing peptide concentrations) towards rHRNR$_{2591-2684}$ (**e**). AA-sequences of the HRNR-polypeptides are shown in (Supplementary Table 1). Results shown are representatives (n=2) (**a-d, f, g**) and (n=3) (**e**).

Supplementary Figure 4

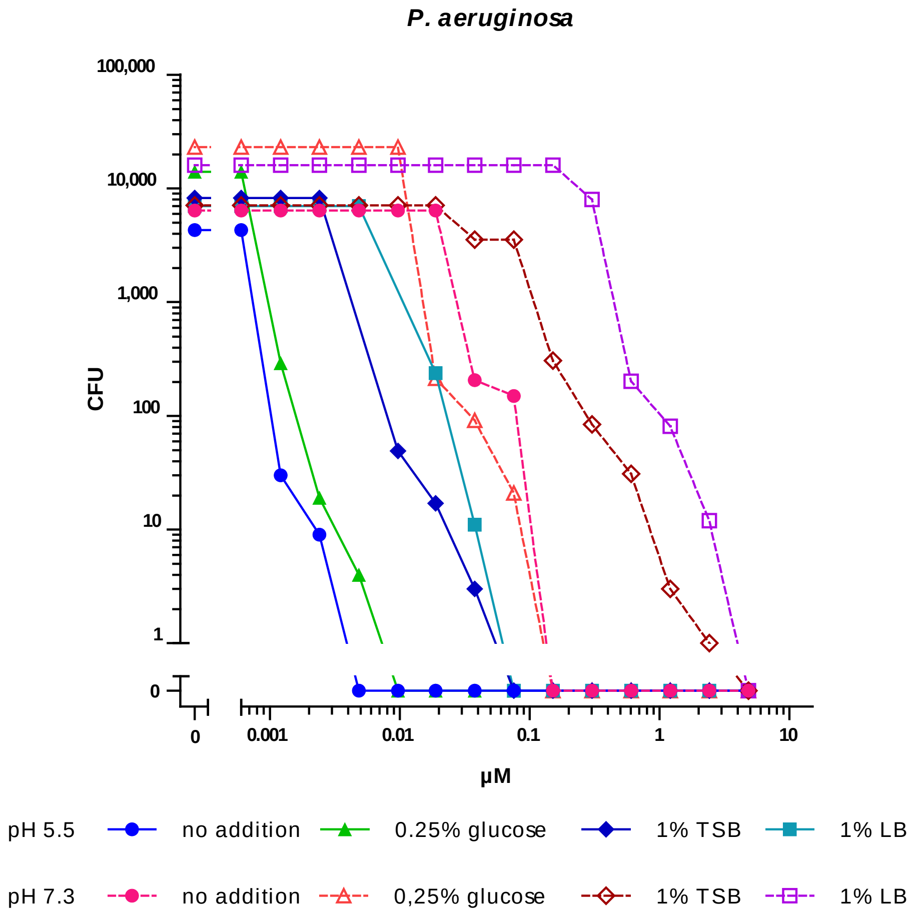

Supplementary Figure 4 I *P. aeruginosa*-cidal activity of SUMO3-HRNR$_{2591\text{-}2684}$ is pH-dependent and is inhibited by "Tryptic Soybean Broth, TSB" and "Luria Broth, LB". Recombinant SUMO3-HRNR$_{2591\text{-}2684}$ was tested at the indicated conditions in 10 mM NaP/0.25% glucose or 1% TSB, 1% LB or no addition. Note a shift of the dose-response curve by both, increased pH and addition of nutrients like TSB or LB. All data shown are representatives (n=2).

Supplementary Figure 5:

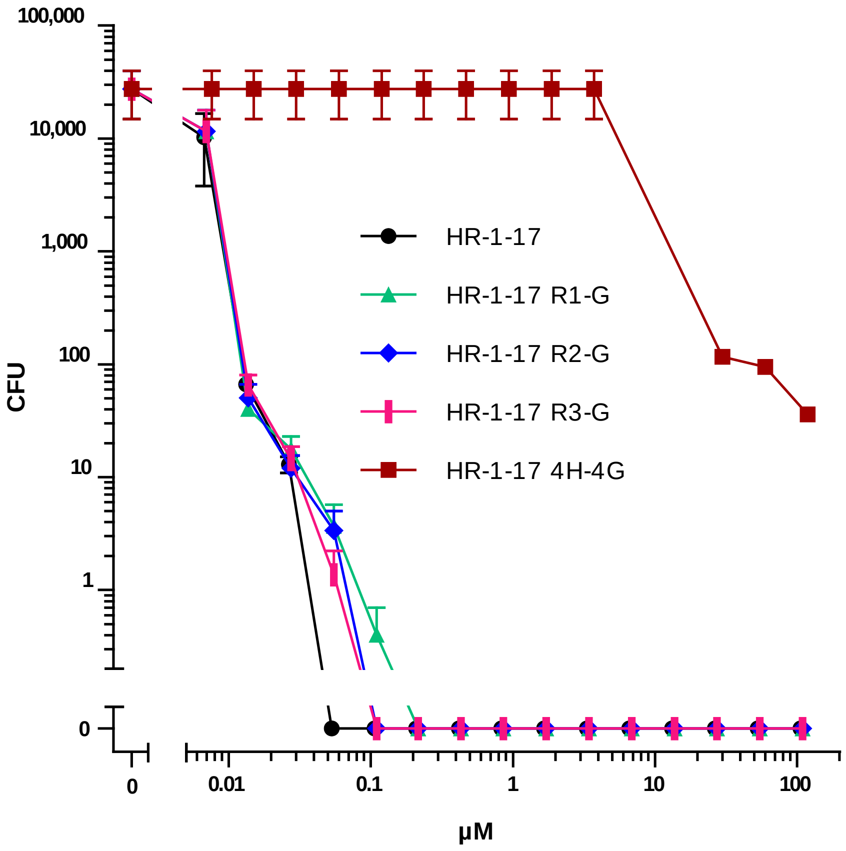

Supplementary Figure 5 I *P. aeruginosa* ATCC 10145-cidal activity of HR 1-17 mutants. *Pseudomonas aeruginosa* ATCC 10145-cidal activity in 10 mM NaP/0.25% glucose/pH 5.5, was examined for four mutants of HR1-17 with replacement of either Arg or His by Gly (AA-sequences are shown in Supplementary Table 4). Data represent the mean ± s.e.m. (n=3).

Supplementary Figure 6:

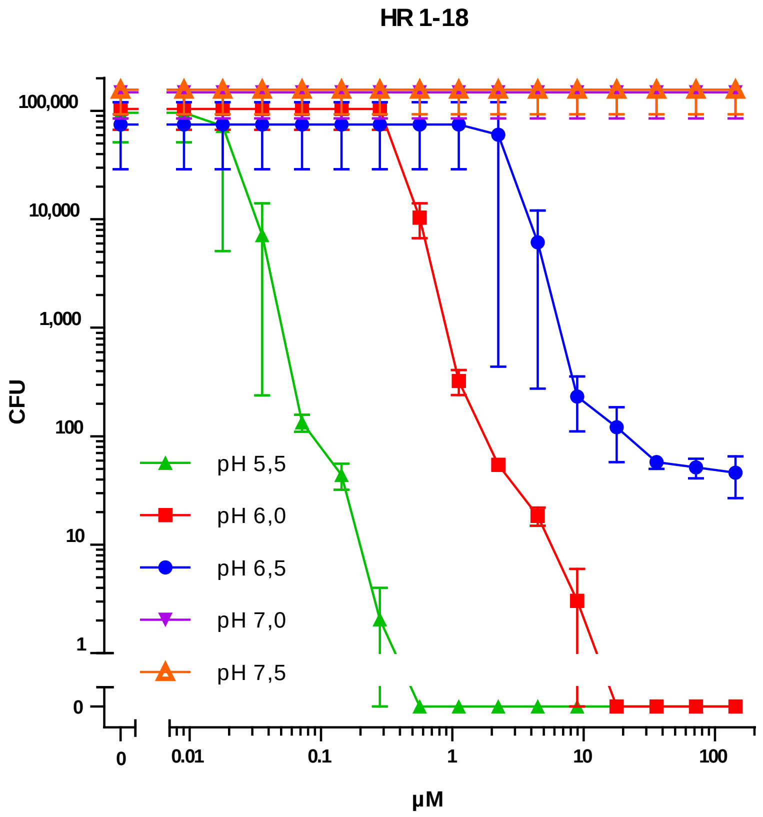

Supplementary Figure 6 | *P. aeruginosa*-cidal activity of HR1-18 depends on pH. *P. aeruginosa* ATCC 10145-cidal activity of HR1-18 was examined in 10 mM NaP/ 0.25% glucose/pH 5.5-7.5. Data shown represent the mean ± s.e.m. (n=3).

Supplementary Figure 7:

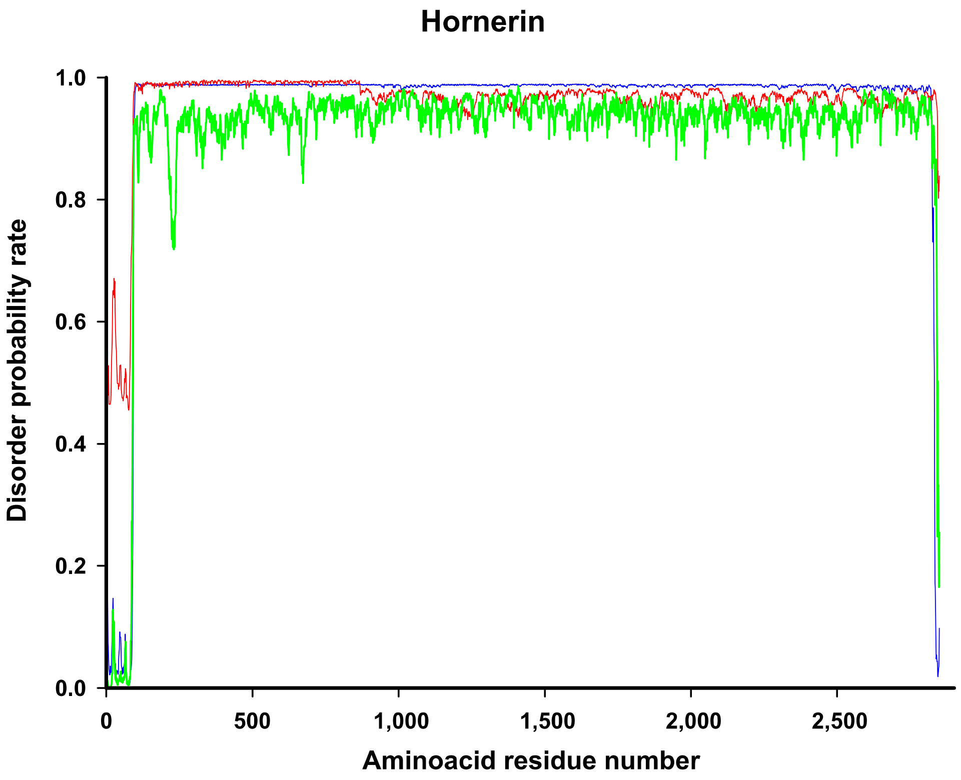

Supplementary Figure 7 I Evaluation of the intrinsic disorder predisposition in HRNR by three computational tools. The amino acid position (abscissa) is shown as a function of the disorder probability rate. Results of the PreDisorder program[94] are given in blue, of the DisoClust program[95] given in red and results of the DISOPRED2 program[96] appear in green. There is a clear difference between the N-terminal ordered EF-hand-like-domain (AA 1-100), the area of repetitive Gly/Ser-rich units detected as a large intrinsically disordered region and the small C-terminal ordered region.

Supplementary Figure 8:

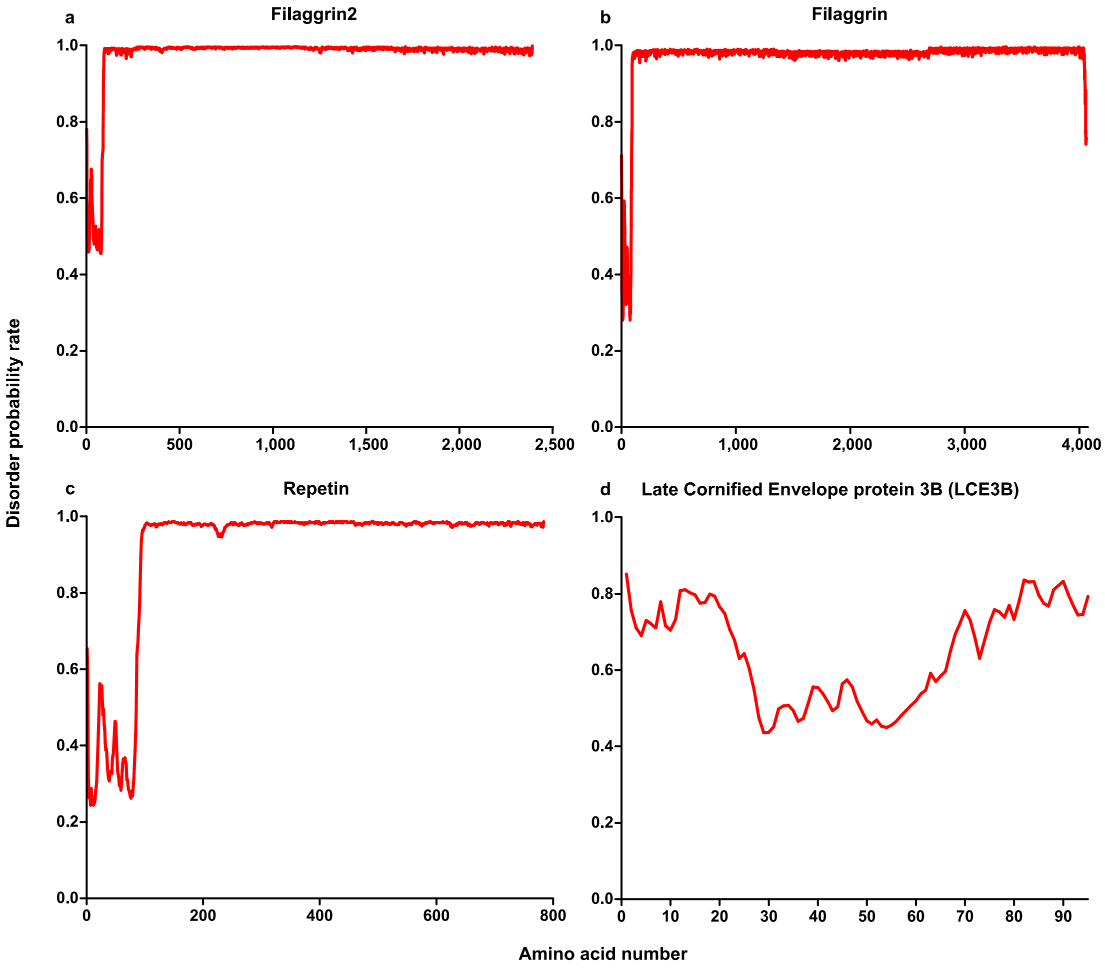

Supplementary Figure 8 I Evaluation of the intrinsic disorder predisposition FLG-2, FLG, RPT and LCE3B by the DisoClust program. a: FLG-2; b: FLG; c: RPT; d: LCE3B. The amino acid position (abscissa) is shown as a function of the disorder probability rate. Results of the DisoClust program[95] are given. In the three "S100-fused-type-proteins FLG2, FLG and RPT there is a clear difference between the N-terminal ordered EF-hand-like-domain (AA 1-100) and the area rich in repetitive disorder promoting AA. LCE3B reveals, possibly due to its high content of Cys and His, a disorder probability between 50 - 70 % in the relevant stretch of AA 56 to AA 78 in $LCE3B_{56\text{-}68}$. Notably, this peptide is investigated for antimicrobial activity in its thiol-form at pH 5.5, where His is protonated. This is not taken into account by the DisoClust program and may further increase the disorder probability rate.

Supplementary Figure 9:

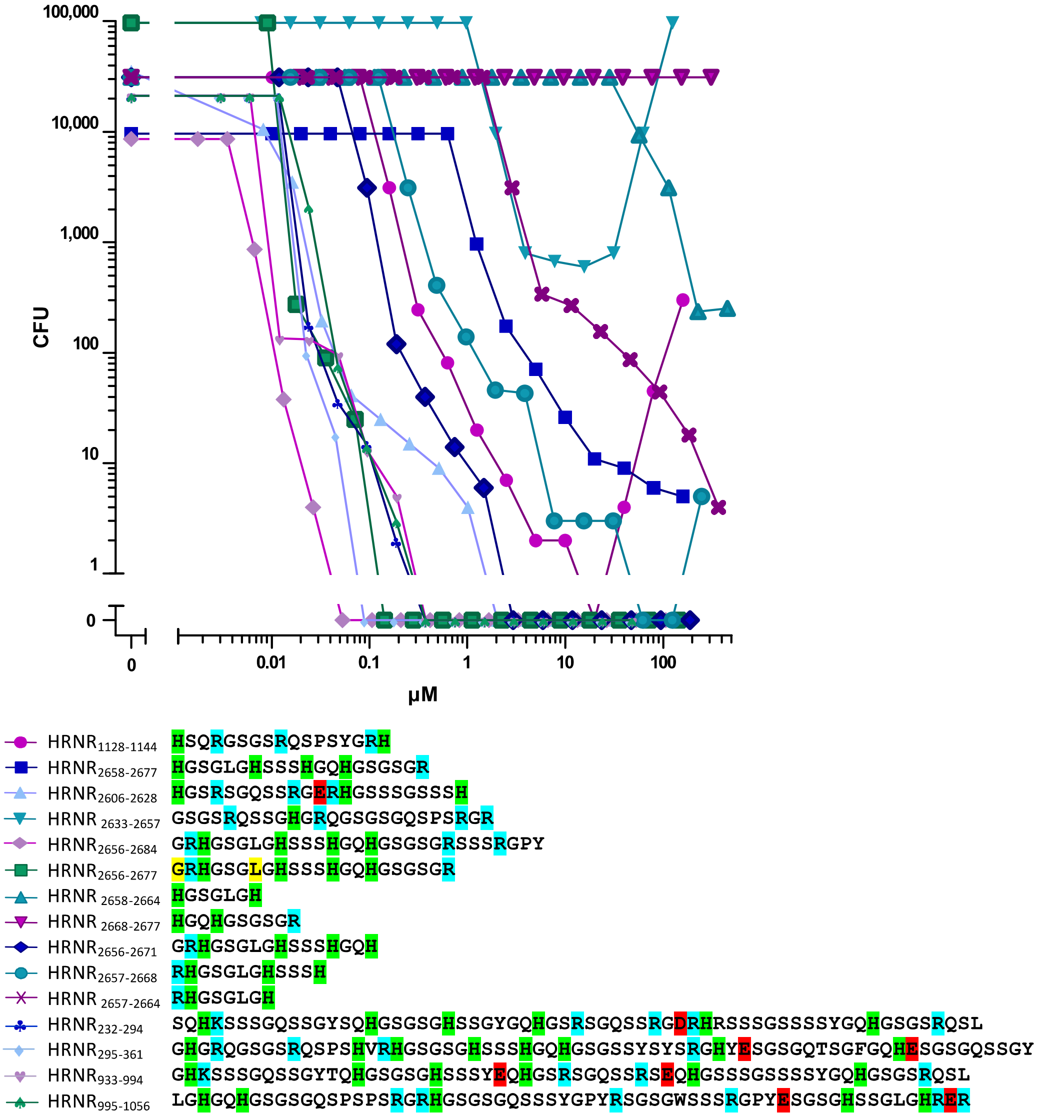

Supplementary Figure 9 I *P. aeruginosa*-cidal activity of selected HRNR peptides. Sensitivity of *P. aeruginosa* ATCC 10145 towards various peptides was tested in 10 mM NaP/0.25% glucose/pH 5.5. In the peptide's amino acid sequences residues cationic at pH 7, cationic only at pH 5.5 and anionic at pH 5.5 are colored blue, green and red, respectively. Antimicrobial paradox[16] (increased bacterial growth with increasing CIDAMP-concentrations) was observed for $HRNR_{1128-1144}$ and $HRNR_{2633-2657}$. Representative dose-response curves of selected HRNR-peptides are shown (for the number of experiments see Supplementary Table 6).

Supplementary Figure 10:

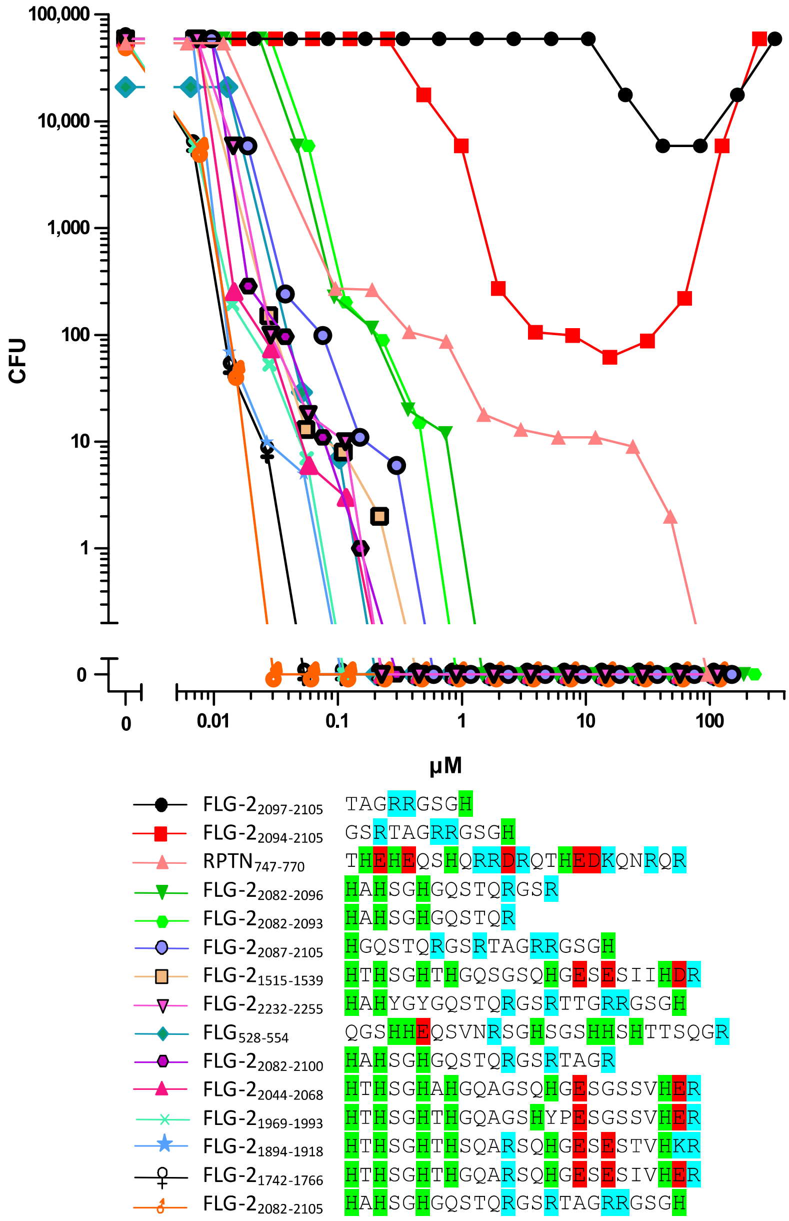

Supplementary Figure 10 I *P. aeruginosa*-cidal activity of selected FLG-2-, FLG- and RPTN-derived CIDAMPs. Sensitivity of *P. aeruginosa* ATCC 10145 was tested in 10 mM NaP/ 0.25% glucose/pH 5.5. Residues cationic at pH 7 (R, K), cationic only at acidic pH (H) and anionic (E, D) are colored blue, green and red, respectively. Antimicrobial paradox[16] (increased bacterial growth with increasing CIDAMP-concentrations) was observed for FLG-$2_{2094\text{-}2105}$ and FLG-$2_{2097\text{-}2105}$. Representative dose-response curves of selected FLG-2-; FLG- and RPTN-peptides are shown (for the number of experiments see Supplementary Table 7).

Supplementary Figure 11:

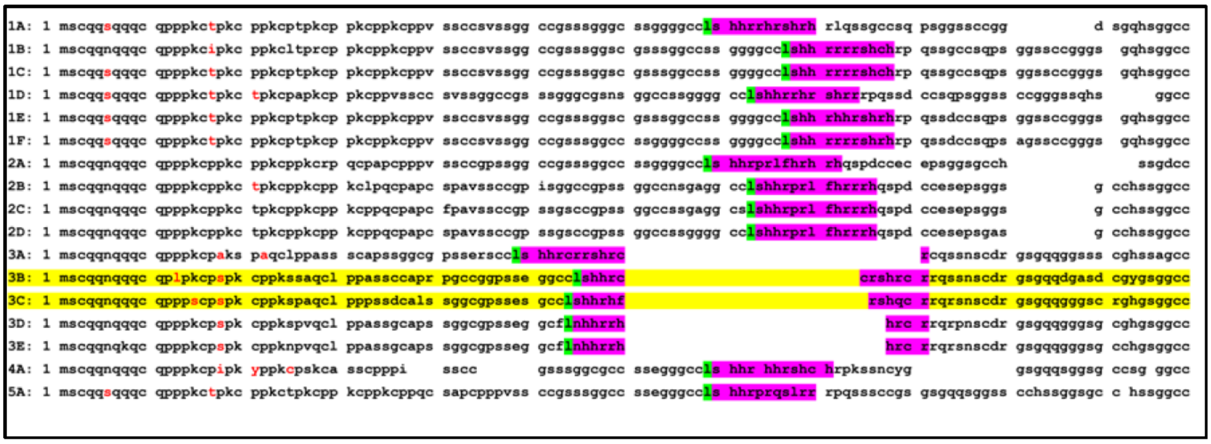

Supplementary Figure 11 I Amino acid sequences of human Late-Cornified-Envelope proteins (LCEs). AA-sequences of LCEs (1A – 5A) are shown and LCEs, investigated in this study, are highlighted in yellow. Note the conserved, potential cathepsin D cleavage sites (P1: Leu, highlighted in green) in all LCEs. Antimicrobially active LCE-peptide fragments in LCE3B and LCE3C as well as putatively antimicrobially active fragments of other LCEs are highlighted in magenta.

Supplementary Figure 12:

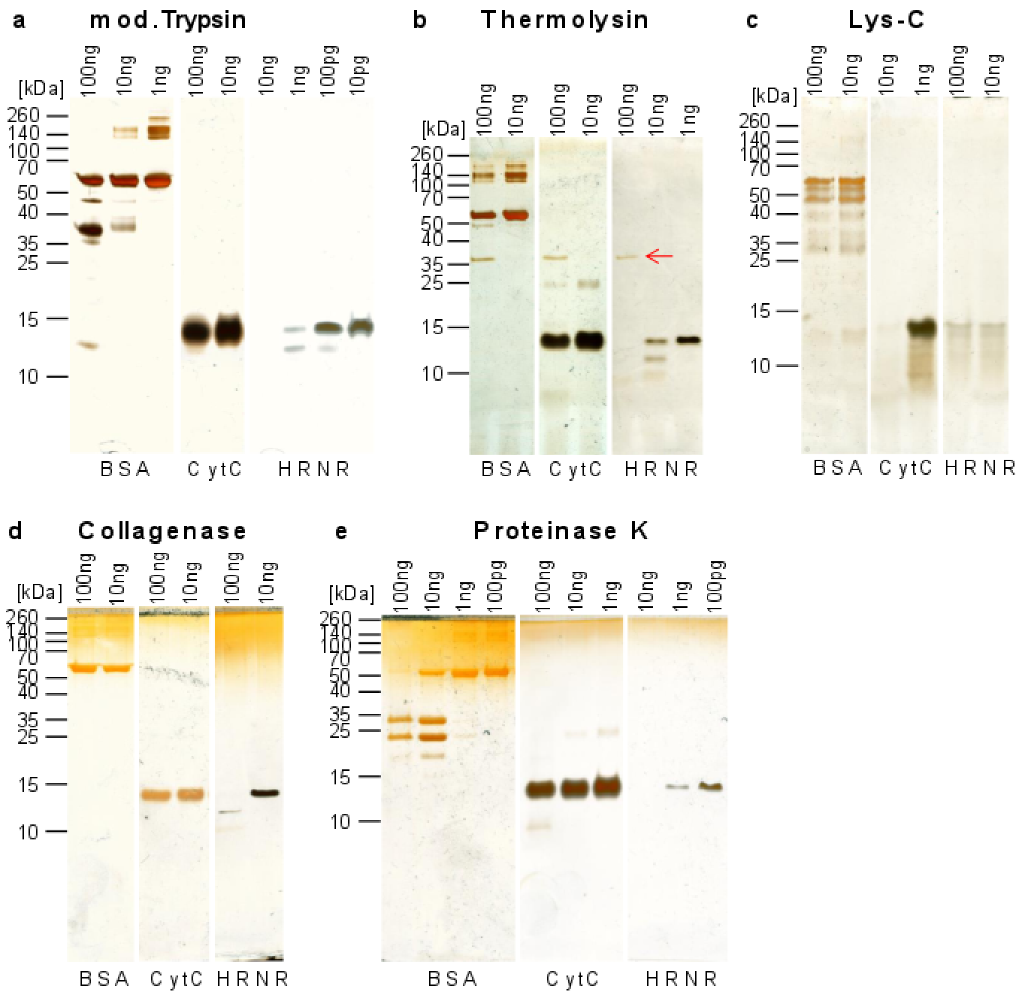

Supplementary Figure 12 I Protease sensitivity of rHRNR$_{2591-2684}$, BSA and cytochrome C. 1 µg each of bovine serum albumin (BSA), cytochrome (Cyt) C or rHRNR$_{2591-2684}$ (HRNR, a different HRNR-fragment as used in Fig. 8) were incubated with the proteases indicated above in protease-specific buffers for 1 h at 37 °C. Thereafter samples were subjected to a SDS-PAGE analysis and proteins were detected by silver staining. **a**, modified (methylated) serine proteinase trypsin; **b**, the metalloproteinase thermolysin (red arrow); **c**, the serine proteinase Lys C; **d**, the metalloproteinase collagenase; and **e**, proteinase K. Note the sensitivity of rHRNR$_{2591-2684}$ towards digestion by Lys C and collagenase, albeit higher concentrations, although predicted cleavage sites for these enzymes are absent in rHRNR$_{2591-2684}$ (for AA-sequence see Supplementary Table 1). Data shown are representatives (n=3).

Supplementary Figure 13:

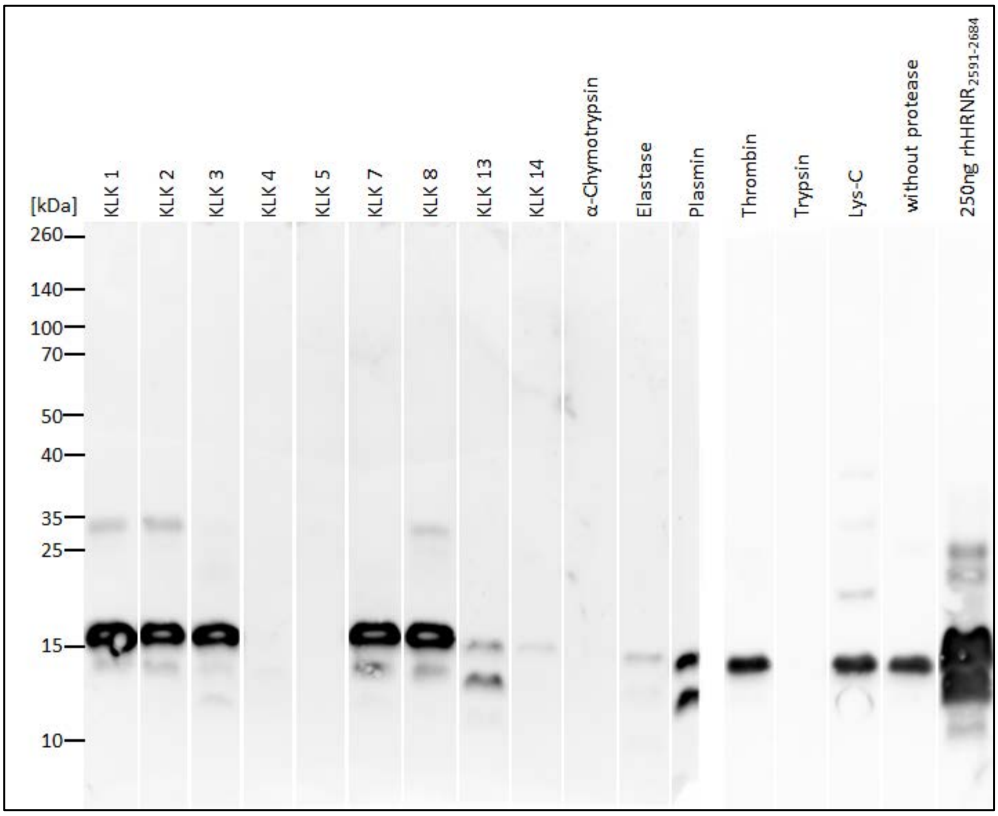

q

Supplementary Figure 13 I KLK4, KLK5, KLK13, KLK14 and trypsin, but not KLK1, KLK7, KLK8 and LysC digest rHRNR$_{2591\text{-}2684}$. 500 ng rHRNR$_{2591\text{-}2684}$ each were incubated with 0.7 nM of the respective, activity-checked protease in buffer (100 mM Tris, pH 7.5, 150 mM NaCl, 5 mM EDTA, 0.05 Tween 20) for 15 h. For SDS-PAGE analyses 250 ng of rHRNR$_{2591\text{-}2684}$ were used and then analyzed by rHRNR$_{2591\text{-}2684}$-Westernblot. Lane 16 (“without protease”) contains a control sample treated in the same manner, however in the absence of a protease. In lane 17, 250 ng of an rHRNR$_{2591\text{-}2684}$-pool (for AA-sequence see Supplementary Table 1) have been directly applied. Note the presence of multiple bands, which originate from oligomeric HRNR-complexes[12]. Data shown are representatives (n=3).

Supplementary Figure 14:

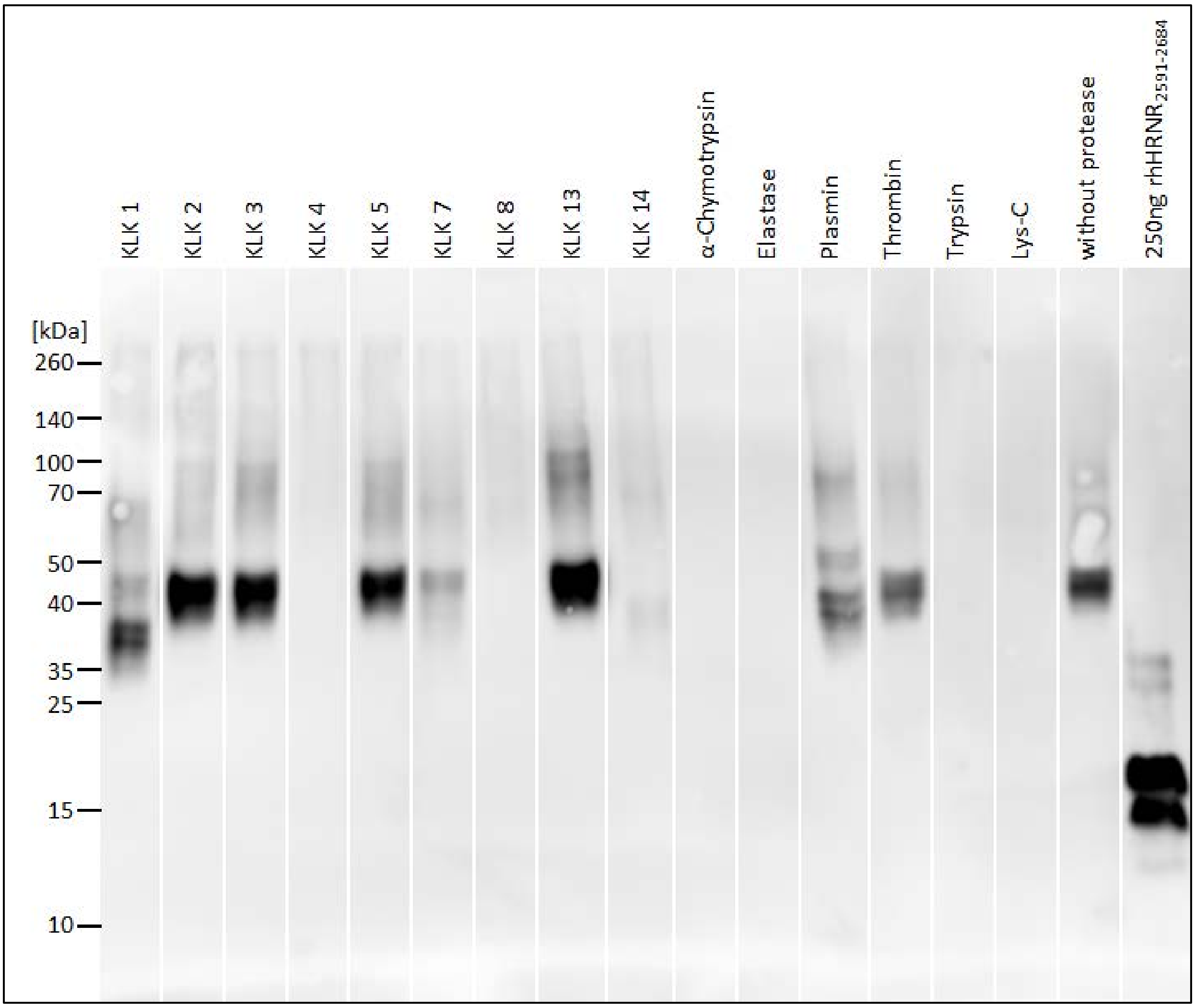

Supplementary Figure 14 I KLK1, KLK4, KLK7, KLK8, KLK14, trypsin and LysC, but not KLK13 digest PAD1-deiminated rHRNR$_{2591-2684}$. 500 ng PAD1-deiminated rHRNR$_{2591-2684}$ each were incubated for 15 h with 0.7 nM of the respective, activity-tested protease in buffer (100 mM Tris pH 7.5, 150 mM NaCl, 5 mM EDTA, 0.05% Tween 20). For SDS-PAGE analyses 250 ng of the protein were used and then analyzed by rHRNR$_{2591-2684}$-Westernblot. Lane 16 ("without protease") contains a control sample (PAD1-deiminated rHRNR$_{2591-2684}$), treated in the same manner, however in the absence of a protease. In lane 17, 250 ng of an rHRNR$_{2591-2684}$-pool have been directly applied. Note a marked decrease of PAD1-deiminated rHRNR$_{2591-2684}$ electromobility (lane 16) relative to rHRNR$_{2591-2684}$ (lane 17). Data shown are representatives (n=3).

Supplementary Figure 15:

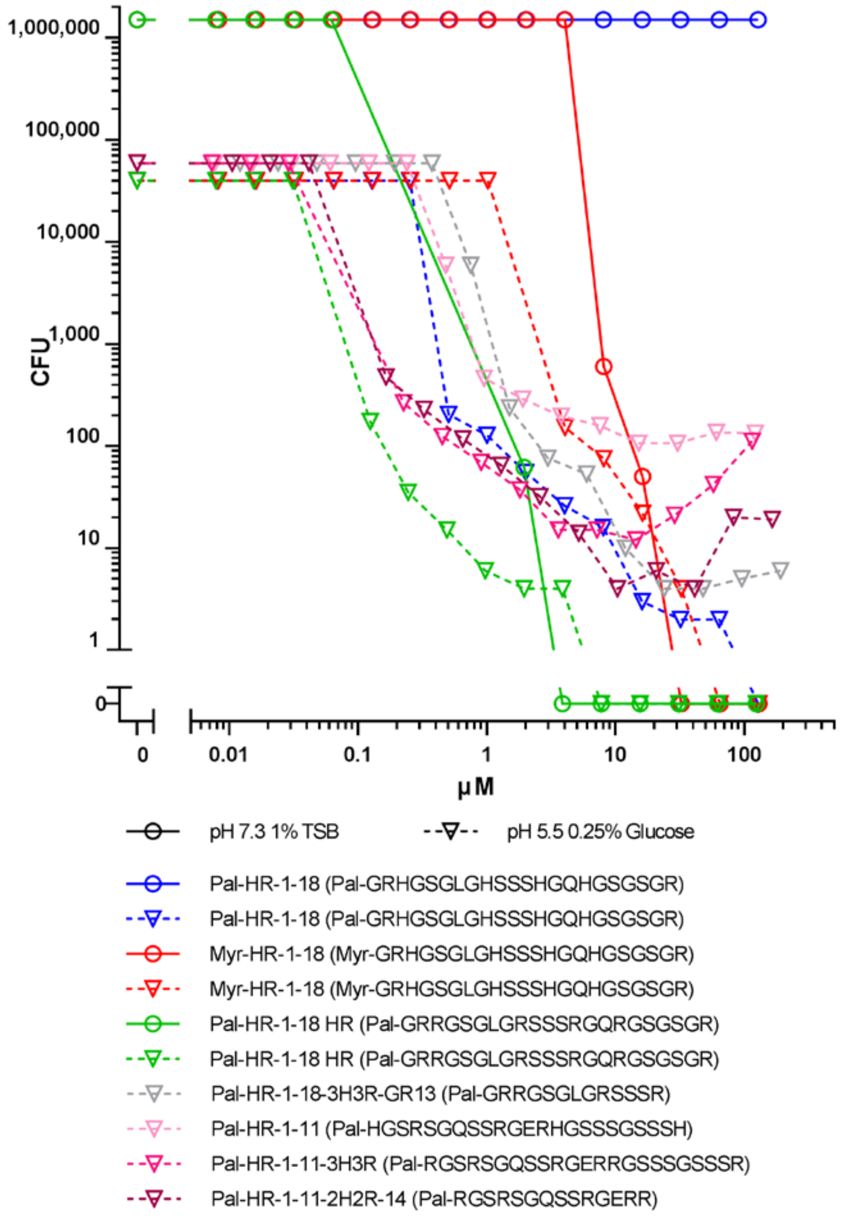

Supplementary Figure 15 I Palmitoylation and myristoylation of CIDAMPs improves *S. aureus*-cidal activity of HR1-18, HR1-11 and its Arg-mutants. *S. aureus* ATCC 6538-cidal activity, assayed either in 10 mM NaP, pH 5.5, containing 0.25% glucose, or in 10 mM NaP, pH 7.3, containing 1 % TSB, was examined in a CFU assay system. Representatives are shown (for the number of experiments see Table 5).

Supplementary Fig. 16

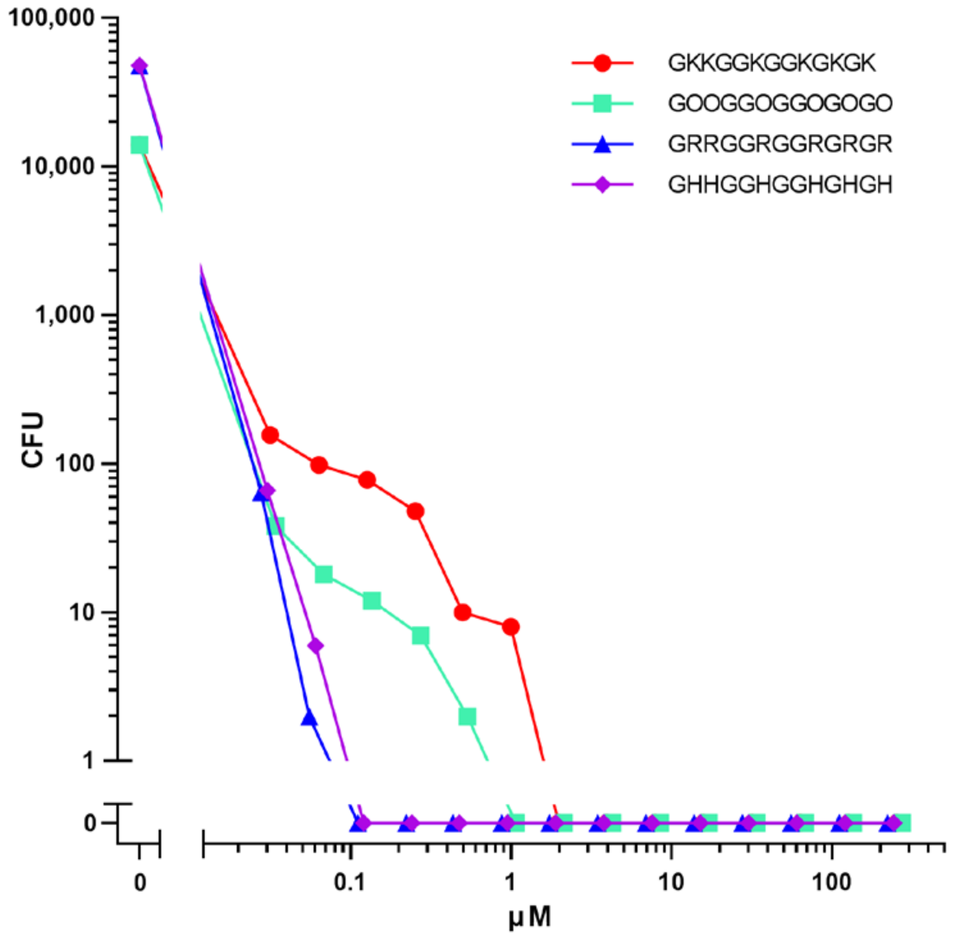

Supplementary Fig. 16 I *P. aeruginosa*-bactericidal activity of Gly-rich 13-mer CIDAMPs containing Lys, Orn, Arg or His. *P. aeruginosa* ATCC 10145-cidal activity was examined in a CFU assay system in 10 mM NaP, pH 5.5, containing 0.25% glucose. Data shown are representatives (n = 2).

Supplementary Figure 17

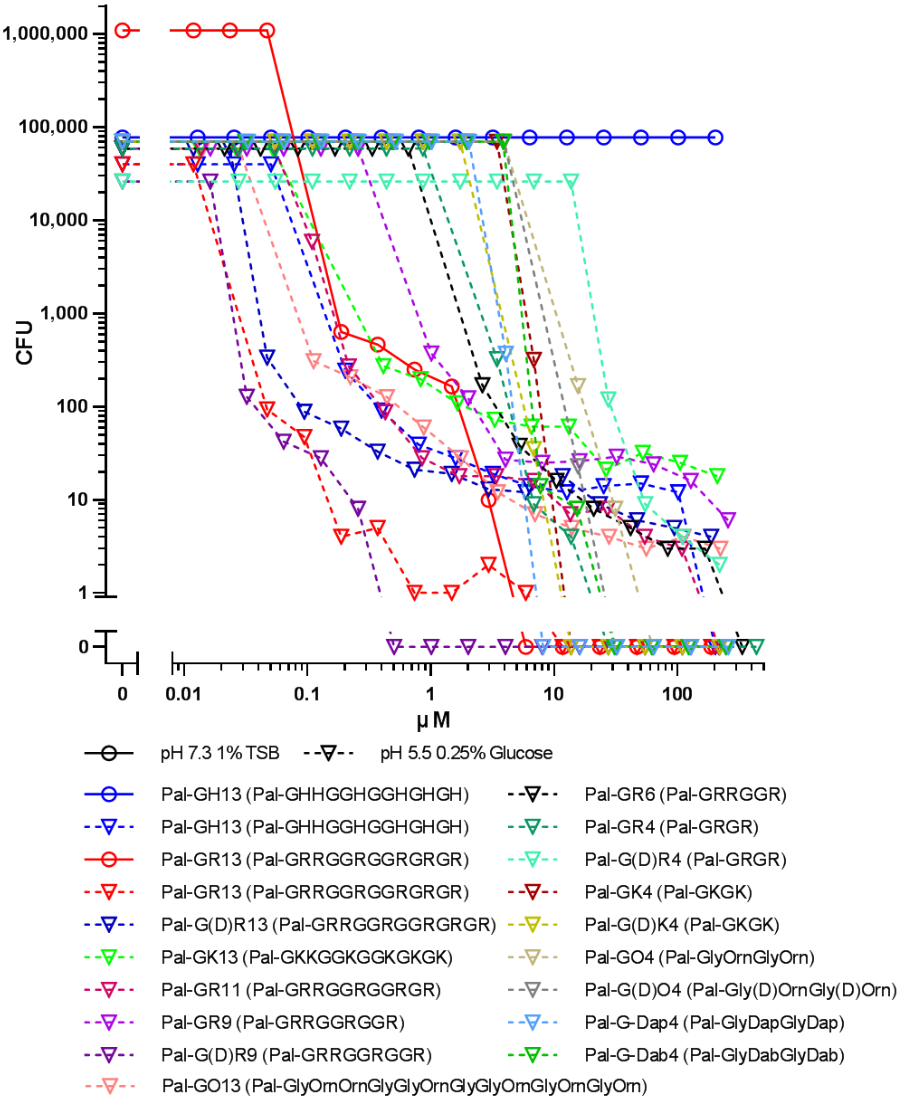

Supplementary Figure 17 I *S. aureus*-cidal activity of palmitoylated CIDAMPs depends on the peptide chain length and positively charged AAs. *S. aureus* ATCC 6538-cidal activity of the lipopeptides was analyzed in a CFU assay system, in 10 mM NaP, pH 5.5, containing 0.25% glucose (solid lines), or in 10 mM NaP, pH 7.3, containing 1 % TSB (dashed lines). Note the loss of sensitivity when Pal-GH13 is tested in TSB-containing medium at pH 7.3. Dab: L-2,4-Diaminobutyric acid, Dap:L-2,3-Diaminopropionic acid. Representatives are shown (for the number of experiments see Table 2).

Supplementary Figure 18

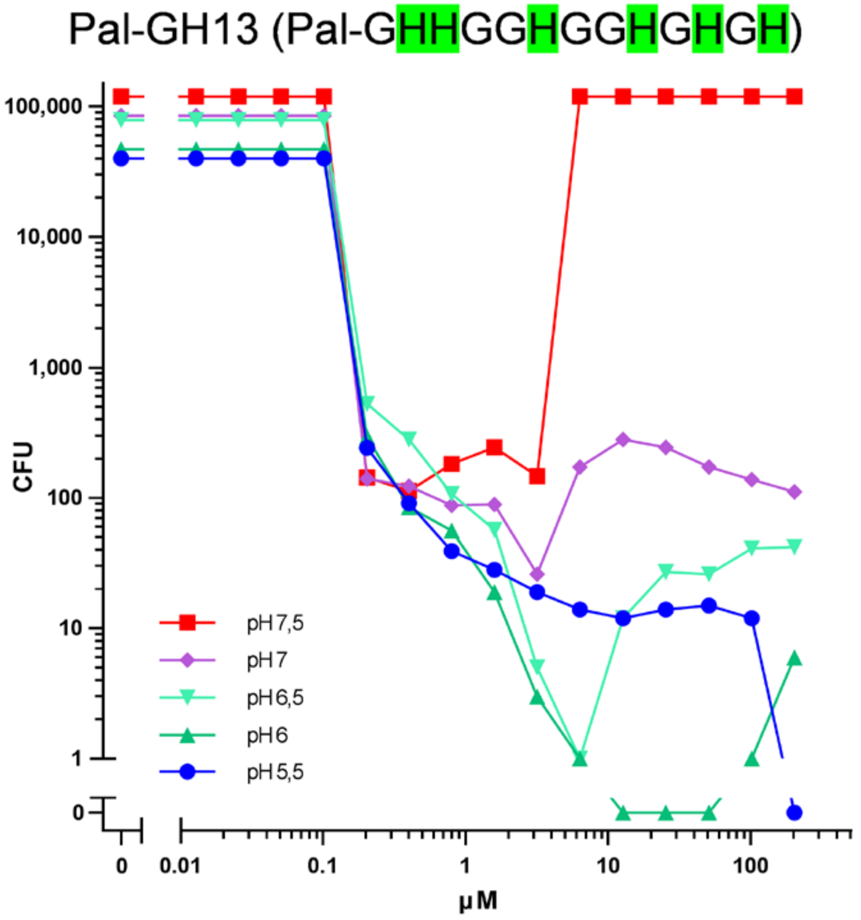

Supplementary Figure 18 I pH-dependency analysis of Pal-GH13-*S. aureus*-cidal activity reveals an "antibiotic paradox". *S. aureus* ATCC 6538-cidal activity of Pal-GH13 was analyzed in a CFU assay system in 10 mM NaP (pH 5.5; 6.0; 6.5; 7.0; 7.5) containing 0.25% glucose. Note that an increase of the pH towards pH 7.5 causes an "antibiotic paradox"[16], which is characterized by an increase of CFU with increasing lipopeptide concentrations. The result of a single experiment is shown.

Supplementary Figure 19

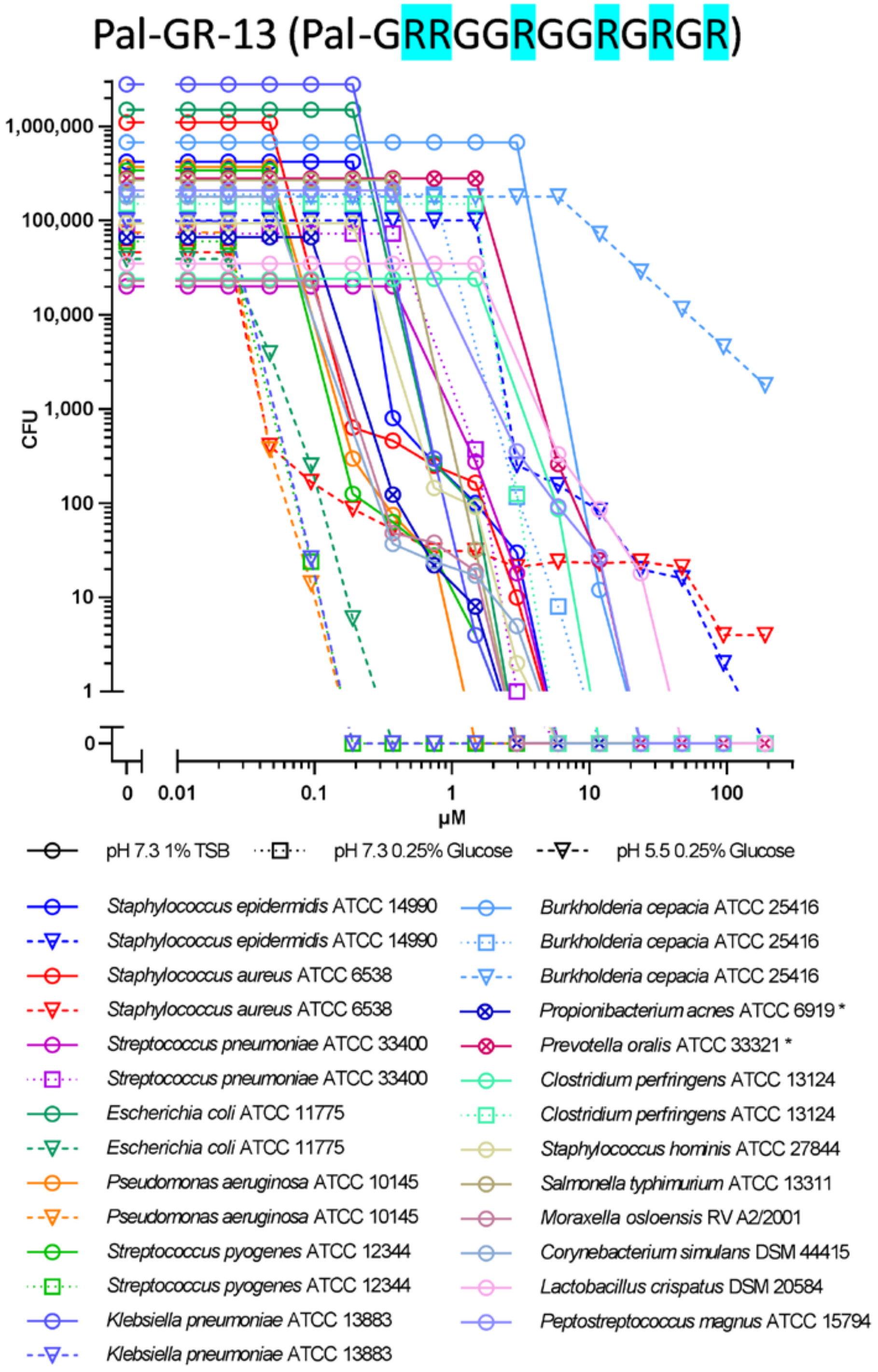

Supplementary Figure 19: Antimicrobial activity spectrum of Pal-GR13. Sensitivity of selected bacteria towards Pal-GR13 was tested in a CFU assay system with 10 mM NaP containing either 0.25% glucose or 1% TSB as test medium, at pH 5.5 or pH 7.3. Representatives are shown (for the number of experiments see Table 7).

Supplementary Figure 20

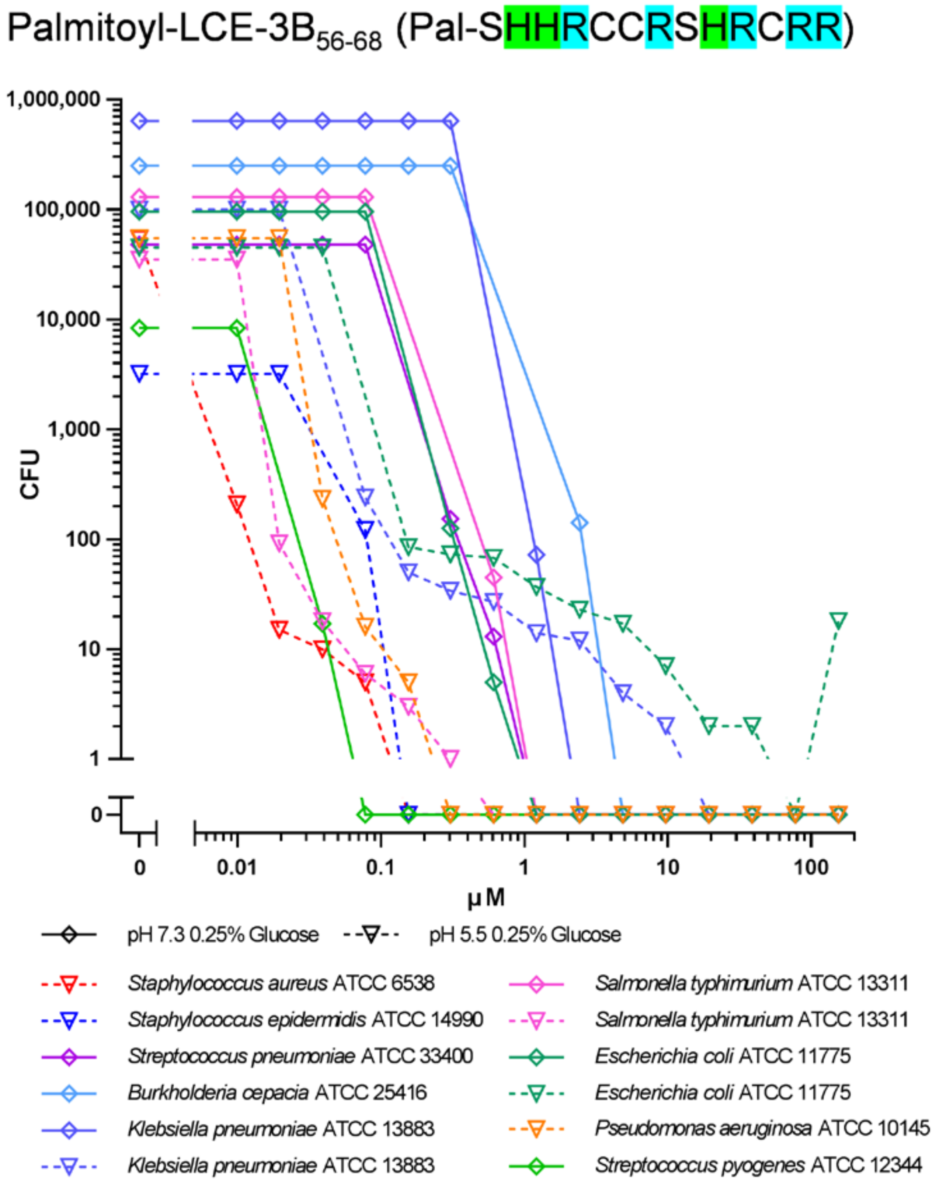

Supplementary Figure 20: Antimicrobial activity spectrum of Pal-LCE-3B$_{56\text{-}68}$. Sensitivity of bacteria towards Pal-LCE-3B$_{56\text{-}68}$ was tested in a CFU assay system in 10 mM NaP, containing 0.25% glucose and, depending on the microbe, at pH 5.5, 6.0 or pH 7.3, as indicated in Supplementary Table 7. Representatives are shown (for the number of experiments see Table 8).

Supplementary Figure 21

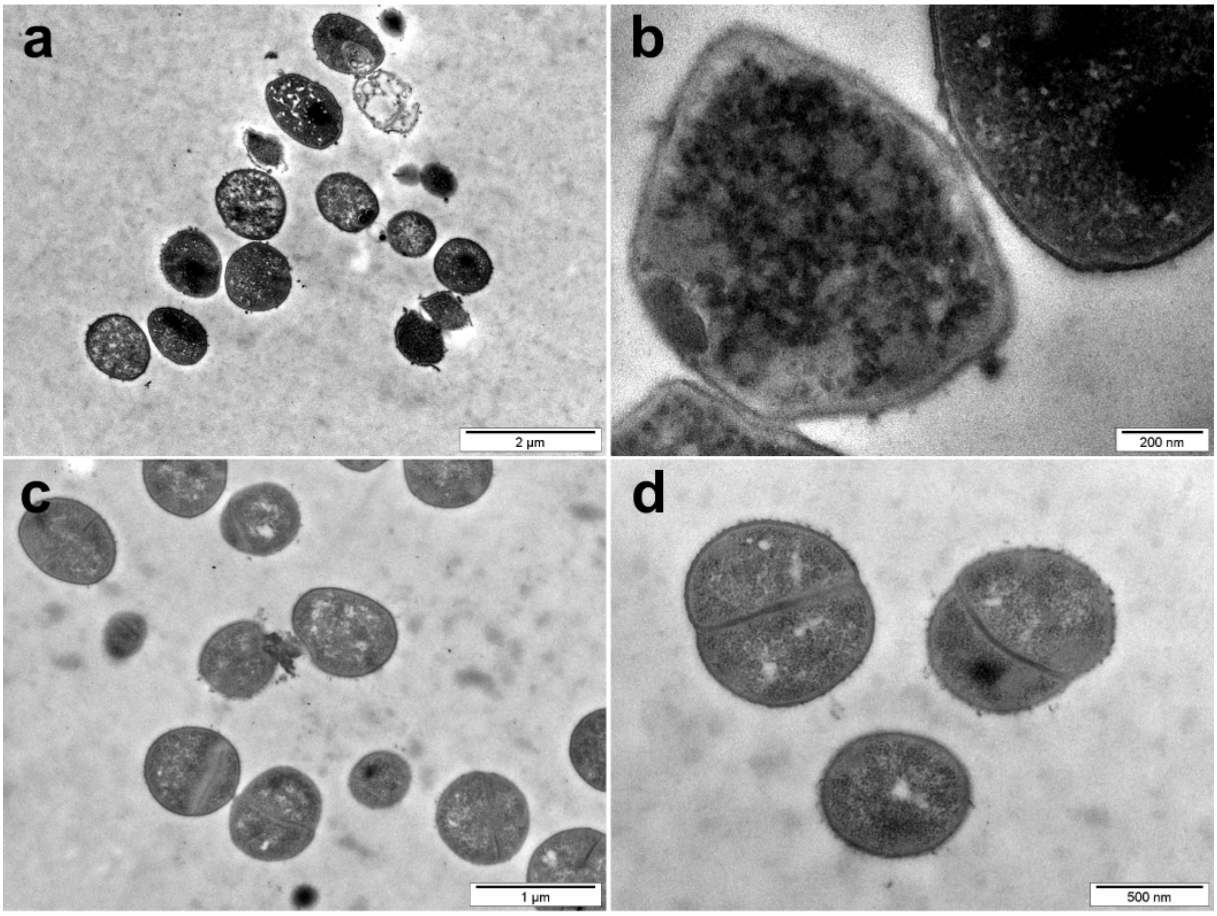

Supplementary Figure 21I TEM of LCE3B$_{56\text{-}68}$-treated *S. aureus*. 10^7 *S. aureus* ATCC 6538, suspended in 150µl 10 mM NaP, pH 5.5, containing 0.25% glucose, were treated with LCE3B$_{56\text{-}68}$ (SHHRCCRSHRCRR, 125 µg/ml) for 90 min at 37°C and then analyzed by transmission electron microscopy (TEM)(**a, b**). *S. aureus* ATCC 6538, incubated with 10 mM NaP, pH 5.5, containing 0.25% glucose, for 90 min at 37°C, served as control (**c, d**). Note the condensation of electron-dense cytoplasmic material in LCE3B$_{56\text{-}68}$-treated bacteria (**a, b**). Images are representative of two independent experiments, sampling on average 10 images.

Supplementary Figure 22 a, b

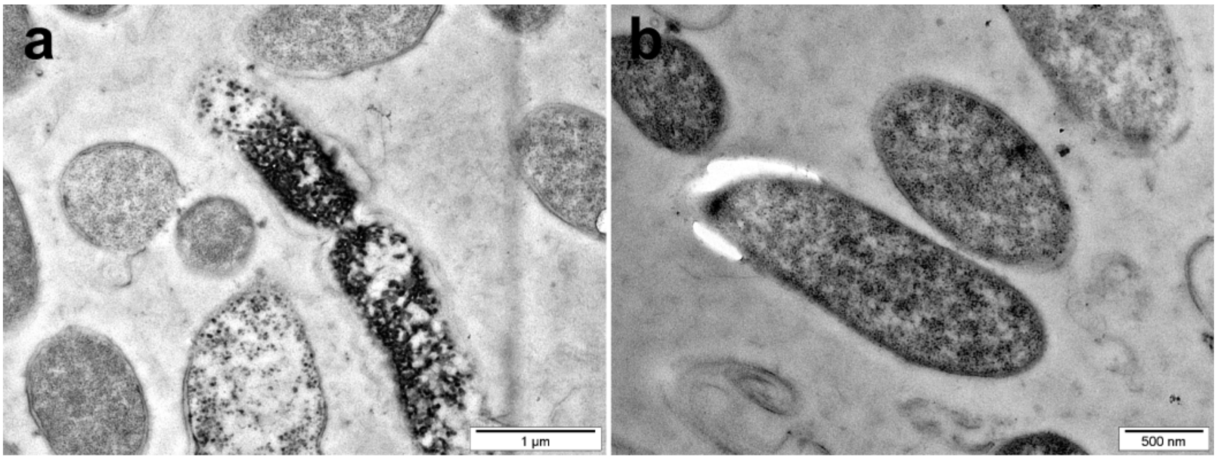

Supplementary Figure 22 a, b I TEM of Pal-GR13-treated *E. coli*. 10^7 *E. coli* ATCC 11775, suspended in 150µl 10 mM NaP, pH 7.3, containing 1% TSB, were treated with Pal-GR13 (Pal-GRRGGRGGRGRGR, 375 µg/ml) for 60 min at ambient temperature and then analyzed by transmission electron microscopy (TEM) (**a**). *E. coli* ATCC 11775, incubated with 10 mM NaP, pH 7.3, containing 1% TSB for 60 min at ambient temperature, served as control (**b**). Note condensation of electron-dense cytoplasmic material in Pal-GR13-treated bacteria (**a**). Images are representative of two independent experiments, sampling on average 10 images.

Supplementary Figure 22c, d

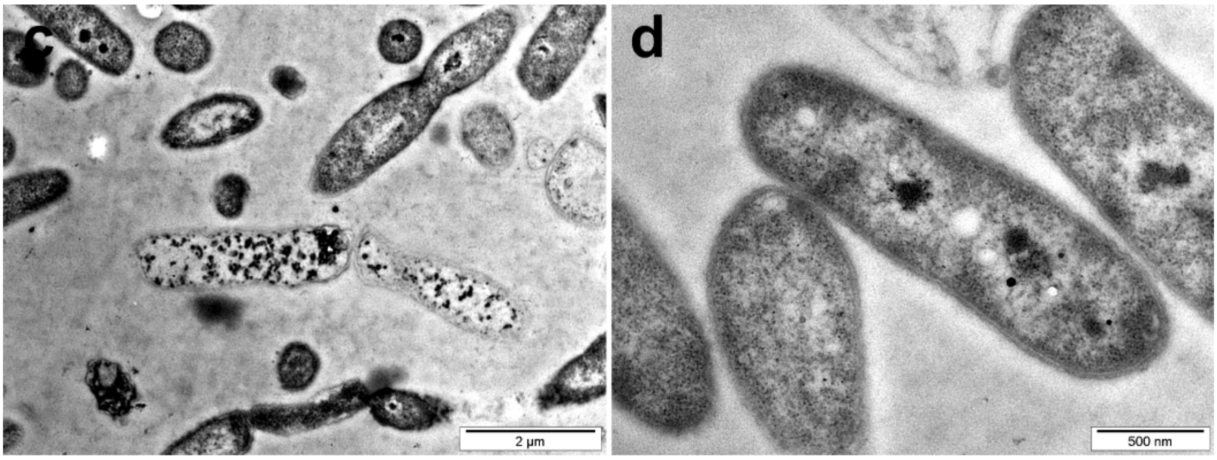

Supplementary Figure 22c, d I TEM of Pal-GR13-treated *Burkholderia cepacia*.
10^7 *Burkholderia cepacia* ATCC 25416, suspended in 150µl 10 mM NaP, pH 7.3, containing 1% TSB, were treated with Pal-GR13 (Pal-GRRGGRGGRGRGR, 375 µg/ml) for 60 min at ambient temperature and then analyzed by transmission electron microscopy (TEM) (**a**). *Burkholderia cepacia* ATCC 25416, incubated with 10 mM NaP, pH 7.3, containing 1% TSB for 60 min at ambient temperature, served as control (**b**). Note condensation of electron-dense cytoplasmic material in Pal-GR13-treated bacteria (**a**). Images are representative of two independent experiments, sampling on average 10 images.

Supplementary Figure 22e, f

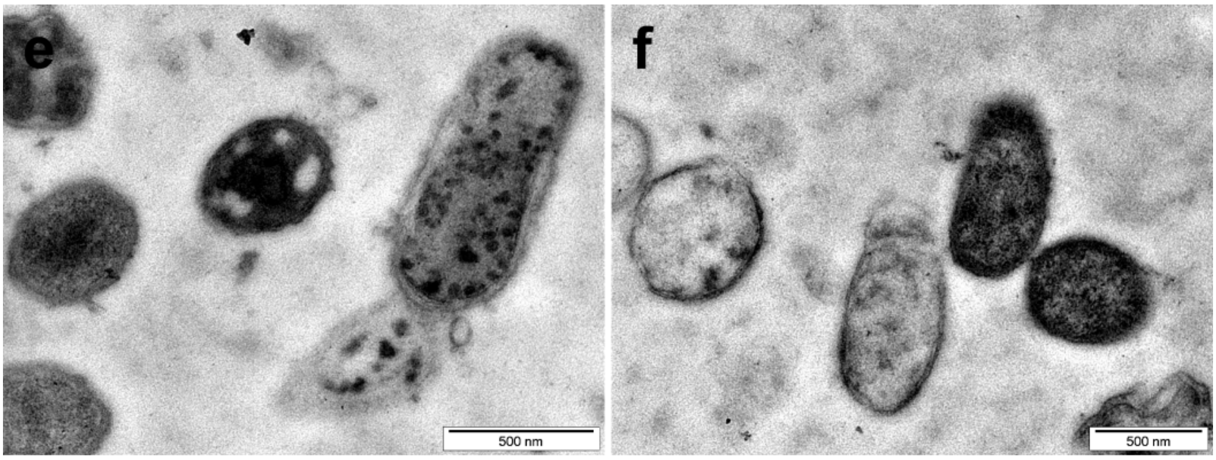

Supplementary Figure 22e, f I TEM of Pal-GR13-treated *Prevotella oralis*. 10^7 *Prevotella oralis* ATCC 33321, suspended in 150µl 10 mM NaP, pH 7.3, containing 1% TSB, were treated with Pal-GR13 (Pal-GRRGGRGGRGRGR, 375 µg/ml) for 60 min at ambient temperature and aerobic conditions and then analyzed by transmission electron microscopy (TEM)(**a**). *Prevotella oralis* ATCC 33321, incubated with 10 mM NaP, pH 7.3, containing 1% TSB for 60 min at ambient temperature and aerobic conditions, served as control (**b**). Note condensation of electron-dense cytoplasmic material in Pal-GR13-treated bacteria (**a**). Images are representative of two independent experiments, sampling on average 10 images.

Supplementary Figure 22g, h

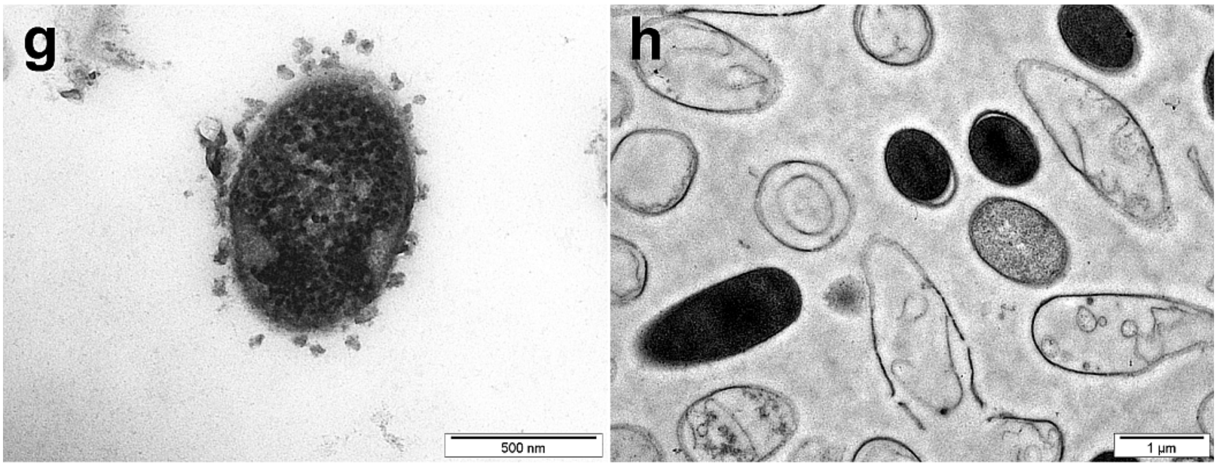

Supplementary Figure 22g, h I TEM of Pal-GR13-treated *Clostridium perfringens*.

10^7 *Clostridium perfringens* ATCC 13124, suspended in 150µl 10 mM NaP, pH 7.3, containing 1% TSB, were treated with Pal-GR13 (Pal-GRRGGRGGRGRGR, 375 µg/ml) for 60 min at ambient temperature and aerobic conditions and then analyzed by transmission electron microscopy (TEM)(**a**). *Clostridium perfringens* ATCC 13124, incubated with 10 mM NaP, pH 7.3, containing 1% TSB for 60 min at ambient temperature and aerobic conditions, served as control (**b**). Note condensation of electron-dense cytoplasmic material and liberation of mesosome-like vesicles in Pal-GR13-treated bacteria (**a**). Images are representative of two independent experiments, sampling on average 10 images.

Supplementary Figure 22j, k

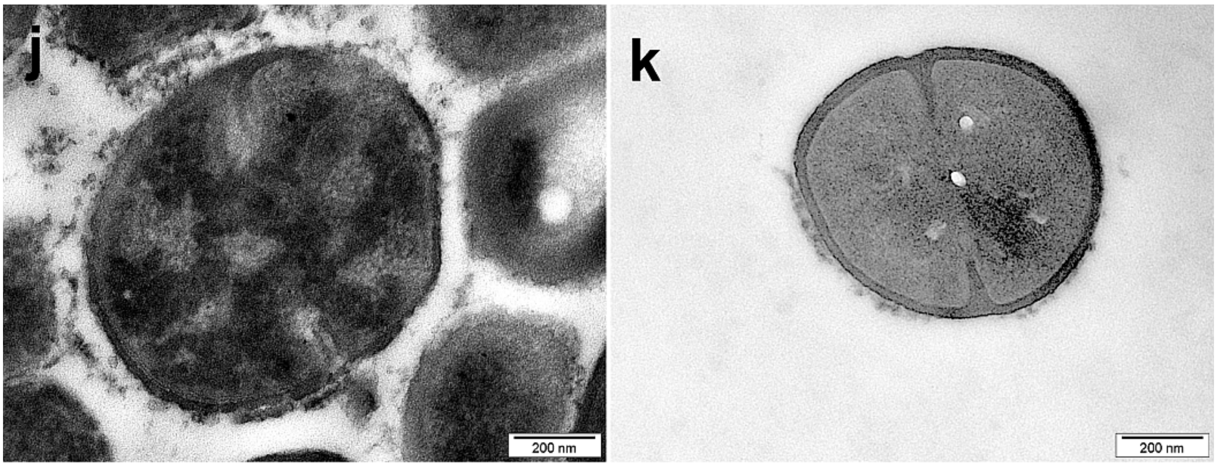

Supplementary Figure 22j, k: TEM of Pal-GR13-treated *Staphylococcus epidermidis*. 10^7 *Staphylococcus epidermidis* ATCC 14990, suspended in 150µl 10 mM NaP, pH 7.3, containing 1% TSB, were treated with Pal-GR13 (Pal-GRRGGRGGRGRGR, 375 µg/ml) for 60 min at ambient temperature and then analyzed by transmission electron microscopy (TEM)(**a**). *Staphylococcus epidermidis* ATCC 14990, incubated with 10 mM NaP, pH 7.3, containing 1% TSB for 60 min at ambient temperature, served as control (**b**). Note condensation of electron-dense cytoplasmic material and liberation of mesosome-like vesicles in Pal-GR13-treated bacteria (**a**). Images are representative of two independent experiments, sampling on average 10 images.

Supplementary Figure 22 l, m

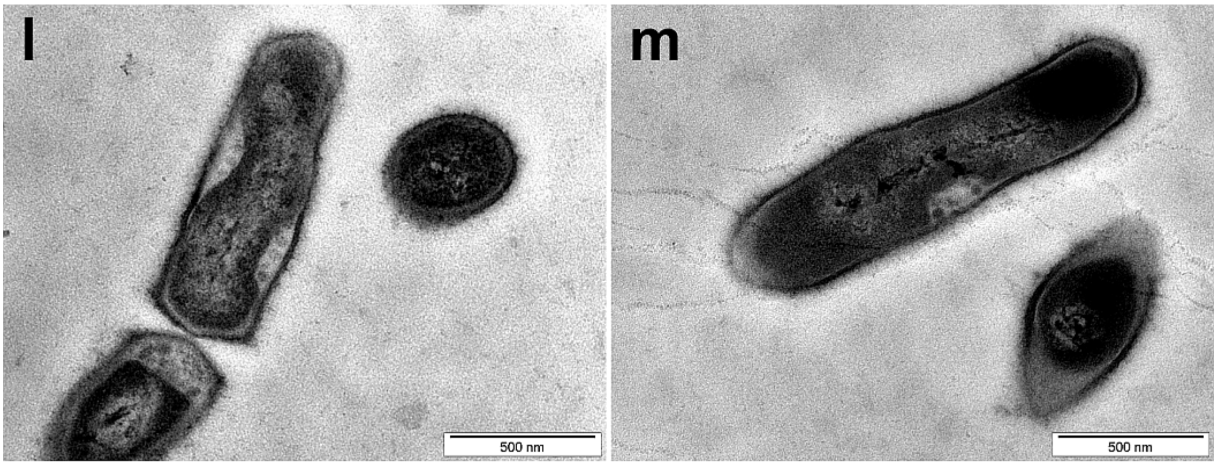

Supplementary Figure 22 l, m I TEM of Pal-GR13-treated *Propionibacterium acnes*. 10^7 *Propionibacterium acnes* ATCC 6919, suspended in 150µl 10 mM NaP, pH 7.3, containing 1% TSB, were treated with Pal-GR13 (Pal-GRRGGRGGRGRGR, 375 µg/ml) for 60 min at ambient temperature and aerobic conditions and then analyzed by transmission electron microscopy (TEM)(**a**). *Propionibacterium acnes* ATCC 6919, incubated with 10 mM NaP, pH 7.3, containing 1% TSB for 60 min at ambient temperature, served as control (**b**). Note condensation of electron-dense cytoplasmic material in Pal-GR13-treated bacteria (**a**). Images are representative of two independent experiments, sampling on average 10 images.

Supplementary Figure 23

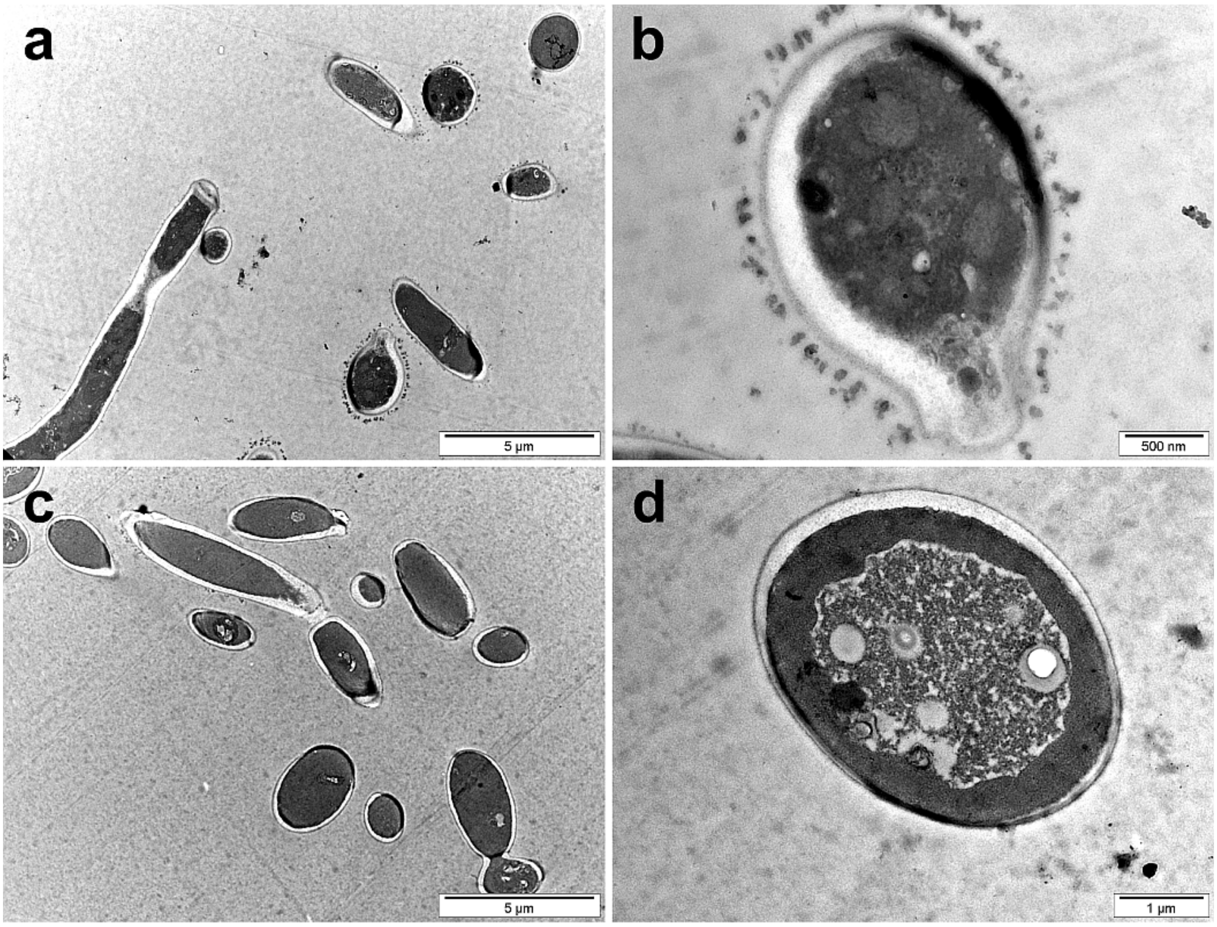

Supplementary Figure 23 I TEM of Pal-GR13-treated *C. albicans*. 10^7 *C. albicans* ATCC 24433, suspended in 150µl 10 mM NaP, pH 7.3, containing 1% TSB, were treated with Pal-GR13 (Pal-GRRGGRGGRGRGR, 375 µg/ml)for 60 min at ambient temperature and then analyzed by transmission electron microscopy (TEM)(**a, b**). *C. albicans* ATCC 24433, incubated with 10 mM NaP, pH 7.3, containing 1% TSB for 60 min at ambient temperature, served as control (**c, d**). Note nuclear blebs and chromatin margination and condensation in the nucleus in Pal-GR13-treated bacteria (**a, b**). Images are representative of two independent experiments, sampling on average 10 images.

Supplementary Tables

Supplementary Table 1 I Amino acid sequences of recombinant HRNR fragments used in this study.

Name	Amino Acid Sequence
**rHRNR$_{1075-1172}$**	THGQHGSTSGQSSSCGQHGASSGQSSSHGQHGSGSSQSSGYGRQGSGSGQSPGHGRGSGSRQSPSYGRHGSGSGRSSSSGQHGSGLGESSGFGHHES
**rHRNR$_{2591-2684}$**	SQHGSGSGHSSGYGQHGSRSGQSSRGERHGSSSGSSSHYGQHGSGSRQSSGHGRQGSGSGQSPSRGRHGSGYGHSSSHGQHGSGSGRSSSRGPY
**rHRNR$_{2638-2684}$**	QSSGHGRQGSGSGQSPSRGRHGSGYGHSSSHGQHGSGSGRSSSRGPY
**rHRNR$_{2656-2684}$**	GRHGSGYGHSSSHGQHGSGSGRSSSRGPY
**rHRNR$_{2591-2644}$**	SQHGSGSGHSSGYGQHGSRSGQSSRGERHGSSSGSSSHYGQHGSGSRQSSGHGR
**rHRNR$_{2576-2707}$**	GFGHHESSSGQSSSYSQHGSGSGHSSGYGQHGSRSGQSSRGERHGSSSGSSSHYGQHGSGSRQSSGHGRQGSGSGQSPSRGRHGSGYGHSSSHGQHGS<br>GSGRSSSRGPYESRSGHSSVFGQHESGSGHSSAY
**rHRNR$_{2727-2850}$**	SGQSSTFDQEGSSTGQSSSYGHRGSGSSQSSGYGRHGAGSGQSLSHGRHGSGSGQSSSYGQHGSGSGQSSGYSQHGSGSGQDGYSYCKGGSNHDGGS<br>SGSYFLSFPSSTSPYEYVQEQRCYFYQ
**SUMO3-His-tag**	MGHHHHHHGGMSEEKPKEGVKTENDHINLKVAGQDGSVVQFKIKRHTPLSKLMKAYCERQGLSMRQIRFRFDGQPINETDTPAQLEMEDEDTIDVFQQQTGG

Residues cationic at pH 7, cationic only at acidic pH and anionic at pH >5 are colored blue, green and magenta, respectively.

Supplementary Table 2 I Tryptic soybean broth (TSB) inhibits *P. aeruginosa* sensitivity towards HRNR$_{2656\text{-}2677}$ (HR 1-18).

AA-sequence	Net charge*	Name	LD90 (µg/ml)	LD100 (µg/ml)	Condition**
GRHGSGLGHSSSHGQHGSGSGR	+6	HR 1-18, HRNR$_{2656\text{-}2677}$	0.038	0.15	0% TSB
GRHGSGLGHSSSHGQHGSGSGR	+6	HR 1-18, HRNR$_{2656\text{-}2677}$	1.18	9.38	1% TSB
GRHGSGLGHSSSHGQHGSGSGR	+6	HR 1-18, HRNR$_{2656\text{-}2677}$	37.5	>300	3% TSB
GRHGSGLGHSSSHGQHGSGSGR	+6	HR 1-18, HRNR$_{2656\text{-}2677}$	>300	>300	10% TSB

*At pH 5.5. **Sensitivity of *P. aeruginosa* ATCC 10145 was examined in 10 mM NaP/0.25% glucose/pH 5.5. Residues cationic at pH 7 and cationic only at acidic pH are colored blue and green, respectively. Representatives are shown (n=2).

Supplementary Table 3 I *P. aeruginosa*-cidal activity of HR 1-18 and its variants.

AA-sequence	Net charge*	Name	n	LD90 (µg/ml)	LD100 (µg/ml)	Condition***
GRHGSGLGHSSSHGQHGSGSGR	+6	HR 1-18, $HRNR_{2656\text{-}2677}$	7	0.038	0.15	pH5.5
LRHGSGLGHSSSHGQHGSGSGR	+6	HR 1-18-Leu	2	4.7	>300	pH5.5
GRHGSGGGHSSSHGGHGSGSGR	+5	HR 1-18,L-G/Q-G	2	0.038	0.15	pH5.5
GRHGGGGGHGGGHGGHGGGGGR	+6	HR 1-18,L-G/Q-G/6S-6G	2	0.038	0.15	pH5.5
GHHGGGGGHGGGHGGHGGGGGH	+6	HR 1-18,G+H	2	**<0.019**	**0.075**	**pH5.5**
GHHGGGGGHGGGHGGHGGGGGH	0	HR 1-18,G+H	2	**>300**	**>300**	**pH7.3**
GRRGSGLGRSSSRGGRGSGSGR	+6	HR 1-18,4H-4R	2	0.038	0.15	pH5.5
GRRGGGGGRGGGRGGRGGGGGR	+6	HR 1-18,G+R	2	<0.019	0.075	pH5.5
GRRGSGGGRSSSRGGRGSGSGR	+6	HR 1-18,4H-4R/L-G	2	0.038	0.15	pH5.5
SRRSSSLSRSSSRSSRSSSSSR	+6	HR 1-18,4H-4R/9G-9S	2	0.15	1.18	pH5.5
Biot-GRHGSGLGHSSSHGQHGSGSGR	+6	Biotin-HR1-18	2	>300/1.18**	>300	pH5.5

*At pH 5.5. **Antimicrobial paradox[16]. ***Sensitivity of *P. aeruginosa* ATCC 10145 was examined in 10 mM NaP/0.25% glucose/pH 5.5 or pH 7.3. In AA-sequences, residues cationic at pH 7 and cationic only at acidic pH are colored blue and green, respectively. AA replaced by Gly, Arg, Ser or Leu or biotinylated are colored magenta. Note the increase of the LD in a Leu-mutant of HR1-18 (colored yellow) and the loss of antimicrobial activity in a Gly- and His-containing HR1-18 mutant at neutral pH (LD values colored blue and red, respectively). n: number of experiments.

Supplementary Table 4 I *P. aeruginosa*-killing by HR1-17 mutants.

AA-sequence	Name	Net charge*	LD90 (µg/ml)	LD100 (µg/ml)
GRHGSGLGHSSSHGQHGSGSGRSSSRGPY	HR 1-17, HRNR$_{2656-2684}$	+7	0.038	0.15
GGHGSGLGHSSSHGQHGSGSGRSSSRGPY	HR 1-17R1-G, HRNR$_{2656-2684}$ R2657G	+6	0.038	0.3
GRHGSGLGHSSSHGQHGSGSGGSSSRGPY	HR 1-17R2-G, HRNR$_{2656-2684}$ R2677G	+6	0.038	0.15
GRHGSGLGHSSSHGQHGSGSGRSSSGGPY	HR 1-17R3-G, HRNR$_{2656-2684}$ R2681G	+6	0.038	0.15-0.3
GRGGSGLGGSSSGGQGGSGSGRSSSRGPY	HR 1-174H-4G, HRNR$_{2656-2684}$ all H vs G	+3	75	>300

*At pH 5.5. Residues cationic at pH 7 and cationic only at acidic pH are colored blue and green, respectively. Amino acids replaced in wild type HR1-17 by Gly are colored magenta. *P. aeruginosa* ATCC 10145-cidal activity was examined in 10 mM NaP/0.25% glucose/pH 5.5. All data shown are representatives (n=2).

Supplementary Table 5 I *P. aeruginosa*-cidal activity of HR1-18 does not depend on its AA-sequence and chirality.

AA-sequence	Net charge*	Name	LD90 (µg/ml)	LD100 (µg/ml)
GRHGSGLGHSSSHGQHGSGSGR	+6	HR 1-18, $HRNR_{2656\text{-}2677}$	0.075	0.59
$GR_DH_DGS_DGL_DGH_DS_DS_DS_DH_DGQ_DH_DGS_DGS_DGR_D$	+6	(D)HR 1-18, $HRNR_{2656\text{-}2677}$	0.038	0.3
RGSGSGHQGHSSSHGLGSGHRG	+6	rev-HR 1-18	<0.019	0.3
SLSSGHGSGHGHQRGGHRSGSG	+6	scr-HR 1-18	<0.019	0.15

*At pH 5.5. Residues cationic at pH 7 and cationic only at acidic pH are colored blue and green, respectively. *P. aeruginosa* ATCC 10145-cidal activity was examined in 10 mM NaP/0.25% glucose/pH 5.5. Data are representatives (n=3).

Supplementary Table 6 I HRNR peptides and its mutants are *P. aeruginosa*-cidal CIDAMPs.

AA-sequence	Net charge*	Name	n	LD90 (µg/ml)	LD100 (µg/ml)	Condition***
QSPSY	±0	HR 2-3, $HRNR_{1137-1141}$	1	>300	>300	pH5.5, 1% TSB
SSSSR	+1	HR 1-9, $HRNR_{2678-2681}$	1	>300	>300	pH5.5, 1% TSB
SGQSSR	+1	HR 1-2, $HRNR_{2610-2615}$	1	>300	>300	pH5.5, 1% TSB
SSSRGPY	+1	HR 1-10, $HRNR_{2678-2684}$	1	>300	>300	pH5.5, 1% TSB
QSPSYGR	+1	HR 2-6, $HRNR_{1137-1143}$	1	>300	>300	pH5.5, 1% TSB
SGQSSRGER	+1	HR 1-3, $HRNR_{2610-2618}$	1	>300	>300	pH5.5, 1% TSB
GSGSRQSPSY	+1	HR 2-2, $HRNR_{1132-1141}$	1	>300	>300	pH5.5, 1% TSB
QGSGSGQSPSR	+1	HR 1-6, $HRNR_{2645-2655}$	1	>300	>300	pH5.5, 1% TSB
QSSGHGR	+2	HR 1-5, $HRNR_{2638-2644}$	1	>300	>300	pH5.5, 1% TSB
HGSGSGR	+2	HR 2-9, $HRNR_{1144-1150}$	1	>300	>300	pH5.5, 1% TSB
GSGSRQSPSYGR	+2	HR 2-8, $HRNR_{1132-1143}$	1	37.5	>300	pH5.5, 1% TSB
QGSGSGQSPSRGR	+2	HR 1-7, $HRNR_{2645-2657}$	1	>300	>300	pH5.5, 1% TSB
QSPSYGRHGSGSGR	+3	HR 2-5, $HRNR_{1137-1150}$	2	>300	>300	pH5.5, 1% TSB
GSGSRQSPSYGRQGSGSGR	+3	HR 2-4Q, $HRNR_{1132-1150}$ H1144Q	1	>300	>300	pH5.5, 1% TSB
GSGSRQSSGHGRQGSGSGQ	+3	HR 1-16, $HRNR_{2633-2651}$	1	>300	>300	pH5.5, 1% TSB
QGSGSGQSPGHSQRGSGSRQSPSY	+3	HR 2-1, $HRNR_{1118-1141}$	1	>300	>300	pH5.5, 1% TSB
GRHGSGSGQSSSYSPYGSGSGWSSSR	+3	$HRNR_{2422-2447}$	2	18.75/150**	>300	pH5.5, 1% TSB
GRHGSGSGQSSSYGPYGSGSGWSSSRGPY	+3	$HRNR_{1952-1980}$	2	18.75/150**	>300	pH5.5, 1% TSB
GRHGSGSGQSSSYSPYGSGSGWSSSRGPY	+3	$HRNR_{2422-2450}$	2	2.35/>300**	>300	pH5.5, 1% TSB
RHGSGLGHSSSH	+4	HR 1-27, $HRNR_{2657-2668}$	1	75-100*	1.18/300*	pH5.5, 0.25% glucose
GRHGSGLGHSSSH	+4	HR 1-37, $HRNR_{2656-2668}$	2	9.38	>300	pH5.5, 0.25% glucose
HSSSHGQHGSGSGR	+4	HR 1-38, $HRNR_{2664-2677}$	2	>300/9.38**	>300	pH5.5, 0.25% glucose
SRQSPSYGRHGSGR	+4	HR 2-15, $HRNR_{1135-1148}$ S1148R	1	75	>300	pH5.5, 1% TSB
RQSPSYGRHGSGSGR	+4	HR 2-14, $HRNR_{1136-1150}$	1	300	>300	pH5.5, 1% TSB
GSGSRQSPSYGRHGSGSGR	+4	HR 2-4, $HRNR_{1132-1150}$	4	>300	>300	pH5.5, 1% TSB
SRQSPSYGRHGSGSGR	+4	HR 2-13, $HRNR_{1135-1150}$	1	>300	>300	pH5.5, 1% TSB
GSRQSPSYGRHGSGSGR	+4	HR 2-12, $HRNR_{1134-1150}$	1	>300	>300	pH5.5, 1% TSB
GSGSRQSSSYGRHGSGSGR	+4	HR 2-4S, $HRNR_{1132-1150}$ P1139S	2	150	>300	pH5.5, 1% TSB
GSGSRQSLSYGRHGSGSGR	+4	HR 2-4L, $HRNR_{1132-1150}$ P1139L	1	75	>300	pH5.5, 1% TSB
HGSSSGSSSHYGQHGSGSR	+4	HR 1-4, $HRNR_{2619-2637}$	1	150	>300	pH5.5, 1% TSB
SQHGSGSGHSSGYGQHGSR	+4	HR 1-1, $HRNR_{2591-2609}$	1	>300	>300	pH5.5, 1% TSB
GRHGSGSGQSSSYGPYRSGSGWSSSRGPY	+4	$HRNR_{1013-1041}$	2	1.18/75**	>300	pH5.5, 1% TSB
RHGSGLGHSSSHGQH	+5	HR 1-26, $HRNR_{2657-2671}$	2	0.3/150**	>300/2,35	pH5.5, 0.25% glucose
GRHGSGLGHSSSHGQH	+5	HR 1-25, $HRNR_{2656-2671}$	2	0.3	4.7	pH5.5, 0.25% glucose
HSQRGSGSRQSPSYGRH	+5	HR 2-16, $HRNR_{1128-1144}$	2	0.59/300**	37.5	pH5.5, 0.25% glucose
GSGSRQSPSRGRHGSGSGR	+5	HR 2-4R, $HRNR_{1132-1150}$ Y1141R	2	37.5/300**	>300	pH5.5, 1% TSB
GSGSRQSPSHGRHGSGSGR	+5	HR 2-4H, $HRNR_{1132-1150}$ Y1141H	2	75	>300	pH5.5, 1% TSB
HGSGLGHSSSHGQHGSGSGR	+5	HR 1-8, $HRNR_{2658-2677}$	1	18.75/>300**	>300	pH5.5, 1% TSB
HGSGLGHSSSHGQHGSGSGR	+5	HR 1-8, $HRNR_{2658-2677}$	1	4.7	>300	pH5.5, 0.25% glucose
HGSRSGQSSRGERHGSSSGSSSH	+5	HR1-11, $HRNR_{2606-2628}$	2	0.075	2.35	pH5.5, 0.25% glucose
GSGSRQSPSYGRHGSGSGRSSSSGQH	+5	HR 2-11 , $HRNR_{1132-1157}$	2	37.5	>300	pH5.5, 1% TSB
GRHGSGSGHSSSHGQHGSGSGR	+6	$HRNR_{2186-2207}$	2	0.15/75**	4.7-9.37**	pH5.5, 0.25% glucose
GRHGSGLGHSSSHGQHGSGSGR	+6	HR 1-18, $HRNR_{2656-2677}$	7	0.038	0.15	pH5.5, 0.25% glucose
LRHGSGLGHSSSHGQHGSGSGR	+6	HR 1-18-Leu,	2	4.7	>300	pH5.5, 0.25% glucose
LGHSSSHGQHGSGSGRSSSRGPYESRLGH	+6	HR 1-36, $HRNR_{2663-2690}$	2	0.15	2.35	pH5.5, 0.25% glucose
GRHGSGLGHSSSHGQHGSGSGRSSSRGPY	+7	HR 1-17, $HRNR_{2656-2684}$	2	0.038	0.15	pH5.5, 0.25% glucose
GHKSSSGQSSGYTQHGSGSGHSSSYEQHGSRSG QSSRSEQHGSSSGSSSSYGQHGSGSRQSL	+8	$HRNR_{933-994}$	2	0.3	2.35	pH5.5, 0.25% glucose
GHGRQGSGSRQSPSHVRHGSGSGHSSSHGQHGS GSSYSYSRGHYESGSGQTSGFGQHESGSGQSSGY	+9	$HRNR_{295-361}$	2	0.15	0.59	pH5.5, 0.25% glucose
LGHGQHGSGSGQSPSPSRGRHGSGSGQSSSYGP YRSGSGWSSSRGPYESGSGHSSGLGHRER	+9	$HRNR_{995-1056}$	2	0.3	2.35	pH5.5, 0.25% glucose
SQHKSSSGQSSGYSQHGSGSGHSSGYGQHGSRSG QSSRGDRHRSSSGSSSSYGQHGSGSRQSL	+10	$HRNR_{232-294}$	2	0.3	2.35	pH5.5, 0.25% glucose

*At pH 5.5. **Antimicrobial paradox[16]. ***Sensitivity of *P. aeruginosa* ATCC 10145 was examined in 10 mM NaP. Residues cationic at pH 7, cationic only at acidic pH, anionic and mutated are colored blue, green, magenta and yellow, respectively. Note the marked increase of the LD value when a selected AA is replaced in defined HRNR-peptides (LD values colored in yellow). n: number of experiments.

Supplementary Table 7 I IDPRs of FLG-2, FLG and Repetin are *P. aeruginosa*-cidal CIDAMPs.

AA-sequence	Name	n	Net charge*	LD90 (µg/ml)	LD100 (µg/ml)
TAGRRGSGH	FLG-$2_{2097-2105}$	1	+3	>300	>300
GSRTAGRRGSGH	FLG-$2_{2094-2105}$	1	+4	>300/2.35**	>300
HAHSGHGQSTQR	FLG-$2_{2082-2093}$	2	+4	0.15	2.35
HTHSGHTHGQSGSQHGESESIIHDR	FLG-$2_{1515-1539}$	2	+4	0.075	0.59
HAHSGHGQSTQRGSR	FLG-$2_{2082-2096}$	2	+5	0.15	1.18
HTHSGHTHGQAGSHYPESGSSVHER	FLG-$2_{1969-1993}$	2	+5	0.038	0.3
HTHSGHAHGQAGSQHGESGSSVHER	FLG-$2_{2044-2068}$	1	+5	0.038	0.3
HTHSGHTHGQARSQHGESESIVHER	FLG-$2_{1742-1766}$	2	+5	0.038	0.15
THEHEQSHQRRDRQTHEDKQNRQR	$RPTN_{747-770}$	2	+5	0.3	150
HGQSTQRGSRTAGRRGSGH	FLG-$2_{2087-2105}$	2	+6	0.075	1.18
HAHSGHGQSTQRGSRTAGR	FLG-$2_{2082-2100}$	2	+6	0.038	0.3
HAHYGYGQSTQRGSRTTGRRGSGH	FLG-$2_{2232-2255}$	2	+7	0.075	0.59
QGSHHEQSVNRSGHSGSHHSHTTSQGR	$FLG_{528-554}$	2	+7	0.15	0.59
HTHSGHTHSQARSQHGESESTVHKR	FLG-$2_{1894-1918}$	2	+7	0.038	0.3
HAHSGHGQSTQRGSRTAGRRGSGH	FLG-$2_{2082-2105}$	2	+8	< 0.038	0.075

*At pH 5.5. **Antimicrobial paradox[16]. Residues cationic at pH 7, cationic only at acidic pH and anionic are colored blue, green and magenta, respectively. Sensitivity of *P. aeruginosa* ATCC 10145 was tested in 10 mM NaP/0.25% glucose/pH5.5. n: number of experiments.

Supplementary Table 8 I CIDAMPs with *S. aureus* ATCC 6538-cidal activity

AA-sequence	Name	Net charge*	n	LD90 (µg/ml)	LD100 (µg/ml)	Condition**
GRHGSGLGHSSSHGQHGSGSGR	HR 1-18, $HRNR_{2656-2677}$	+6	2	>300	>300	pH5.5
LRHGSGLGHSSSHGQHGSGSGR	HR 1-18-Leu G2656Leu	+6	2	300	>300	pH5.5
GRRGSGLGRSSSRGGRGSGSGR	HR 1-18,4H-4R	+6	2	150	>300	pH5.5
GRRGSGLGRSSSRGGRGSGSGR	HR 1-18,4H-4R	+6	2	9. 375	>300, max.killing: 98.8%	pH7.0
GRRGSGGGRSSSRGGRGSGSGR	HR 1-18,4H-4R/L-G	+6	1	>300	>300	pH5.5
GRRGGGGGRGGGRGGRGGGGGR	HR 1-18,G+R	+6	1	>300	>300	pH5.5
SRRSSSLSRSSSRSSRSSSSSR	HR 1-18,4H-4R/9G-9S	+6	1	150	>300	pH5.5
SRRSSSLSRSSSRSSRSSSSSR	HR 1-18,4H-4R/9G-9S	+6	2	75	>300	pH7.2
GRRGGRGGRGRGR	GR13	+6	2	75	>300	pH5.5
GRRGGRGGRGRGR	GR13	+6	1	75	>300	pH7.0
SHHRCCRSHRCRR	$LCE\text{-}3B_{56-68}$	+8	2	**0.075**	>150, max.killing: 99.3%	pH5.5
SHHRHFRSHQCRR	$LCE\text{-}3C_{55-67}$	+8	2	2.35	>150, max.killing: 97.7%	pH5.5
SHHRHFRSHQCRRQRSNSCDR	$LCE\text{-}3C_{55-75}$	+9	2	0.59	>150, max.killing: 99.4%	pH5.5
SHHRPRLFHRRRH	$LCE\text{-}2B_{74-86}$	+9	2	1.18	>150, max.killing: 97.4%	pH5.5

*At pH 5.5. ** *S. aureus* ATCC 6538 sensitivity was examined in 10 mM NaP/0.25% glucose/pH5.5, pH 7.0 or pH 7.2. Residues cationic at pH 7 and cationic only at acidic pH are colored blue and green, respectively. Amino acids in HR1-18 replaced by Gly, Arg, Ser or Leu are colored magenta. Note the higher antimicrobial potency of selected CIDAMPs at neutral pH (shaded) and the highest potency of the "Late-Cornified-Envelope (LCE) protein" fragment $LCE\text{-}3B_{56-68}$ at acidic pH (bold). n: number of experiments.

Supplementary Table 9 I *S. aureus* ATCC 6538 -bactericidal activity of S-palmitoylated HRNR-CIDAMPs.

AA-sequence	Name	n	Net charge	LD90 (µg/ml)	LD100 (µg/ml)	Condition***
GQHGSSSGHSSTHGQHGSTSGQSSSC-Pal*	$HRNR_{598-623}$	2	+4	2.35	>150	pH5.5, 0.25% glucose
GQHGSSSGHSSTHGQHGSASGQSSSC-Pal*	$HRNR_{2004-2029}$	2	+4	0.59	>150/75**	pH5.5, 0.25% glucose
Pal-CGQHGATSGQSSSHGQHGSGSSQSSR	$HRNR_{623-648}$	2	+4	2.35	>150	pH5.5, 0.25% glucose
Pal-CGQHGATSSGQSSSHGQHGSGSSQSSGYGR	$HRNR_{1089-1117}$	2	+4	>150	>150	pH5.5, 0.25% glucose
GRHGSGSGQSSGFGHHESSSWQSSGC-Pal*	$HRNR_{1389-1414}$	2	+4	1.18	18.8	pH5.5, 0.25% glucose
SGHSSVFGQHESGSGHSSAYSQHGSGSGHFC-Pal*	$HRNR_{1748-1778}$	2	+4	0.3	9.38	pH5.5, 0.25% glucose

*amide, **antimicrobial paradox[16]. ***Antimicrobial activity was examined in 10 mM NaP. Residues cationic at pH 7, cationic only at acidic pH, anionic and S-palmitoyl-Cys are colored blue, green, magenta and yellow, respectively. Hydrophobic residues are shaded grey. n: number of experiments.

Supplementary Table 10 I Sensitivity of *Corynebacterium simulans* and *P. aeruginosa* towards selected FLG- and HRNR-derived CIDAMPs

AA-sequence	Name	n	Net charge*	LD90 *C. simulans* (µg/ml)	LD100 *C. simulans* (µg/ml)	LD90 *P. aeruginosa* (µg/ml)	LD100 *P. aeruginosa* (µg/ml)
QGSHHEQSVNRSGHSGSHHSHTTSQGR	$FLG_{528-554}$	1/2	+7	>150	>150	0.15	0.59
HTQNSSSGQAASSHEQAR	$FLG_{684-701}$	1/1	+3	>150	>150	>300	>300
QSGTRHETSSGGQAASSQEQAR	$FLG_{1652-1674}$	1/1	+1	>150	>150	>300	>300
QSGTHHAENSSGGQAASSHEQAR	$FLG_{2301-2323}$	1/1	+2	>150	>150	>300	300
QSGTRHTQTSSGGQAASSHEQAR	$FLG_{2625-2647}$	1/1	+3	>150	>150	>300	>300
QSGTRHAETSSGGQAASSHEQAR	$FLG_{3273-3295}$	1/1	+2	>150	>150	>300	300
SQHKSSSGQSSGYSQHGSGSGHSSGYGQHGSRSGQSSR GDRHRSSSGSSSSYGQHGSGSRQSL	$HRNR_{232-294}$	2/1	+11	1.18	>150/37.5**	0.3	2.35
GHGRQGSGSRQSPSHVRHGSGSGHSSSHGQHGSGSSYS YSRGHYESGSGQTSGFGQHESGSGQSSGY	$HRNR_{295-361}$	2/1	+10	0.59	75/9.375**	0.15	0.59
GHKSSSGQSSGYTQHGSGSGHSSSYEQHGSRSGQSSRS EQHGSSSGSSSSYGQHGSGSRQSL	$HRNR_{933-994}$	1/1	+8	4.7	>150	0.3	2.35
LGHGQHGSGSGQSPSPSRGRHGSGSGQSSSYGPYRSGS GWSSSRGPYESGSGHSSGLGHRESR	$HRNR_{995-1056}$	1/1	+8	4.7	>150	0.3	2.35

*at pH 5.5. **antimicrobial paradox[16]. Residues cationic at pH 7, cationic only at acidic pH and anionic are colored blue, green and magenta, respectively. Sensitivity of *P. aeruginosa* ATCC 10145 towards the peptides was tested at pH 5.5 and towards *Corynebacterium simulans* DSM 44415 it was tested at pH 6.0 in 10 mM NaP/0.25% glucose. n: number of experients.

Supplementary Table 11 I Palmitoylated or myristoylated CIDAMPs containing Arg, His, Lys, Dab or Dap as cationic AAs are bactericidal for *P. aeruginosa* ATCC 10145

AA-sequence	Name	n	LD100 (µg/mL)	LD90 (µg/mL)	Condition
Pal-GRHGSGLGHSSSHGQHGSGSGR	Pal-HR 1-18, Pal-HRNR$_{2656-2677}$	2	0.59	0.075	pH5.5, 0.25% glucose
Myr-GRHGSGLGHSSSHGQHGSGSGR	Myr-HR 1-18, MyrHRNR$_{2656-2677}$	1	0.3	0.075	pH5.5, 0.25% glucose
Pal-GRRGSGLGRSSSRGQRGSGSGR	Pal-HR 1-18-HR	2	0.59	0.038	pH5.5, 0.25% glucose
Pal-GRRGSGLGRSSSRGQR GSGSGR	Pal-HR 1-18-HR	1	4.7	1.18	pH7.3, 1% TSB
Pal-HGSRSGQSSRGERHGSSSGSSSH	HR 1-11, HRNR$_{2606-2628}$	2	0.3	0.075	pH5.5, 0.25% glucose
Pal-RGSRSGQSSRGERRGSSSGSSSR	Pal-HR 1-11-3H3R	2	0.15	0.038	pH5.5, 0.25% glucose
Pal-RGSRSGQSSRGERR	Pal-HR 1-11-2H2R-14	1	0.15	0.038	pH5.5, 0.25% glucose
Pal-GHHGGHGGHGHGH	Pal-GH13	1	0.3	0.075	pH5.5, 0.25% glucose
Pal-GRRGGRGGRGRGR	Pal-GR13	1	0.3	0.038	pH5.5, 0.25% glucose
Pal-G$R_D R_D$GGR_DGGR_DGR_DGR_D	(D)-Pal-GR13	1	0.3	0.075	pH5.5, 0.25% glucose
Pal-GRRGGRGGRGR	Pal-GR11	2	0.3	0.075	pH5.5, 0.25% glucose
Pal-GRRGGRGGR	Pal-GR9	1	0.59	0.038	pH5.5, 0.25% glucose
Pal-G$R_D R_D$GGR_DGGR_D	(D)-Pal-GR9	1	0.59	0.038	pH5.5, 0.25% glucose
Pal-GRRGGR	Pal-GR6	1	4.7	1.18	pH5.5, 0.25% glucose
Pal-GRGR	Pal-GR4	2	9.375	2.35	pH5.5, 0.25% glucose
Pal-GR_DGR_D	Pal-(D)-GR4	1	150	18.75	pH5.5, 0.25% glucose
Pal-GKKGGKGGKGKGK	Pal-K-GR13	1	0.15	<0.019	pH5.5, 0.25% glucose
Pal-GOOGGOGGOGOGO	Pal-Orn-GR13	1	0.3	0.075	pH5.5, 0.25% glucose
Pal-GKGK	Pal-GK4	2	9.375	2.38	pH5.5, 0.25% glucose
Pal-GK_DGK_D	Pal-(D)-GK4	1	4.7	1.18	pH5.5, 0.25% glucose
Pal-GOGO	Pal-GO4	1	18.75	2.35	pH5.5, 0.25% glucose
Pal-GO_DGO_D	Pal-(D)-GO4	1	9.38	1.18	pH5.5, 0.25% glucose
Pal-GlyDabGlyDab	Pal-G-Dab4	1	9.38	2.35	pH5.5, 0.25% glucose
Pal-GlyDapGlyDap	Pal-G-Dap4	2	9.38	2.35	pH5.5, 0.25% glucose
Pal-GRRGSGLGRSSSR	Pal-HR 1-18-3H3R-GR13	2	4.7	0.59	pH5.5, 0.25% glucose

Residues cationic at pH 7, cationic only at acidic pH and anionic at pH 7 are colored blue, green and magenta, respectively. Dab: L-2,4-Diaminobutyric acid, Dap:L-2,3-Diaminopropionic acid. n: number of experiments.

Supplementary Table 12 I Late cornified envelope protein (LCE)-peptide fragments and its palmitoylated derivatives are potent and efficient *S. aureus*-cidal antimicrobials.

AA-sequence	Name	Net Charge*	LD100 (µg/mL)	LD90 (µg/mL)	Condition
SHHRCCRSHRCRR	LCE-3B$_{56\text{-}68}$	+8	>150, max.killing: 99.3%	**0.075**	pH5.5, 0.25% glucose
SHHRHFRSHQCRR	LCE-3C$_{55\text{-}67}$	+8	>150, max.killing: 97.7%	2.35	pH5.5, 0.25% glucose
SHHRHFRSHQCRRQRSNSCDR	LCE-3C$_{55\text{-}75}$	+9	>150, max.killing: 99.4%	0.59	pH5.5, 0.25% glucose
Pal-SHHRCCRSHRCRR	Pal- LCE-3B$_{56\text{-}68}$	+8	0.15	**<0.019**	pH5.5, 0.25% glucose
Pal-SHHRHFRSHQCRR	Pal- LCE-3C$_{55\text{-}67}$	+8	0.3	**0.038**	pH5.5, 0.25% glucose
Pal-SHHRHFRSHQCRRQRSNSCDR	Pal- LCE-3C$_{55\text{-}75}$	+9	0.59	0.15	pH5.5, 0.25% glucose

Residues cationic at pH 7, cationic only at acidic pH and anionic are colored blue, green and magenta, respectively. *: at pH 5.5. Representatives are shown (n=2).

Supplementary Table 13 | Antimicrobial activity spectrum of Palmitoyl-HR1-18.*

Microorganism	n	LD100 (µg/mL)	LD90 (µg/mL)	Conditions
Burkholderia cepacia ATCC 25416	2	>300	75	pH 7.3, 0.25% glucose
Burkholderia cepacia ATCC 25416	2	>300	>300	pH 5.5, 0.25% glucose
Klebsiella pneumoniae ATCC 13883	1	1.18	0.3	pH 5.5, 0.25% glucose
Prevotella oralis ATCC 33321	1	>300	37.5	pH 6.5, 0.25 % glucose
Prevotella oralis ATCC 33321	2	150	18.75	pH 7.3, 1 % TSB
Pseudomonas aeruginosa ATCC10145	2	0.59	0.075	pH 5.5, 0.25% glucose
Clostridium perfringens ATCC 13124	1	37.5	18.75	pH 7.3, 1 % TSB
Clostridium perfringens ATCC 13124	1	75	37.5	pH 5.5, 0.25% glucose
Clostridium perfringens ATCC 13124	1	18.75	4.7	pH 7.3, 0.25% glucose
Propionibacterium acnes ATCC 6919	1	0.59	0.3	pH 5.5, 0.25% glucose
Propionibacterium acnes ATCC 6919	1	4.7	1.18	pH 7.3, 1 % TSB
Propionibacterium acnes ATCC 6919	1	2.35	0.59	pH 7.3, 0.25% glucose
Staphylococcus aureus ATCC 6538	2	37.5	1.18	pH 5.5, 0.25% glucose
Staphylococcus aureus ATCC 6538	1	>300	>300	pH 7.3, 1 % TSB
Staphylococcus epidermidis ATCC 14990	1	>300	>300	pH 7.3, 1 % TSB
Streptococcus pneumoniae ATCC 33400	2	9.37	2.35	pH 6.5, 0.25% glucose
Streptococcus pneumoniae ATCC 33400	1	37.5	9.38	pH 7.3, 1 % TSB
Streptococcus pneumoniae ATCC 33400	1	4.7	2.35	pH 7.3, 0.25% glucose
Streptococcus pyogenes ATCC 12344	2	0.59	0.15	pH 6.0, 0.25% glucose
Streptococcus pyogenes ATCC 12344	1	9.38	9.38	pH 7.3, 1 % TSB
Streptococcus pyogenes ATCC 12344	1	0.3	0.15	pH 7.3, 0.25% glucose

*AA-sequence: Pal-GRHGSGLGHSSSHGQHGSGSGR. Sensitivity of bacteria towards Pal-HR1-18 was tested in a CFU assay system in 10 mM NaP, depending on the microbe, at pH 5.5, 6.5 or pH 7.3, which contained 0.25% glucose and 1% TSB, respectively. n: number of experiments.

Supplementary Table 14 l Antimicrobial activity spectrum of Palmitoyl-HR1-18HR.*

Microorganism	n	LD100 (µg/mL)	LD90 (µg/mL)	Conditions
Burkholderia cepacia ATCC 25416	2	150	75	pH 7.3, 1 % TSB
Burkholderia cepacia ATCC 25416	2	300	75	pH 7.3, 0.25% glucose
Prevotella oralis ATCC 33321	1	75	37.5	pH 7.3, 1 % TSB
Pseudomonas aeruginosa ATCC10145	1	4.7	1.18	pH 7.3, 1 % TSB
Pseudomonas aeruginosa ATCC10145	2	0.59	0.038	pH 5.5, 0.25% glucose
Clostridium perfringens ATCC 13124	1	9.375	4.7	pH 7.3, 0.25% glucose
Propionibacterium acnes ATCC 6919	1	4.7	1.18	pH 7.3, 1 % TSB
Staphylococcus aureus ATCC 6538	2	4.7	0.3	pH 5.5, 0.25% glucose
Staphylococcus aureus ATCC 6538	1	9.38	1.18	pH 7.3, 1 % TSB
Streptococcus pneumoniae ATCC 33400	1	9.38	9.38	pH 7.3, 0.25 % glucose
Streptococcus pyogenes ATCC 12344	1	0.3	0.15	pH 7.3, 0.25 % glucose

* AA-sequence: Pal-GRRGSGLGRSSSRGQRGSGSGR**.** Sensitivity of bacteria towards Pal-HR1-18HR was tested in a CFU assay system in 10 mM NaP, depending on the microbe, which contained 0.25% glucose and 1% TSB, respectively, at pH 5.5 or pH 7.3. n: number of experiments.

Supplementary Table 15 I Results of a CIDAMP-MIC*-screening at CO-ADD

Sample Name	CO-ADD Sample ID	GN_001<br>E. coli<br>ATCC 25922 FDA control	GN_003<br>K. pneumoniae<br>ATCC 700603 MDR	GN_034<br>A. baumannii<br>ATCC 19606 Type strain	GN_042<br>P. aeruginosa<br>ATCC 27853 Type strain	GP_020<br>S. aureus<br>ATCC 43300 MRSA	FN_001<br>C. albicans<br>ATCC 90028 CSLI Ref	FN_002<br>C. neoformans<br>ATCC 20881 H99	MA_007<br>HEK293<br>ATCC CRL-1573
		MIC (µg/mL)							CC_{50} (µg/mL)
LCE-2B74-86	C0137532	>32 (14.6%)<br>>32 (15.9%)	>32 (32.1%)<br>>32 (31.8%)	>32 (17.5%)<br>>32 (38.7%)	>32 (4.8%)<br>>32 (10.9%)	>32 (45.1%)<br>>32 (29.0%)	>32 (4.0%)<br>>32 (-3.2%)	>32 (27.2%)<br>>32 (7.0%)	>32
LCE-3B56-68	C0137533	>32 (22.5%)<br>>32 (17.5%)	>32 (25.7%)<br>>32 (24.1%)	>32 (18.4%)<br>>32 (38.6%)	>32 (1.6%)<br>>32 (11.5%)	>32 (48.1%)<br>>32 (39.5%)	>32 (19.8%)<br>>32 (9.1%)	8 (95.9%)<br>8 (104.2%)	>32
LCE-3C55-67	C0137534	>32 (15.0%)<br>>32 (12.6%)	>32 (23.0%)<br>>32 (19.3%)	>32 (9.6%)<br>>32 (38.9%)	>32 (9.9%)<br>>32 (11.3%)	>32 (47.4%)<br>>32 (36.9%)	>32 (6.2%)<br>>32 (5.7%)	8 (97.8%)<br>8 (102.3%)	>32
LCE-3C55-75	C0137535	>32 (15.5%)<br>>32 (11.7%)	>32 (25.7%)<br>>32 (20.5%)	>32 (7.4%)<br>>32 (17.6%)	>32 (-0.4%)<br>>32 (4.9%)	>32 (41.5%)<br>>32 (25.2%)	>32 (10.0%)<br>>32 (7.8%)	4 (105.8%)<br>4 (105.3%)	>32
Pal-HR 1-18-HR	C0137536	4 (95.0%)<br>8 (96.6%)	32 (92.9%)<br>32 (91.1%)	4 (97.0%)<br>1 (98.3%)	16 (97.5%)<br>32 (96.9%)	4 (94.0%)<br>8 (93.4%)	16 (99.0%)<br>16 (98.9%)	1 (105.0%)<br>1 (106.1%)	>32
Pal-HR-1-11-2H2R-14	C0137537	8 (97.8%)<br>16 (96.8%)	>32 (32.7%)<br>>32 (36.7%)	8 (98.3%)<br>4 (98.2%)	32 (81.8%)<br>32 (93.4%)	16 (95.0%)<br>8 (91.1%)	>32 (36.3%)<br>>32 (31.7%)	4 (107.3%)<br>4 (106.8%)	>32
Pal-GR13	C0137538	4 (96.9%)<br>8 (95.1%)	32 (91.9%)<br>16 (92.3%)	2 (96.8%)<br>4 (97.0%)	8 (98.3%)<br>8 (97.1%)	0.5 (98.6%)<br>0.25 (99.4%)	4 (99.4%)<br>4 (99.1%)	0.25 (106.2%)<br>0.25 (106.6%)	>32
Pal-GR9	C0137539	>32 (28.9%)<br>>32 (35.6%)	>32 (25.4%)<br>>32 (21.3%)	>32 (16.9%)<br>>32 (29.1%)	>32 (13.4%)<br>>32 (12.9%)	32 (84.0%)<br>32 (100.3%)	>32 (5.7%)<br>>32 (2.7%)	>32 (-0.9%)<br>>32 (10.3%)	>32
Pal-GR4	C0137540	>32 (14.2%)<br>>32 (11.2%)	>32 (19.2%)<br>>32 (23.1%)	32 (92.8%)<br>32 (79.6%)	>32 (5.5%)<br>>32 (11.4%)	8 (97.4%)<br>8 (98.1%)	32 (79.8%)<br>8 (86.0%)	2 (103.9%)<br>2 (106.3%)	>32
Pal-G-Dap4	C0137541	>32 (15.7%)<br>>32 (13.5%)	>32 (15.6%)<br>>32 (11.4%)	>32 (15.5%)<br>>32 (25.8%)	>32 (9.5%)<br>>32 (10.6%)	8 (99.2%)<br>8 (98.0%)	8 (97.2%)<br>16 (94.3%)	1 (106.0%)<br>1 (105.5%)	>32
Pal-LCE-3C55-67	C0137542	>32 (15.0%)<br>>32 (17.3%)	>32 (29.9%)<br>>32 (31.2%)	>32 (15.2%)<br>>32 (25.9%)	>32 (10.1%)<br>>32 (10.8%)	>32 (71.3%)<br>>32 (59.0%)	16 (93.9%)<br>16 (93.6%)	2 (103.9%)<br>1 (107.8%)	>32
Pal-LCE-3B56-68	C0137543	>32 (17.3%)<br>>32 (18.9%)	>32 (30.1%)<br>>32 (23.6%)	>32 (11.5%)<br>>32 (30.3%)	>32 (10.5%)<br>>32 (12.5%)	16 (96.9%)<br>16 (97.6%)	1 (98.4%)<br>2 (98.3%)	0.25 (105.7%)<br>0.25 (106.1%)	>32
S-Pal-HRNR1748 - 1778	C0137544	>32 (13.0%)<br>>32 (11.5%)	>32 (17.2%)<br>>32 (22.9%)	>32 (12.7%)<br>>32 (21.6%)	>32 (5.3%)<br>>32 (12.2%)	>32 (59.0%)<br>>32 (32.0%)	>32 (18.4%)<br>>32 (4.1%)	4 (108.6%)<br>4 (104.7%)	>32
Pal-HR 1-18 GR13	C0137546	32 (95.8%)<br>>32 (40.2%)	>32 (19.3%)<br>>32 (13.9%)	16 (99.5%)<br>16 (99.8%)	>32 (19.8%)<br>>32 (20.8%)	16 (100.2%)<br>32 (76.1%)	>32 (8.9%)<br>>32 (-0.8%)	16 (104.6%)<br>32 (101.0%)	>32
Pal HR1-18	C0142188	>32 (9.8%)<br>>32 (15.6%)	>32 (11.5%)<br>>32 (10.5%)	32 (91.9%)<br>32 (98.8%)	>32 (7.3%)<br>>32 (1.2%)	>32 (-2.7%)<br>>32 (7.5%)	>32 (29.4%)<br>>32 (23.6%)	>32 (16.3%)<br>32 (102.7%)	>32 (15.0%)<br>>32 (49.9%)
Myr HR1-18	C0142189	>32 (11.5%)<br>>32 (16.0%)	>32 (4.5%)<br>>32 (8.1%)	>32 (4.1%)<br>>32 (7.1%)	>32 (6.9%)<br>>32 (3.8%)	>32 (0.3%)<br>>32 (1.9%)	>32 (9.1%)<br>>32 (0.0%)	>32 (-11.3%)<br>>32 (9.2%)	>32 (9.8%)<br>>32 (60.9%)
HR1-11 Palm	C0142190	>32 (11.4%)<br>>32 (8.3%)	>32 (1.3%)<br>>32 (5.6%)	>32 (3.1%)<br>>32 (6.5%)	>32 (7.4%)<br>>32 (5.4%)	>32 (-0.2%)<br>>32 (3.6%)	>32 (15.3%)<br>>32 (4.4%)	>32 (-0.8%)<br>>32 (17.3%)	>32 (24.1%)<br>>32 (29.2%)
PalHR1-11-3H3R	C0142191	32 (96.2%)<br>32 (95.6%)	>32 (5.6%)<br>>32 (-0.9%)	8 (99.4%)<br>8 (102.2%)	>32 (11.9%)<br>>32 (9.9%)	>32 (15.1%)<br>>32 (18.6%)	32 (75.1%)<br>>32 (19.7%)	32 (114.1%)<br>32 (94.6%)	>32 (7.2%)<br>>32 (19.9%)
(D)PalGR13	C0142192	8 (96.9%)<br>4 (97.3%)	32 (89.5%)<br>32 (93.2%)	4 (97.6%)<br>4 (98.0%)	16 (97.5%)<br>16 (98.7%)	2 (96.8%)<br>4 (95.5%)	4 (98.7%)<br>4 (88.9%)	2 (111.9%)<br>2 (131.9%)	>32 (8.2%)<br>>32 (31.4%)
(D)PalGR9	C0142193	>32 (12.5%)<br>16 (98.0%)	>32 (4.7%)<br>>32 (9.2%)	>32 (6.2%)<br>>32 (9.9%)	>32 (10.6%)<br>>32 (8.1%)	>32 (41.1%)<br>32 (94.2%)	>32 (16.5%)<br>>32 (9.6%)	>32 (63.3%)<br>>32 (63.1%)	>32 (12.6%)<br>>32 (48.1%)
Pal(D)GR4	C0142194	>32 (7.3%)<br>>32 (12.7%)	>32 (6.9%)<br>>32 (8.6%)	>32 (2.3%)<br>>32 (9.2%)	>32 (3.3%)<br>>32 (4.5%)	>32 (-2.2%)<br>>32 (12.4%)	>32 (22.8%)<br>>32 (10.2%)	>32 (17.4%)<br>>32 (9.2%)	>32 (14.8%)<br>>32 (15.5%)
Pal(D)GK4	C0142195	>32 (29.1%)<br>>32 (27.8%)	>32 (28.2%)<br>>32 (28.7%)	32 (91.7%)<br>32 (93.6%)	>32 (4.9%)<br>>32 (4.9%)	32 (89.0%)<br>32 (92.6%)	>32 (50.4%)<br>>32 (31.2%)	8 (95.3%)<br>16 (97.3%)	>32 (23.4%)<br>>32 (35.1%)
Pal(D)GO4	C0142196	>32 (4.9%)<br>>32 (13.2%)	>32 (6.6%)<br>>32 (8.7%)	>32 (36.2%)<br>>32 (41.8%)	>32 (7.6%)<br>>32 (2.6%)	>32 (18.5%)<br>>32 (20.7%)	>32 (32.8%)<br>>32 (6.7%)	32 (91.4%)<br>32 (94.6%)	>32 (8.4%)<br>>32 (25.2%)
PalHR1-18-3H3R-GR13	C0142197	>32 (39.2%)<br>>32 (37.5%)	>32 (4.9%)<br>>32 (9.9%)	16 (93.5%)<br>32 (98.3%)	>32 (7.3%)<br>>32 (1.7%)	>32 (25.3%)<br>>32 (39.4%)	>32 (12.3%)<br>>32 (5.1%)	>32 (-7.5%)<br>>32 (11.0%)	>32 (13.9%)<br>>32 (43.9%)
HRNR1389-1414-S-Pal	C0142198	>32 (11.0%)<br>>32 (19.6%)	>32 (8.9%)<br>>32 (11.7%)	>32 (3.6%)<br>>32 (9.2%)	>32 (7.7%)<br>>32 (2.9%)	>32 (1.9%)<br>>32 (6.7%)	>32 (52.4%)<br>>32 (7.0%)	>32 (2.5%)<br>>32 (6.5%)	>32 (21.8%)<br>>32 (34.9%)
Pal G-Dab4	C0142199	>32 (6.8%)<br>>32 (22.4%)	>32 (6.5%)<br>>32 (5.0%)	32 (95.8%)<br>32 (99.9%)	>32 (9.1%)<br>>32 (9.3%)	32 (95.0%)<br>32 (90.7%)	>32 (45.9%)<br>>32 (24.5%)	>32 (58.8%)<br>16 (100.9%)	>32 (27.8%)<br>>32 (52.9%)
Pal GR6	C0142200	>32 (14.2%)<br>>32 (12.6%)	>32 (4.8%)<br>>32 (8.4%)	>32 (12.0%)<br>>32 (12.6%)	>32 (5.0%)<br>>32 (8.7%)	16 (83.5%)<br>16 (80.7%)	>32 (24.3%)<br>>32 (62.9%)	32 (93.1%)<br>16 (95.1%)	>32 (8.4%)<br>>32 (33.4%)
Pal-Orn-GR13	C0142201	32 (96.1%)<br>32 (96.0%)	>32 (54.1%)<br>>32 (46.5%)	32 (94.7%)<br>32 (95.5%)	32 (98.1%)<br>32 (98.1%)	32 (88.0%)<br>32 (92.2%)	32 (98.9%)<br>32 (88.8%)	32 (96.4%)<br>32 (108.5%)	>32 (41.1%)<br>>32 (37.5%)
Pal GK4	C0142202	>32 (18.5%)<br>>32 (20.0%)	>32 (4.1%)<br>>32 (4.0%)	>32 (45.7%)<br>>32 (32.8%)	>32 (8.8%)<br>>32 (6.7%)	>32 (1.2%)<br>>32 (-22.2%)	16 (90.1%)<br>0.5 (97.5%)	32 (85.2%)<br>32 (89.6%)	>32 (32.9%)<br>>32 (25.9%)
S-Pal-HRNR2004-2029	C0142203	>32 (15.5%)<br>>32 (11.6%)	>32 (4.8%)<br>>32 (4.6%)	>32 (58.2%)<br>>32 (58.5%)	>32 (10.8%)<br>>32 (8.4%)	>32 (18.2%)<br>>32 (6.1%)	>32 (63.9%)<br>16 (81.1%)	4 (91.0%)<br>8 (92.9%)	22.6 (60.4%)<br>28.6 (56.9%)
Pal-K-GR13	C0142204	16 (103.3%)<br>16 (101.7%)	>32 (60.1%)<br>>32 (49.5%)	32 (100.6%)<br>32 (100.1%)	32 (100.1%)<br>32 (100.4%)	32 (98.4%)<br>>32 (56.6%)	32 (94.1%)<br>32 (98.1%)	32 (87.0%)<br>32 (91.3%)	>32 (22.7%)<br>>32 (18.6%)
Pal GR11	C0142205	8 (101.4%)<br>16 (99.9%)	>32 (40.4%)<br>32 (88.5%)	8 (97.3%)<br>8 (98.1%)	16 (96.8%)<br>16 (99.5%)	4 (99.3%)<br>4 (97.3%)	4 (97.7%)<br>8 (98.0%)	4 (95.3%)<br>4 (99.8%)	>32 (36.8%)<br>>32 (29.2%)
C-Pal-GR13	C0142220	32 (94.2%)<br>32 (93.9%)	>32 (48.3%)<br>>32 (55.3%)	32 (84.6%)<br>32 (79.4%)	32 (95.2%)<br>32 (94.9%)	4 (95.3%)<br>4 (94.5%)	2 (98.9%)<br>2 (98.8%)	0.5 (96.2%)<br>1 (99.5%)	>32 (41.6%)<br>>32 (15.1%)
8-C-Pal-GR13	C0142221	16 (101.6%)<br>32 (97.4%)	32 (92.4%)<br>32 (97.3%)	32 (90.6%)<br>32 (92.7%)	32 (97.1%)<br>32 (97.4%)	4 (98.1%)<br>4 (94.8%)	4 (99.5%)<br>4 (98.6%)	2 (93.1%)<br>2 (95.9%)	>32 (35.1%)<br>>32 (40.1%)
LCE1B 78-88	C0142222	>32 (3.8%)<br>>32 (17.4%)	>32 (0.4%)<br>>32 (6.1%)	>32 (8.4%)<br>>32 (2.9%)	>32 (9.7%)<br>>32 (8.6%)	>32 (11.2%)<br>>32 (11.2%)	>32 (18.3%)<br>>32 (5.2%)	>32 (2.7%)<br>>32 (-3.8%)	>32 (24.4%)<br>>32 (24.9%)
LCE2A 70-82	C0142228	>32 (14.8%)<br>>32 (20.1%)	>32 (3.0%)<br>>32 (6.8%)	>32 (7.6%)<br>>32 (10.3%)	>32 (8.5%)<br>>32 (7.3%)	>32 (15.6%)<br>>32 (15.3%)	>32 (10.4%)<br>>32 (9.7%)	>32 (-1.5%)<br>>32 (-8.4%)	>32 (8.6%)<br>>32 (6.6%)
LCE3A 50-60	C0142229	>32 (12.7%)<br>>32 (14.4%)	>32 (3.8%)<br>>32 (7.5%)	>32 (8.6%)<br>>32 (3.9%)	>32 (6.3%)<br>>32 (4.0%)	>32 (15.0%)<br>>32 (12.6%)	>32 (23.6%)<br>>32 (13.6%)	>32 (-4.3%)<br>>32 (-13.0%)	>32 (18.2%)<br>>32 (6.6%)
LCE3C 55-66	C0142231	>32 (7.7%)<br>>32 (11.2%)	>32 (4.5%)<br>>32 (5.7%)	>32 (7.9%)<br>>32 (18.0%)	>32 (13.8%)<br>>32 (6.2%)	>32 (12.9%)<br>>32 (5.8%)	>32 (17.8%)<br>>32 (18.5%)	>32 (-4.0%)<br>>32 (-5.1%)	>32 (26.9%)<br>>32 (24.0%)
LCE3D 55-64	C0142232	>32 (1.9%)<br>>32 (7.9%)	>32 (2.6%)<br>>32 (2.2%)	>32 (0.3%)<br>>32 (18.3%)	>32 (8.7%)<br>>32 (5.2%)	>32 (9.1%)<br>>32 (5.2%)	>32 (15.5%)<br>>32 (7.6%)	>32 (-0.6%)<br>>32 (-1.5%)	>32 (39.8%)<br>>32 (23.4%)

* Minimal inhibitory concentration (MIC) values were determined using the broth microdilution susceptibility test according to the CLSI (Clinical and Laboratory Standards Institute) guidelines in Mueller–Hinton (MH) broth on 5×10^5 CFU/mL. They were defined as the lowest peptide concentration that prevented visible bacterial growth after incubation for 18 h at 37 °C. Results are from "**C**ommunity for **O**pen **A**ntimicrobial **D**rug **D**iscovery, CO-ADD". Further details of the screening experiments are available from http://www.co-add.org/file/co-add-workflowscreeningpdf-0.

Supplementary Table 16 I Structures of CIDAMPs tested at conditions recommended by CLSI.

Name	AA-sequence
LCE-2B 74-86	SHHRPRLFHRRRH
LCE-3B 56-68	SHHRCCRSHRCRR
LCE-3C 55-67	SHHRHFRSHQCRR
LCE-3C 55-75	SHHRHFRSHQCRRQRSNSCDR
Pal-HR 1-18-HR	Pal-GRRGSGLGRSSSRGQRGSGSGR
Pal-HR-1-11-2H2R-14	Pal-RGSRSGQSSRGERR
Pal-GR13	Pal-GRRGGRGGRGRGR
Pal-GR9	Pal-GRRGGRGGR
Pal-GR4	Pal-GRGR
Pal-G-Dap4	Pal-GDapGDap
Pal-LCE-3C 55-67	Pal-SHHRHFRSHQCRR
Pal-LCE-3B 56-68	Pal-SHHRCCRSHRCRR
S-Pal-HRNR 1748-1778	SGHSSVFGQHESGSGHSSAYSQHGSGSGHFC-Pal-NH2
Pal-HR 1-18 GR13	Pal-GRRGSGLGRSSSR
Pal-HR1-18	Pal-GRHGSGLGHSSSHGQHGSGSGR
Myr-HR1-18	Myr-GRHGSGLGHSSSHGQHGSGSGR
HR-1-11-Palm	Pal-HGSRSGQSSRGERHGSSSGSSSH
Pal-HR-1-11-3H3R	Pal-RGSRSGQSSRGERRGSSSGSSSR
(D)-Pal-GR13	Pal-GR$_D$R$_D$GGR$_D$GGR$_D$GR$_D$GR$_D$
(D)-Pal-GR9	Pal-GR$_D$R$_D$GGR$_D$GGR$_D$
(D)-Pal-GR4	Pal-GR$_D$GR$_D$
(D)-Pal-GK4	Pal-GK$_D$GK$_D$
(D)-Pal-GO4	Pal-GO$_D$GO$_D$
Pal-HR-1-18-3H3R-GR13	Pal-GRRGSGLGRSSSR
HRNR 1389-1414-S-Pal	GRHGSGSGQSSGFGHHESSSWQSSGC-Pal-NH2
Pal-GDab4	Pal-GDabGDab
PalGR6	Pal-GRRGGR
Pal-OrnGR13	Pal-GOOGGOGGOGOGO
Pal-GK4	Pal-GKGK
S-Pal-HRNR2004-2029	GQHGSSSGHSSTHGQHGSASGQSSSC-Pal-NH2
Pal-K-GR13	Pal-GKKGGKGGKGKGK
Pal-GR11	Pal-GRRGGRGGRGR
C-Pal-GR13	GRRGGRGGRGRGR-C-Pal-NH2
8 C-Pal-GR13	GRRGGRG-C-Pal-GRGRGR
LCE1B 78-88	SHHRRRRSHCH
LCE2A 70-82	SHHRPRLFHRHRH
LCE3A 50-60	SHHRCRRSHRC
LCE3C 55-66	SHHRHFRSHQCR
LCE3D 55-64	NHHRRHHRCR

Residues cationic at pH 7, cationic only at pH < 6, and anionic at pH 7 are colored blue, green and magenta, respectively. Dab:L-2,4-Diaminobutyric acid, Dap:L-2,3-Diaminopropionic acid.

Supplementary Table 17 I HRNR-primer-sequences used in this study

Name	Primer Sequence	A.-T.	D.-T:
pSu-hr3-a-2576-f (BsaI)	AAGGTCTCAAGGTGGCTTTGGTCACCACGAGT	60°C	79,1°C
pSu-hr3-b-2591-f (BsaI)	AAGGTCTCAAGGTAGTCAGCATGGGTCTGGCT	60°C	77,1°C
pSu-hr3-c-2638-f (BsaI)	AAGGTCTCAAGGTCAGTCTTCGGGCCACGGC	62°C	82,3°C
pSu-hr3-d-2656-f (BsaI)	AAGGTCTCAAGGTGGCCGACATGGGTCCGGT	62°C	83,7°C
pSu-hr3-e-2658-f (BsaI)	AAGGTCTCAAGGTCATGGGTCCGGTTTGGGG	60°C	81,6°C
pSu-hr3-a-2707-r (BamHI)	AAGGATCCTTAGTAAGCAGAGGAATGTCCTG	60°C	70,8°C
pSu-hr3-b-2684-r (BamHI)	AAGGATCCTTAATATGGGCCACGGCTGGAA	60°C	77,4°C
pSu-hr3-d-2618-r (BamHI)	AAGGATCCTTATCGTTCACCCCTAGATGACTG	64°C	73,5°C
pSu-hr3-e-2609-r (BamHI)	AAGGATCCTTATCTAGAGCCGTGTTGTCCGT	62°C	73,9°C
pSu-hr3-f-2644-r (BamHI)	AAGGATCCTTAACGGGAGCCAGACCCATG	60°C	77,4°C
hr3-2662G-f	GTCCGGT**GGC**GGGCACTCCTCCAG	72°C	82,1°C
hr3-2662G-r	GTGCCC**GCC**ACCGGACCCATGTCG	72°C	84,9°C
hr3-2629G-f	CAC**GG**TGGTCAGCATGGGTCTGG	68°C	76,7°C
hr3-2629G-r	GCTGACCA**CC**GTGGGAAGACGAACC	74°C	77,6°C
hr3-2603G-f	CTGGC**GG**CGGACAACACGGCTC	68°C	80,3°C
hr3-2603G-r	TGTTGTCCG**CC**GCCAGAGGAGTG	68°C	78,1°C
Su3-hr3-2684G-r (BamHI)	AAAGGATCCTCAA**CC**TGGGCCACGGCTGGAAGA	62°C	83,8°C
pESu3-hrnr-3-f (FokI)	AAGGGGATGAGCATGATTAGGTAAACTCCTACAAGGCGTCATC	62°C	80,3°C
pESu3-hrnr-244-r (XhoI)	AAGGCTCGAGTCAGTAACCAGAGGACTGCCC	58°C	77,8°C
pESu3-hrnr-82-f (FokI)	AAGGGGATGAGCATGATTAGGTGTTCAGGCTCGTAATAAAATC	58°C	79,0°C
pESu3-hrnr-245-f (FokI)	AAGGGGATGAGCATGATTAGGTAGTCAGCATGGATCTGGC	56°C	81,8°C
pESu3-hrnr-478-r (XhoI)	AAGGCTCGAGTCAGTAACCAGAGGAATGCTCT	56°C	75,1°C
HRNR-2591-f-CyBr (SbfI)	AACCCTGCAGGAGATGAGTCAGCATGGGTCTGGCT	74°C	83,6°C

A.-T.: Annealing temperature; D.-T.: Detection temperature. The complementary coding sequence is underlined.

REFERENCES

93. Schechter, I. & Berger, A. On the size of the active site in proteases. I. Papain. *Biochem. Biophys. Res. Commun.* **27,** 157–162 (1967).
94. Deng, X., Eickholt, J. & Cheng, J. PreDisorder: ab initio sequence-based prediction of protein disordered regions. *BMC Bioinformatics* **10,** 436 (2009).
95. McGuffin, L. J. Intrinsic disorder prediction from the analysis of multiple protein fold recognition models. *Bioinforma. Oxf. Engl.* **24,** 1798–1804 (2008).
96. Ward, J. J., Sodhi, J. S., McGuffin, L. J., Buxton, B. F. & Jones, D. T. Prediction and functional analysis of native disorder in proteins from the three kingdoms of life. *J. Mol. Biol.* **337,** 635–645 (2004).
